# Identification of “sarsasapogenin-aglyconed” timosaponins as novel Aβ-lowering modulators of amyloid precursor protein processing†
†Electronic supplementary information (ESI) available: ESI includes experimental procedures for biology and chemistry experiments, synthetic figures, NMR data and mass spectroscopic analysis. See DOI: 10.1039/c5sc02377g


**DOI:** 10.1039/c5sc02377g

**Published:** 2016-01-22

**Authors:** Lai-King Sy, Chun-Nam Lok, Juan-Yu Wang, Yungen Liu, Lu Cheng, Pui-Ki Wan, Chi-Ting Leung, Bei Cao, Wai-Lun Kwong, Raymond Chuen-Chung Chang, Chi-Ming Che

**Affiliations:** a Department of Chemistry , The University of Hong Kong , Chemical Biology Centre , 8/F., The Hong Kong Jockey Club Building for Interdisciplinary Research, 5, Sassoon Road, Pokfulam , Hong Kong , China . Email: cmche@hku.hk ; Fax: +852-28571586 ; Tel: +852-28592154; b Neurodegenerative Diseases Laboratory , School of Biomedical Sciences , LKS Faculty of Medicine , Research Centre of Heart, Brain and Hormone, and Healthy Aging , LKS Faculty of Medicine , State Key Laboratory of Brain and Cognitive Sciences , The University of Hong Kong , China

## Abstract

The inhibition of amyloid β (Aβ) peptide production is a key approach in the development of therapeutics for the treatment of Alzheimer's disease (AD). We have identified that timosaponins consisting of sarsasapogenin (SSG) as the aglycone can effectively lower the production of Aβ peptides and stimulate neurite outgrowth in neuronal cell cultures. Structure–activity relationship studies revealed that the *cis*-fused AB ring, 3β-configuration, spiroketal F-ring and 25*S*-configuration of SSG are the essential structural features responsible for the Aβ-lowering effects and neurite-stimulatory activity. New synthetic derivatives that retain the SSG scaffold also exhibited an Aβ lowering effect. Treatment of cells with timosaponins led to modulation of amyloid precursor protein (APP) processing through the suppression of β-cleavage and preferential lowering of the production of the 42-amino acid Aβ species (Aβ_42_) without affecting another γ-secretase substrate. The SSG and “SSG-aglyconed” timosaponins also penetrated brain tissue and lowered brain Aβ_42_ levels in mice. Our studies demonstrate that timosaponins represent a unique class of steroidal saponins that may be useful for the development of AD therapeutics.

## Abstract 2

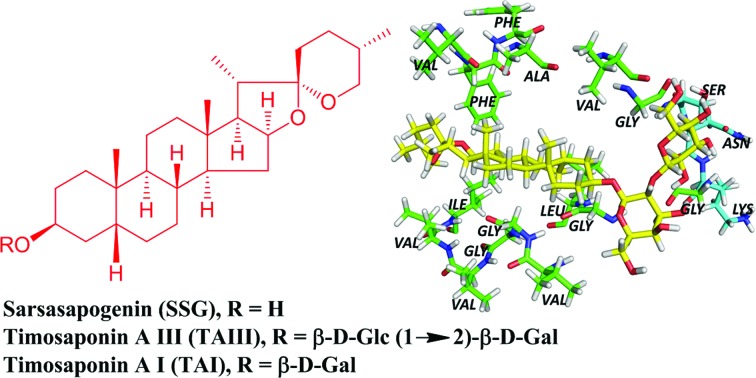
The “sarsasapogenin-aglyconed” timosaponins are Aβ lowering agents that may be useful for the development of Alzheimer’s disease therapeutics.

## Introduction

Alzheimer's disease (AD) is a neurodegenerative disorder that starts with a decline in short-term memory and progresses to the loss of cognition and executive functions. The pathology of AD is characterized by synaptic loss, neuronal death, frequent deposition of phosphorylated tau proteins and Aβ aggregation within the brain.^1^ Although the underlying cause of AD is complex, the accumulation of Aβ within the brain appears to play a pivotal role in the onset and progression of the disease.^2^

Aβ is generated from the proteolysis of amyloid precursor protein (APP) during aging or in subjects with an inherited cause of AD.^3^ APP is a transmembrane protein whose proteolysis is mediated by α-, β- and γ-secretases that cleave APP at specific sites.^3^ The amyloidogenic process first involves the cleavage of APP to create a c-terminal fragment (CTF), known as β-CTF, which is subsequently cleaved by a multiprotein γ-secretase complex to produce different lengths of Aβ peptides such as Aβ_38_, Aβ_40_ and Aβ_42_. Genetic and mechanistic data strongly suggest that the accumulation of amyloidogenic Aβ_42_ peptide results in the formation of toxic oligomers and/or fibrils. Accordingly, Aβ_42_-lowering compounds that target the β- and/or γ-cleavage processes represent a promising strategy for therapeutic intervention in AD.^4^,^5^ γ-Secretase inhibitors (GSI) initially emerged as effective Aβ-lowering agents, but the side effects resulting from non-specific inhibition of other vital γ-secretase substrates, such as Notch, have complicated the development of these inhibitors.^6^ With evidence for the more specific role of Aβ_42_ in amyloidogenesis, current approaches to AD drug development are focused on γ-secretase modulators (GSM) that preferentially lower Aβ_42_ without affecting the action of γ-secretase on general APP processing and the cleavage of other substrates.^7^ Modulation of β-secretase is also considered to be a viable strategy for lowering Aβ production.^8^ A mutation in APP that hinders β-cleavage and lowers the production of Aβ by 40% was demonstrated to be protective against AD and age-related cognitive decline,^9^ supporting the idea that a moderate reduction of Aβ in humans is favourable for AD treatment or prevention.

**Fig. 1:**
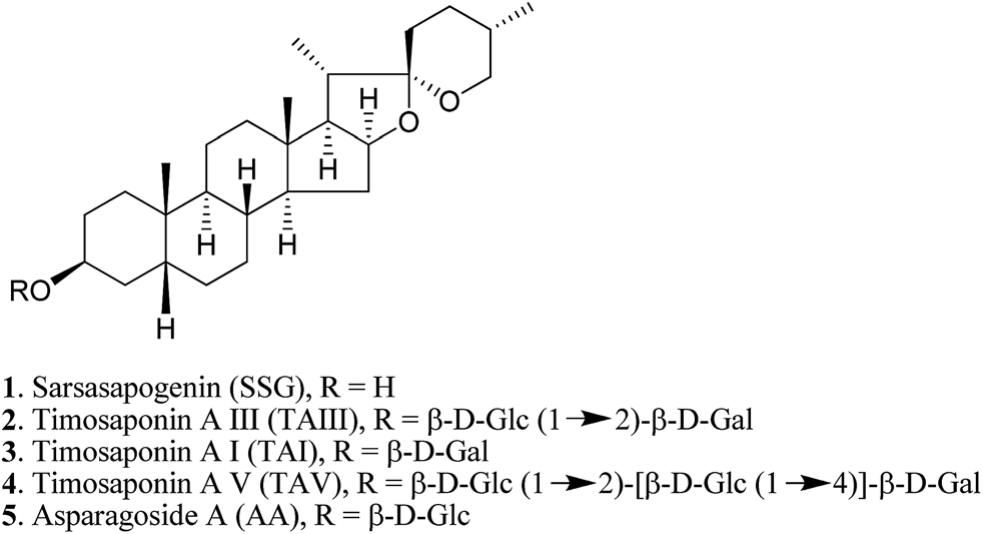
Chemical structures of effective Aβ_42_-lowering agents in N2A-APPswe cells.

Natural products provide opportunities for the development of anti-AD pharmaceuticals (ESI Table S1†). For example, green tea polyphenols, ginsenosides and resveratrol have all been shown to exhibit promising anti-amyloidogenic effects.^10^–^12^ In the course of studying the pharmacological properties of medicinal saponins from natural products, we found that timosaponins consisting of sarsasapogenin (SSG, **1**) (Fig. 1) as the aglycone, including timosaponin A III (TAIII, **2**) isolated from the rhizome of *Anemarrhena asphodeloides* Bge. (Liliaceae), could effectively lower Aβ production. We have previously identified TAIII's anti-cancer and autophagy-inducing properties.^13^,^14^ Oral administration of SSG, TAIII and other timosaponins were shown to improve memory dysfunction in animal models of dementia^15^–^18^ despite the chemical structural differences between these compounds. Total saponins from *Anemarrhena asphodeloides* Bge. were reported to ameliorate diabetes-associated cognitive decline in rats and mediate Aβ decreases in brain.^19^ In the present study, we show that SSG (**1**) and other timosaponins (**2–5**, Fig. 1) specifically exhibit Aβ-lowering activities and their actions are akin to GSM,^7^ which preferentially lowers Aβ_42_ peptide production. We propose a model by which the timosaponins may bind to the steroid binding site of APP,^20^ possibly modulating the APP secretase properties. Importantly, SSG and several timosaponins showed brain penetration and Aβ_42_-diminishing activities *in vivo*.

## Results and discussion

### Synthesis of timosaponins, SSG analogues and SSG derivatives

*A. asphodeloides* is a medicinal herb that is rich in structurally closely related timosaponins whose isolation and purification are exceptionally difficult.^17^ Chemical synthesis, on the other hand, is a feasible strategy to obtain timosaponins that are of low natural abundance or commercially unavailable. Preparation of steroidal timosaponins (**2–5**) (Fig. 1) with different sugar lengths was achieved by reacting aglycone **1** with different glycosyl donors (ESI Fig. S1–S3†).

**Fig. 2:**
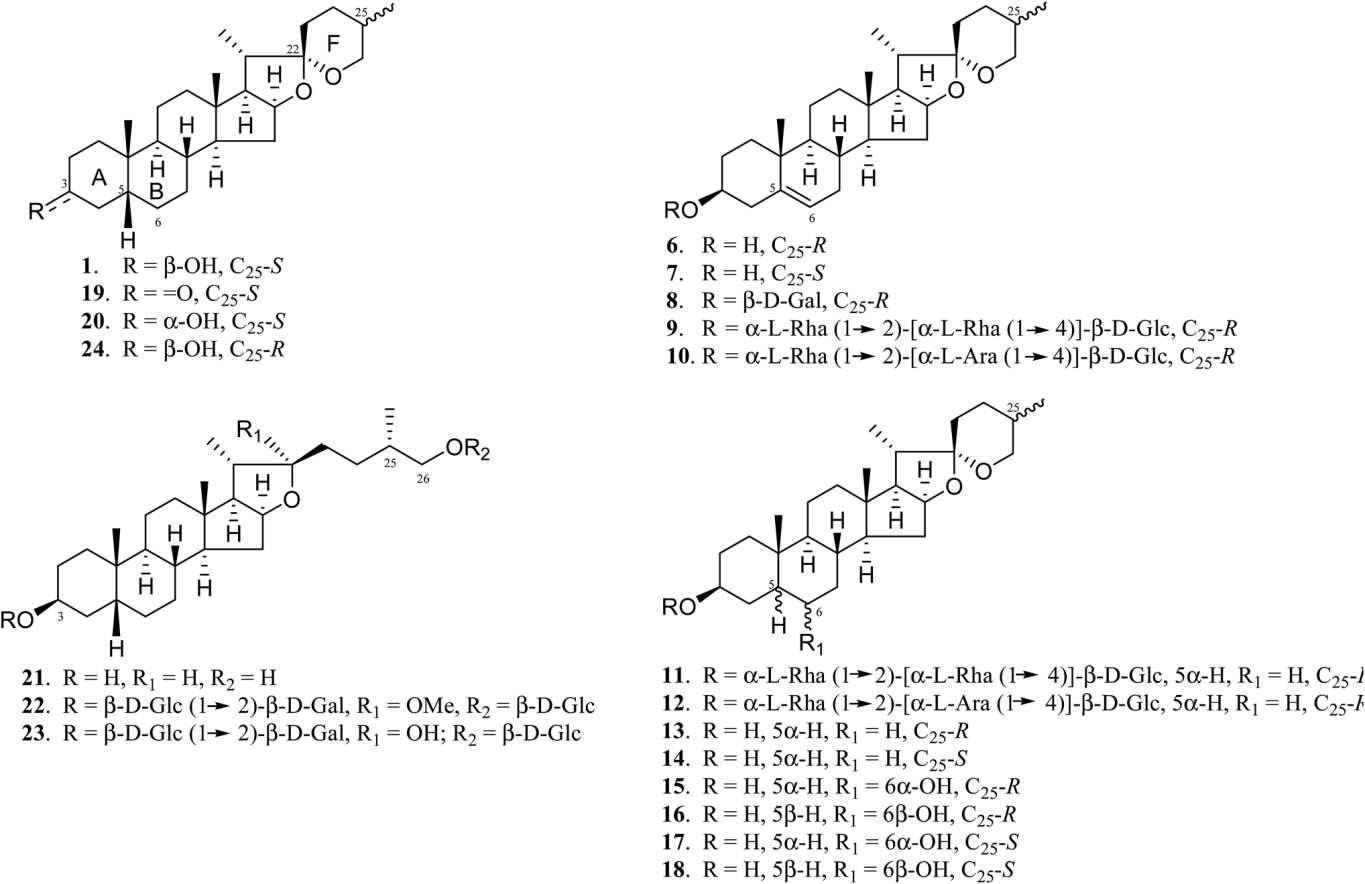
Chemical structures of SSG analogues.

The chemical structure of the aglycone SSG (**1**) is rather unique in naturally-occurring sapogenins due to its structural characteristics, including a *cis*-fused AB ring (or 5β-configuration), 3β-configuration, spiroketal F-ring and 25*S*-configuration.^21^ In this study, a series of SSG analogues (**6–24**) (Fig. 2) differing in these structural features were employed to study the structure–activity relationship (SAR) in the Aβ-lowering activities. The linkage of AB rings in steroidal sapogenins can be *cis*-, *trans*- or Δ^5^-double bonded. Δ^5^-Double bonded diosgenin (**6**) and yamogenin (**7**) were studied in addition to “diosgenin-aglyconed” saponins, including synthesized capsicoside A_3_ (**8**) (ESI Fig. S4†), dioscin (**9**) and polyphyllin D (**10**). Hydrogenation of saponins **9** and **10** generated AB ring *trans*-fused dihydrodioscin (**11**) and dihydropolyphyllin D (**12**), respectively (ESI Fig. S5†). Similarly, hydrogenation of **6** and **7** produced AB ring *trans*-fused tigogenin (**13**) and neotigogenin (**14**), respectively (ESI Fig. S6†). Functionalization at Δ^5^ of **6** or Δ^5^ of **7** led to production of a pair of isomers for each compound: 5α-H, 6α-OH diosgenin (**15**), 5β-H, 6β-OH diosgenin (**16**) and 5α-H, 6α-OH yamogenin (**17**), 5β-H, 6β-OH yamogenin (**18**) (ESI Fig. S7†).^22^ The hydration products **15** and **17** are AB ring *trans*-fused, while the new compounds **16** and **18** are AB ring *cis*-fused.

Compound **1** has a secondary β-OH at C_3_. Oxidation of **1** to sarsasapogenone (**19**) followed by reduction gave episarsasapogenin (**20**) (ESI Fig. S8†).^23^

SSG **1** has heterocyclic rings E and F fixed at C_22_ in which the F-spiroketal ring appears to be a crucial moiety in bioactive saponins^21^ (Fig. 2). Reductive cleavage of the F-ring in **1** gave dihydrosarsasapogenin (dSSG, **21**) with a terminal OH group^24^ (ESI Fig. S9†). Timosaponin B I (TBI, **22**) and timosaponin B II (TBII, **23**) are two examples of “dSSG-aglyconed” saponins with two sugar chains substituted at C_3_ and C_26_. The contribution of the C_25_*S*-configuration of **1** in decreasing Aβ_42_ was also investigated by comparing the activity of compound **16** and its epimer, smilagenin (**24**), both of which have a C_25_*R*-configuration.

**Fig. 3:**
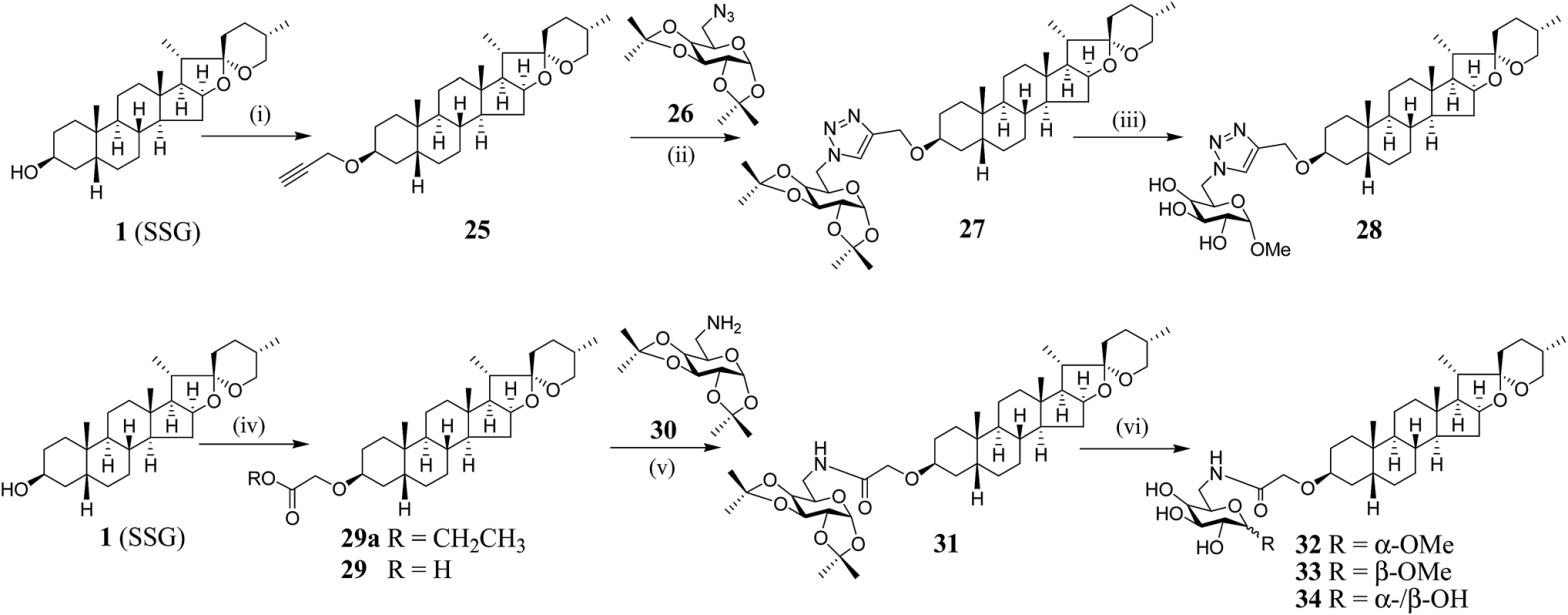
Synthesis of SSG derivatives. (i) Propargyl bromide, NaH, DMF, rt, 3 d, 46%; (ii) CuSO_4_·5H_2_O, ascorbic acid, ^*t*^BuOH/DMSO (4 : 1, v/v), rt, 24 h, 63%; (iii) (a) MeOH-35% HCl (1 : 1), 40 °C, 3 h; (b) 1 M HCl in MeOH, rt, overnight, 58% (over 2 steps); (iv) (a) ethyl diazoacetate, Rh_2_(OAc)_4_, CH_2_Cl_2_, 40 °C, 3 h; (b) K_2_CO_3_, MeOH–H_2_O (5 : 1), reflux, 5 h; (c) HCl, **29**: 80% (over 3 steps); (v) DIC, HOBt, CH_2_Cl_2_, rt, 48 h, 92%; (vi) MeOH-35% HCl (1 : 1), 40 °C, 3 h, **32**: 24%; **33**: 16%, **34**: 38%. DMF = dimethylformamide; DMSO = dimethyl sulfoxide; DIC = *N*,*N*-diisopropylcarbodiimide; HOBt = hydroxybenzotriazole.

We have synthesized new SSG derivatives (**28–29**, **32–34**) in an attempt to modify the biological activity and/or bioavailability (Fig. 3).^25^,^26^ Reaction of **1** with propargyl bromide yielded **25**, containing a propargyl group, which can be linked to other moieties or probes *via* click chemistry. By reacting **25** with acetonide-protected α-galactose azide (**26**), *via* Cu(i)-catalysed click chemistry, triazole SSG (**27**) was obtained. Removing the acetonide protecting groups of **27** yielded α-OMe triazole SSG (**28**) (ESI Fig. S10†). The relatively harsh reaction conditions (strong base NaH and long reaction time) used in the preparation of **25** was also attempted for the preparation of carboxylate ethereal SSG (**29a**) by reacting compound **1** with methyl bromoacetate, but without success. Compound **1** was then reacted with diazoacetate, in the presence of Rh_2_(OAc)_4_ as a catalyst, to obtain **29a** in good yield *via* carbene insertion under mild conditions. Hydroxylation of the carboxylate **29a** in alkaline conditions, followed by neutralization, produced the ethereal SSG (**29**) (ESI Fig. S11†). The carboxylic acid of **29** serves as a useful linkage for coupling with other moieties under mild conditions. Reaction of **29** with acetonide-protected α-galactose amine (**30**) gave amide SSG (**31**). The acetonide protecting groups of **31** were then removed by acid hydrolysis to yield α-OMe SSG (**32**), β-OMe SSG (**33**) and a mixture of α- and β-OH SSG (**34**) (ESI Fig. S12†).

### Aβ_42_-lowering activities

Using Neuro-2A neuroblastoma cells stably transfected with APP with the AD-linked Swedish mutation (N2A-APPswe) as a cell culture model of Aβ production,^27^ it was found that the SSG **1** treatment modestly decreases Aβ_42_ production with an IC_50_ of 53 μM (Table 1). Treatment with timosaponins (**2–5**) markedly lowered Aβ_42_ production when compared to the aglycone SSG. SSG analogue **18**, in addition to the newly synthesized SSG derivatives **28–29** and **32–34**, also showed slight to moderate improvement in Aβ_42_-lowering activity when compared to **1** (Table 1). In rat primary cortical neuronal cultures, which produce and secrete low levels of Aβ, chronic exposure to compounds **1–3** also resulted in a moderate diminishment of Aβ_42_ levels in the medium (Table 2).

**Table 1:** IC_50_ values of timosaponins, SSG analogues and SSG derivatives in lowering Aβ_42_ production in N2A-APPswe cells. Data represent mean ± standard deviation; *n* ≥ 3

Compound	IC_50_ (μM)	Compound	IC_50_ (μM)
SSG (**1**)	53.0 ± 9.0	5α-H, 6α-OH yamogenin (**17**)	>100
TAIII (**2**)	2.3 ± 0.2	5β-H, 6β-OH yamogenin (**18**)	45.0 ± 4.0
TAI (**3**)	6.1 ± 2.8	Sarsasapogenone (**19**)	50.0 ± 5.0
TAV (**4**)	4.2 ± 1.2	Episarsasapogenin (**20**)	>100
AA (**5**)	6.0 ± 1.4	Dihydrosarsasapogenin (**21**)	>100
Diosgenin (**6**)	>100	Timosaponin B I (**22**)	>100
Yamogenin (**7**)	>100	Timosaponin B II (**23**)	>100
Capsicoside A_3_ (**8**)	>100	Smilagenin (**24**)	>100
Tigogenin (**13**)	>100	α-OMe triazole SSG (**28**)	6.5 ± 2.1
Neotigogenin (**14**)	>100	Ethereal SSG (**29**)	27.0 ± 8.0
5α-H, 6α-OH diosgenin (**15**)	>100	α-OMe SSG (**32**)	7.2 ± 2.2
5β-H, 6β-OH diosgenin (**16**)	>100	β-OMe SSG (**33**)	9.3 ± 3.5
		α-, β-OH SSG (**34**)	7.3 ± 4.0

**Table 2:** Effects of SSG, TAIII and TAI on Aβ_42_ levels in conditioned medium of rat primary cortical neuron culture upon 5-day incubation. A low concentration of SSG was used owing to the compound's insolubility in the reduced serum medium for the neuronal culture. Data represent mean ± standard deviation; *n* = 3

Compound	Aβ_42_ reduction (%)
SSG (**1**) (5 μM)	25 ± 4
TAIII (**2**) (5 μM)	42 ± 9
TAI (**3**) (10 μM)	28 ± 5

### Structure–activity relationship

#### The role of the sugar chain in timosaponins

Treatment of N2A-APPswe cells with monosaccharide timosaponin A I (TAI, **3**) and disaccharide TAIII (**2**) showed an improved reduction of Aβ_42_ when compared to aglycone SSG **1** (Table 1), indicating that the presence of the sugar chain is beneficial in lowering Aβ_42_ levels. However, trisaccharide timosaponin A V (TAV, **4**), reported for the first time, showed no further lowering effect (IC_50_ = ∼4.2 μM) when compared to disaccharide **2** (IC_50_ = ∼2.3 μM). In view of the importance of hydrophobicity to the cell permeability of the compounds to be tested, timosaponins with longer sugar chains were not considered in this study.^28^,^29^

Timosaponins **2–4** are galactosyl derived and exhibit prominent Aβ_42_-level reducing effects. Asparagoside A (AA, **5**), a glucosyl-derived timosaponin (Fig. 1), shows a comparable Aβ_42_-lowering effect (IC_50_ = ∼6.0 μM) to galactosyl **3** (IC_50_ = ∼6.1 μM), revealing that the nature of the monosaccharide coupled to aglycone **1** has a negligible effect on Aβ_42_ levels.

The aforementioned enhancement in lowering Aβ_42_ production by the sugar chains in timosaponins has not been observed in the steroidal aglycone diosgenin **6** and its galactosyl product **8** (Table 1). Taken together with the insignificant Aβ_42_-lowering effects exhibited by diosgenyl saponins (data not shown) **9** and **10** (Δ^5^ double bond) and their corresponding hydrogenated products **11** and **12** (both AB *trans*-fused rings), it is suggested that aglycone **1** is critical in lowering Aβ_42_ production. The structural features associated with **1**, including the AB-fused ring and the F-ring, were subjected to investigation and the findings are discussed below.

#### The roles of the AB-fused ring and the C_3_ configuration in SSG

Diosgenin **6** and yamogenin **7**, both having Δ^5^ double bonds and different configurations at C_25_ (*R*- for **6** and *S*- for **7**), showed no effect in Aβ_42_ lowering when compared to **1**. Their respective hydrogenated products **13** and **14**, both of which have AB rings *trans*-fused, were also ineffective (Table 1). In addition, only compound **18** (AB rings *cis*-fused) from the 4 hydrated products **15–18** caused reduction of Aβ_42_ levels (Table 1), indicating that the *cis*-fused AB ring (or 5β) in **1** is biologically significant in decreasing Aβ_42_ production.

Compound **19**, with a ketone functionality at C_3_, showed a comparable effect to **1** in Aβ_42_ lowering, while compound **20**, with a C_3_ α-OH, was inactive (Table 1), implying that the 3α-configuration is unfavourable for Aβ_42_ lowering.

#### The roles of the F-spiroketal ring and the C_25_ configuration in SSG

Steroidal sapogenins, including spirostane-, furostane- and cholestane-types, are widely distributed in the plant kingdom.^30^ SSG **1**, which has an intact spiroketal F-ring, is an example of the spirostane-type (Fig. 2). As mentioned above, this compound exhibits an Aβ_42_-lowering effect. However, such an effect was not observed for the F-ring cleaved, furostane-type dSSG **21** (IC_50_ > 100 μM). In addition, timosaponin B I (**22**) and timosaponin B II (**23**), possessing dSSG as the aglycone, are also ineffective in lowering Aβ_42_ (Table 1). Timosaponin B II (**23**), however, has been shown to inhibit the up-regulation of β-secretase induced by ferric chloride in rat retina.^31^ Ergosterol (structure not shown) structurally resembles a cholestane-type aglycone. This sterol also elicited no attenuation of Aβ_42_ production (IC_50_ > 100 μM). It is therefore concluded that the F-spiroketal ring in **1** should remain intact for Aβ_42_-lowering activity.

Smilagenin **24** and 6β-OH substituted **16** were ineffective in lowering Aβ_42_ (Fig. 2 and Table 1). Thus, one may envisage that the 25*S*-configuration in **1** is one of the vital structural factors contributing to the compound's Aβ-lowering activities.

Collectively, the SSG moiety is essential for Aβ_42_-lowering activity. Notably, the Aβ_42_-lowering effect of SSG is significantly enhanced when the compound is glycosylated at C_3_ to obtain timosaponins **2–5** or carboxylated at C_3_ to obtain SSG derivatives **28–29** and **32–34** (Fig. 1, 3 and Table 1). It is suggested that chemical modification at the C_3_ position of **1** is an appealing approach for the generation of versatile SSG derivatives with anti-amyloidogenic effects and thus timosaponins herein represent a class of interesting saponins noteworthy of further investigation related to Aβ_42_-lowering activities.

### Biochemical mechanisms

#### Timosaponins modulate APP processing with suppression of β-cleavage

**Fig. 4:**
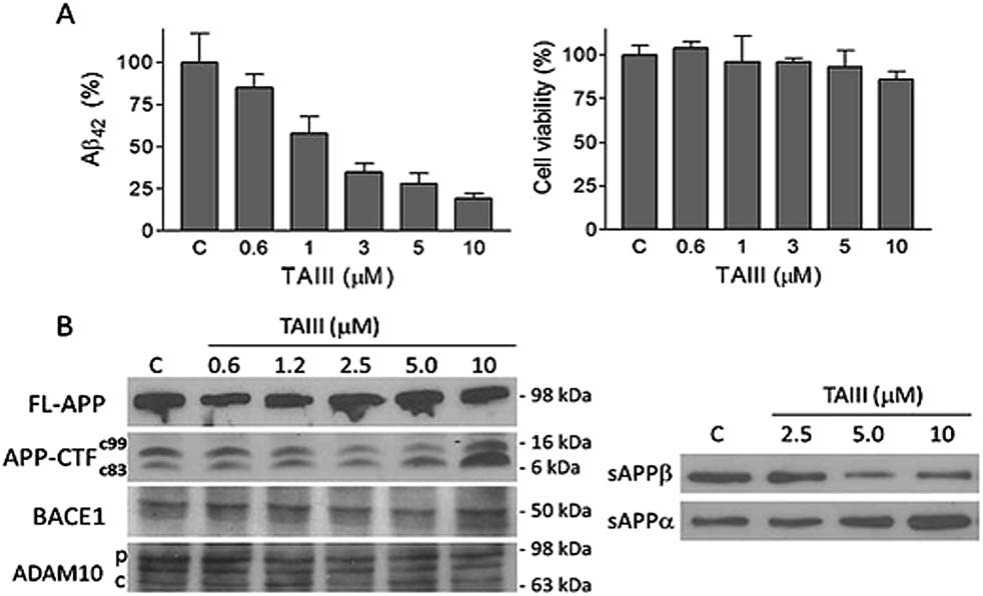
Effects of TAIII on Aβ_42_ production and APP processing in N2A-APPswe cells. Cells were treated with various concentrations of TAIII or DMSO vehicle as control (C) for 18 h. (A) The Aβ_42_ concentrations in the conditioned medium were determined by ELISA. The cell viability was determined by MTT assay. Data represent means ± standard deviation; *n* = 3. (B) The expression of full length (FL), CTF (C99, C83), BACE1 and ADAM10 (p: precursor form; c: cleaved form) in cell lysates and secreted APP (sAPPα and sAPPβ) in the conditioned medium were examined by immunoblot.

**Fig. 5:**
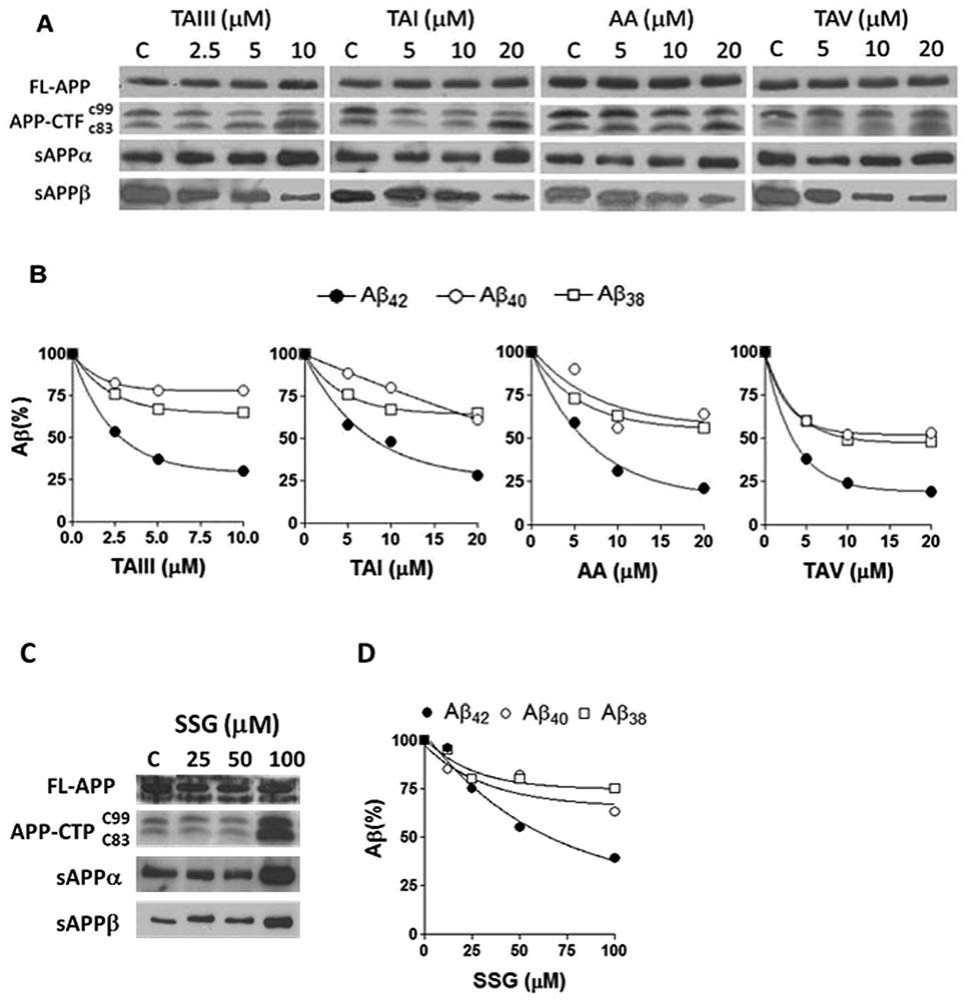
Effects of timosaponins and SSG on APP processing and Aβ profiles in N2A-APPswe cells. (A) Cells were treated with various concentrations of TAIII/TAI/AA/TAV and DMSO vehicle as control (C) for 18 h. The expression of full length (FL) and CTF in cell lysates and the levels of secreted APP (sAPPα and sAPPβ) in the conditioned medium were examined by immunoblot. (B) The profiles of Aβ_42_, Aβ_40_ and Aβ_38_ in the conditioned medium were determined by ELISA. (C) Effects of SSG on APP processing. (D) Effects of SSG on Aβ profiles.

We have investigated whether the timosaponins interfere with APP processing by immunoblot analysis. Treatment of N2A-APPswe cells with TAIII did not elicit a change in the expression of full length APP, but resulted in the alteration of the expression of the CTF and secreted APP fragments (sAPP) (Fig. 4). In general, TAIII treatment decreased the levels of the β-secretase-cleaved CTF (C-99) and secreted APPβ in a concentration-dependent manner. Elevated concentrations (10 μM) of TAIII increased the α-secretase-cleaved CTF (C-83) and secreted APPα (Fig. 4). These changes in the expression of APP cleavage products are indicative of suppression of the amyloidogenic β cleavage and/or enhancement of the α cleavage, which is non-amyloidogenic and is competitive to the former. Other timosaponins (TAI/TAV/AA) that exhibited effective Aβ-lowering activities also elicited changes in APP cleavage products similar to that of TAIII (Fig. 5A). TAIII treatment did not affect the expression of the β-secretase BACE1 (Fig. 4), β-secretase activities in the cell extracts or the activities of purified BACE1. Furthermore, TAIII treatment also did not alter the expression of ADAM-10, an α-secretase that is activated by proteolytic cleavage (Fig. 4). Thus, these results suggest that the Aβ-lowering effects mediated by timosaponins are unlikely to be due to changes in the enzyme activities of α- and β-secretases. However, allosteric modulation of the APP processing complexes may be a possible cause for the alterations of APP cleavage elicited by the timosaponins.

We also studied the effect of SSG on the Aβ production and APP processing (Fig. 5C & D). The Aβ-lowering activity of SSG was weaker than those of timosaponins generally. Treatment of N2A-APPswe cells with SSG at 50 μM and 100 μM for 18 h caused a decrease in Aβ_42_ by 55% and 40%, respectively. Interestingly, treatment of cells with SSG at 100 μM increased both α-CTF and β-CTF expression with marked increase in the sAPPα levels. There was no change in β-secretase activity in the protein extracts from SSG-treated cells compared to that from untreated cells. Though the mechanism accounting for the difference between timosaponins and SSG in APP processing remains to be elucidated, it was concluded that the sugar moiety appears to play a role in modifying the effects of the timosaponins on APP processing.

#### Timosaponins preferentially lower Aβ_42_ production similar to the action of GSM

Aβ species of variable lengths are generated upon cleavage of the CTFs of APP by γ-secretase; these Aβ profiles can be used for predicting the mechanisms of action of drugs that act on the APP-secretase complexes.^4^,^5^ As revealed by ELISA specific for individual Aβ species, treatment of cells with timosaponins (TAIII/TAI/TAV/AA) effectively lowered the levels of secreted Aβ_42_ while having a much smaller effect on Aβ_40_ and Aβ_38_ (Fig. 5B). The timosaponins' preferential effects on Aβ_42_, over the shorter forms of Aβ species, resemble the results of γ-secretase modulation.^4^–^7^ Currently, identification of novel GSM is of considerable interest in the development of AD therapeutics, because Aβ_42_ oligomers or fibrils are considered to be the most toxic Aβ species in AD pathology.

**Fig. 6:**
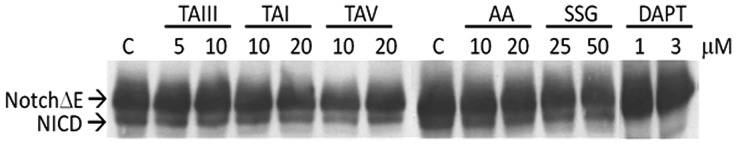
Effects of timosaponins on Notch cleavage by γ-secretase. N2A-APPswe cells were transfected with myc-tagged NotchΔE construct, which is constitutively cleaved by γ-secretase to generate NICD. Cells were then treated with DMSO control (C); the indicated concentrations of timosaponins or DAPT (as a positive control for γ-secretase inhibition) for 18 h and the expression of NotchΔE and NICD were examined by immunoblot analysis.

GSM are expected to selectively act on the APP complexes, without inhibiting the cleavage activity of γ-secretase on other physiological substrates.^6^,^7^ To further investigate the specificity of the impact of timosaponins on γ-secretase-mediated protein processing, the effects of timosaponins on the γ-secretase-mediated cleavage of the transmembrane receptor Notch1 were examined (Fig. 6). γ-Secretase-catalysed cleavage of Notch releases the Notch intracellular domain (NICD), which regulates developmental gene transcription.^32^ In cells expressing a Notch1 variant containing transmembrane and intracellular domains (NotchΔE), the NICD is constitutively present due to γ-secretase activity (Fig. 6). Treatment of cells with a γ-secretase inhibitor DAPT completely blocked the production of NICD, while treatment of cells with timosaponins at concentrations that effectively lower Aβ levels did not affect NICD levels. Thus, the timosaponins selectively interfere with Aβ production without altering Notch1 processing.

#### Timosaponins stimulate neurite outgrowth

**Fig. 7:**
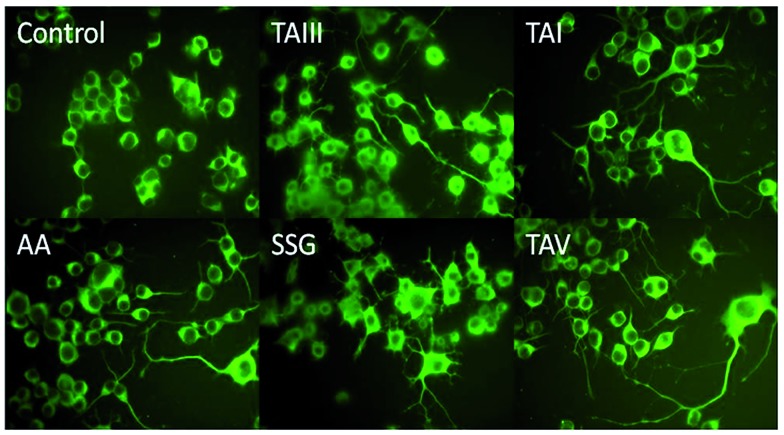
Effects of timosaponins and SSG on neurite outgrowth of Neuro-2A cells. Cells were treated with DMSO (control), TAIII (5 μM), TAI (10 μM), AA (10 μM), SSG (25 μM) and TAV (10 μM) for 18 h, stained with a monoclonal antibody raised against type III β-tubulin and examined by fluorescence microscopy.

Intriguingly, treatment of cells with SSG (**1**) and timosaponins (**2–5**) also markedly stimulated neurite outgrowth at concentrations that lower Aβ production, as revealed by type III β-tubulin immunostaining (Fig. 7). The neurite outgrowth stimulation was not shared by other steroidal saponins (*e.g.*, **8–12**) or SSG analogues (*e.g.*, **6**, **7**, **13**, **14**, **20**, and **24**) investigated in this study. Stimulation of neurite outgrowth is considered to be a favourable property in pharmaceuticals designed to ameliorate AD, which is characterized by neuronal loss.^1^ Neurite outgrowth is a complex neuronal process that is, in part, modulated through the interaction of membrane-bound and/or secreted forms of APP with proteins of axonal and dendritic growth machinery.^33^,^34^ The mechanism by which timosaponins exhibit neurite outgrowth stimulatory property remains to be elucidated, but is perhaps related to its APP modulating properties, which may tip the balance toward neurite growth and branching.

#### APP as a potential molecular target of timosaponins

**Fig. 8:**
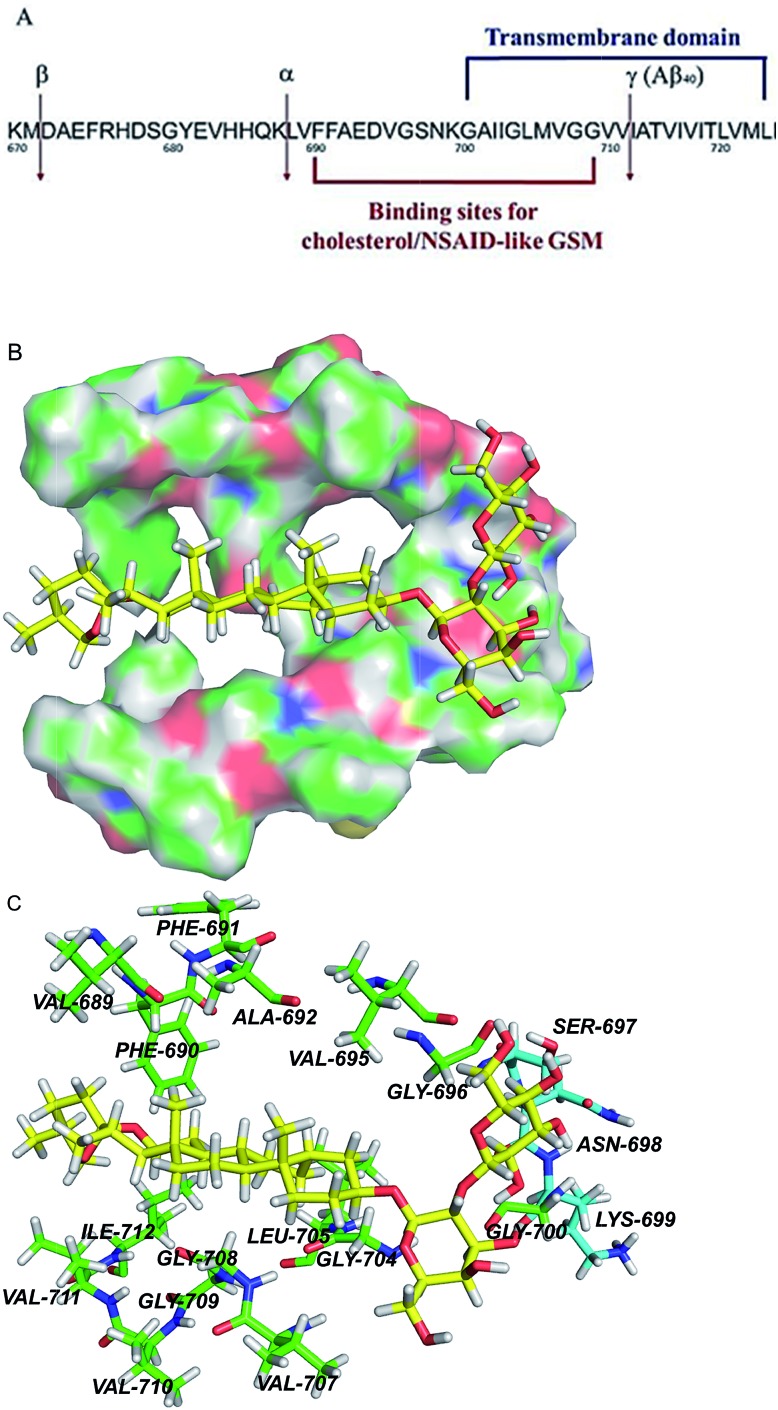
APP transmembrane domain as a potential molecular target of timosaponins. (A) An overview of the portion of APP that is cleaved by α-, β-, and γ-secretases. Also shown are the juxtamembrane and transmembrane regions harbouring binding sites for NASID-like GSM^34^ and cholesterol.^20^ Numbering is according to the full length of APP770. (B) QM/MM calculations of TAIII binding to the transmembrane domain of APP. The surface representation of the transmembrane region of APP, showing the calculated binding pose of TAIII. (C) The hydrophobic residues and polar residues around TAIII are portrayed with stick display mode. Carbon atoms of hydrophobic residues are highlighted in green and carbon atoms of polar residues are highlighted in light blue. TAIII is represented by sticks with the carbon atoms in yellow. Colour code: carbon (yellow, green or light blue), nitrogen (dark blue), oxygen (red), and hydrogen (white).

Taken together, our data demonstrate that timosaponins modulate APP processing with a suppression of β-cleavage and selective reduction in Aβ_42_ production. To establish a possible binding model of the molecular targeting of Aβ, molecular simulation of timosaponins to APP was performed. We postulate that timosaponin binds to APP because there is evidence for interactions between a number of GSM and APP, particularly at its transmembrane region that contains the sites of γ-cleavage (Fig. 8A).^35^ We employed a model of an APP fragment (Protein Data Bank ID: ; 2LP1) that spans the extracellular juxtamembrane and transmembrane domains (TMD) (Fig. 8). Previous NMR analyses indicate that the extracellular amino terminus includes a surface-embedded “N-helix” followed by a short “N-loop” connecting to the TMD.^20^ Importantly, a binding pocket for cholesterol, centred around the N-helix/N-loop/TMD structural element, has been identified (Fig. 8A).^20^ Our preliminary molecular docking analysis revealed that SSG and timosaponins can be positioned within the cholesterol binding pocket (ESI Fig. S14†). High level hybrid quantum mechanics/molecular mechanics (QM/MM) calculation (ESI†) was performed to provide in depth understanding on the timosaponin binding to the transmembrane domain of APP (Fig. 8B and C). The results imply that the binding interaction is selective, which is probably due to the specific polarity of the SSG-aglyconed timosaponins (Fig. 8B and C). The parent SSG part of TAIII containing the lipophilic steroid ring is surrounded by a group of hydrophobic residues (689–692, 695, 696, 704, 705, 707–712), while the polar part of TAIII possessing the hydrophilic galactose moiety is surrounded by several polar residues (697–699) and lies at the surface of the transmembrane. In particular, the binding pose of the SSG motif is proximal to the GXXXG motifs (particularly, the G_700_AIIG_704_) of the TMD. These motifs have been shown to be important in the production of the long and short forms of the Aβ polypeptides mediated by γ-secretase. In addition, these motifs are involved in the non-steroidal anti-inflammatory drug (NSAID)-derived GSM modulation of γ-secretase.^36^,^37^ Nonetheless, further experiments (*e.g.*, crystallography) are needed to validate the specific binding mode. It is noteworthy that the modulation of APP processing by some endogenous steroid-like compounds from animals and plants has been recently reported.^38^,^39^

#### *In vivo* Aβ-lowering activities of timosaponins

**Fig. 9:**
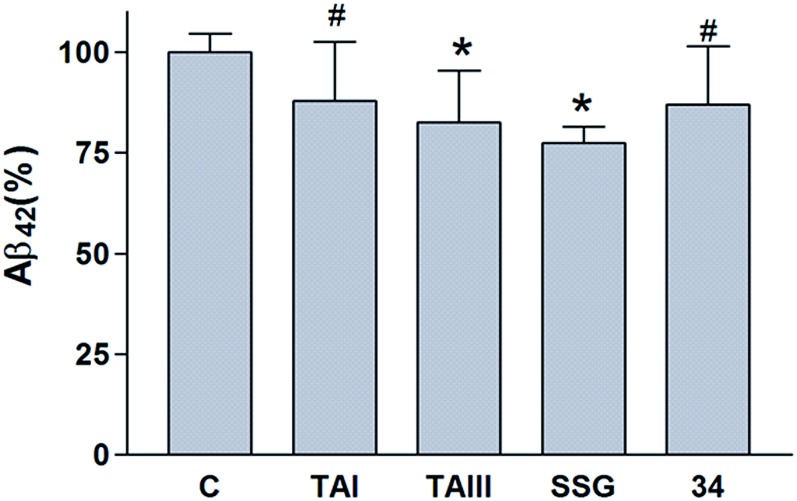
*In vivo* Aβ_42_-lowering activities of SSG and timosaponins. 3–5 month old C57BL/6 mice were dosed with vehicle (C), TAI, TAIII, SSG or α-, β-OH SSG (**34**) at 100 mg kg^–1^ by oral gavage for three times in 2 days. The levels of Aβ_42_ in the mouse brain were determined by ELISA. The number (*n*) of animals for vehicle control, TAI, TAIII and SSG = 10; for **34**, *n* = 6. Data represent means ± standard deviations. Statistical significance in differences between vehicle control and treatment groups was determined by Student's test, #, *P* < 0.1; *, *P* < 0.05.

The *in vivo* Aβ-lowering activities of timosaponins were examined in mice. A group of 3–5 month old C57BL/6 mice was dosed with SSG (**1**), TAIII (**2**), TAI (**3**) or α-, β-OH SSG (**34**) at 100 mg kg^–1^ by oral gavage for three times in 2 days. The results showed that these compounds elicited a reduction of Aβ_42_ levels in the brain (77% ± 4% for SSG, *P* < 0.05; 83% ± 13% for TAIII, *P* < 0.05; 87% ± 14% for TAI, *P* = 0.09; 87% ± 15% for **34**; *P* = 0.09) (Fig. 9). Such a moderate degree of Aβ_42_ reduction has been demonstrated by many Aβ-lowering natural compounds (ESI Table S1†). We also determined the levels of SSG **1**, timosaponins **2–3**, compound **34** and their metabolites in the plasma and brain of the mice by ultra-performance liquid chromatography tandem mass spectrometry. The timosaponins and/or their deglycosylated products (TAI and SSG) at low micromolar concentrations could be detected in the plasma and brain at the end of the experiments (ESI Table S2†). No further metabolites except the deglycosylated products (TAI, SSG from TAIII; SSG from TAI) were detected. The reason for the scarcity of TAIII in the brain is uncertain but may be attributed to a lower ability of the glycosylated compounds to cross the blood brain barrier and/or an elevated glycohydrolase activity in the neurons.^40^ Compound **34** was present at a much lower level in the mouse plasma and brain compared to its closest analogue TAI, suggesting that the nature of C_3_ linkage may have an impact on the bioavailability and/or tissue distribution. Further pharmacokinetics studies of SSG and the timosaponins and their derivatives are required to elucidate the structural features of the timosaponins required for optimal bioavailability and efficacy. Collectively, our results reveal that SSG and certain timosaponins display oral bioavailability, brain penetration capacity and Aβ-lowering activity *in vivo*.

## Conclusion

The timosaponins investigated in the current study are preferentially able to lower Aβ_42_ production and stimulate neurite outgrowth, largely due to the presence of the effective aglycone SSG **1**. They contain structural features including a *cis*-fused AB ring, 3β-configuration and an intact F-spiroketal ring with a 25*S*-configuration. These characteristics are indispensable structural requirements for the compound's dual properties. The Aβ-lowering activities of “SSG-aglyconed” timosaponins are generally associated with decreases in β-cleavage and/or increases in α-cleavage of APP. These are accompanied by a preferential reduction of Aβ_42_ levels without affecting the processing of other γ-secretase substrates, resembling the action of GSM. Thus, the “SSG-aglyconed” timosaponins are novel agents that modulate APP processing and subsequently lower Aβ production. We have also shown here that some timosaponins and SSG exhibit Aβ-lowering activities *in vivo*. It is envisaged that, when properly modified and formulated, timosaponins will be intriguing compounds for the development of AD therapeutics.

## Supplementary Material

Supplementary information

Supplementary information
