# Supplementary material for: Identification of “sarsasapogenin-aglyconed” timosaponins as novel Aβ-lowering modulators of amyloid precursor protein processing
Source: Chem Sci. 2016 Jan 22;7(5):3206–14. doi: 10.1039/c5sc02377g (PMC6005341; doi:10.1039/c5sc02377g)

Electronic Supplementary Material (ESI) for Chemical Science.
This journal is © The Royal Society of Chemistry 2016

Appendix I

^{1}H NMR of Timosaponin A III (**2**)

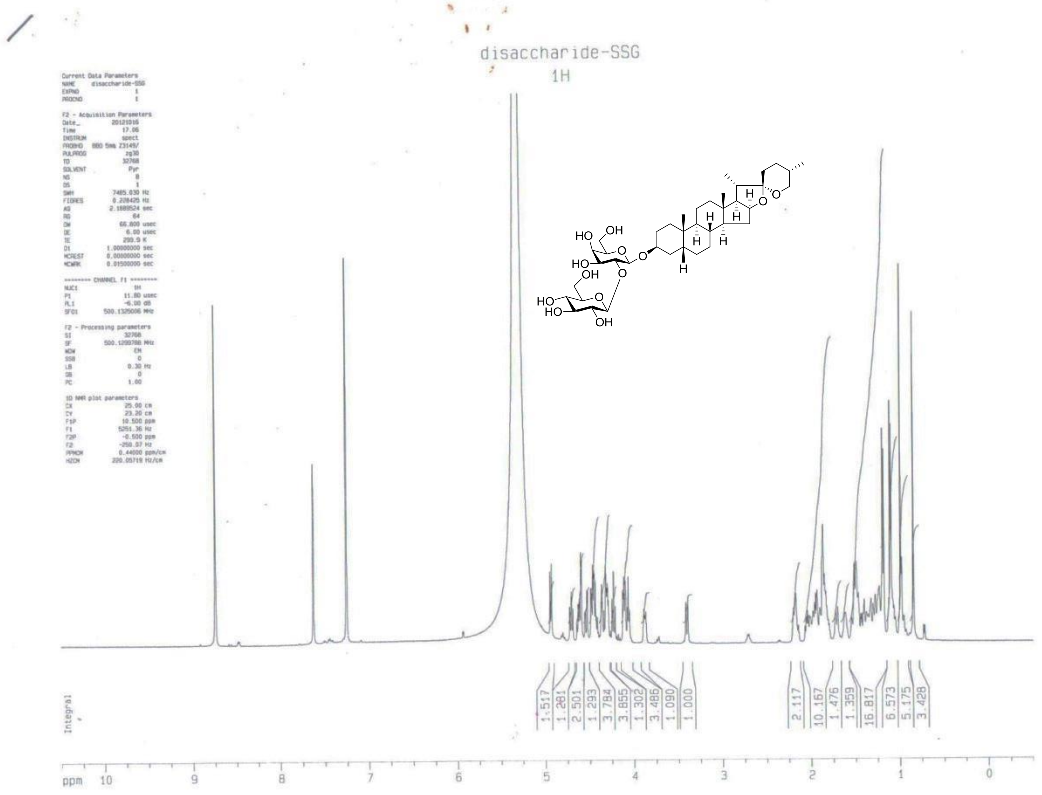

^{13}C NMR of Timosaponin A III (**2**)

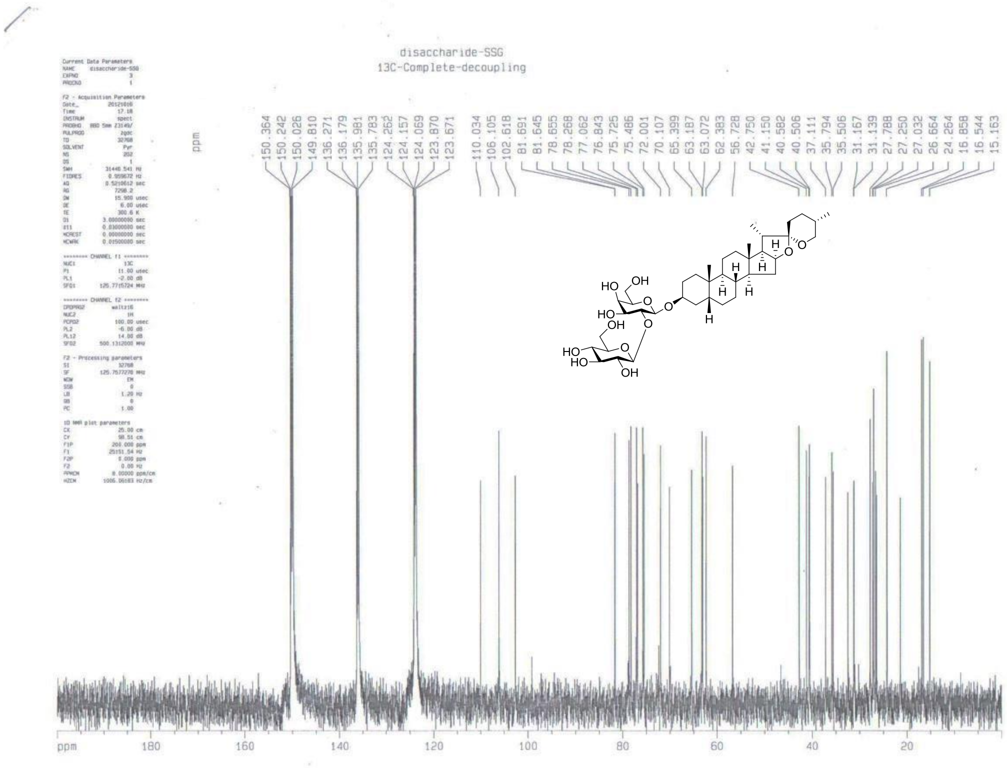

^{1}H NMR of Timosaponin A I (**3**)

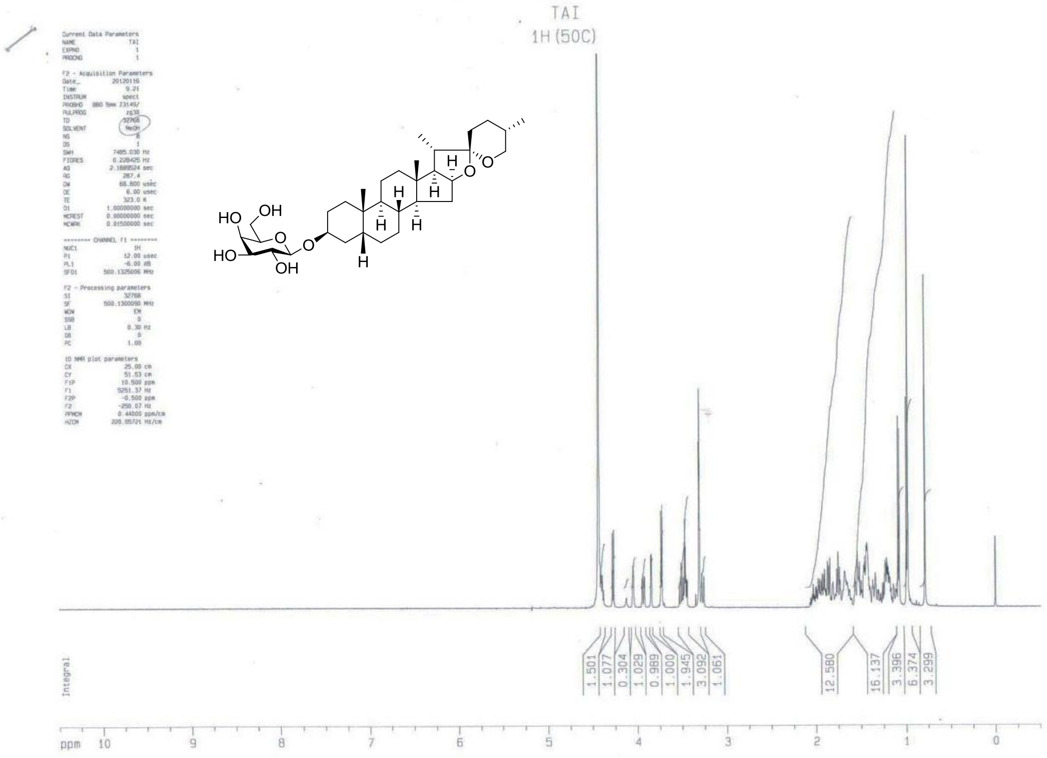

^{13}C NMR of Timosaponin A I (**3**)

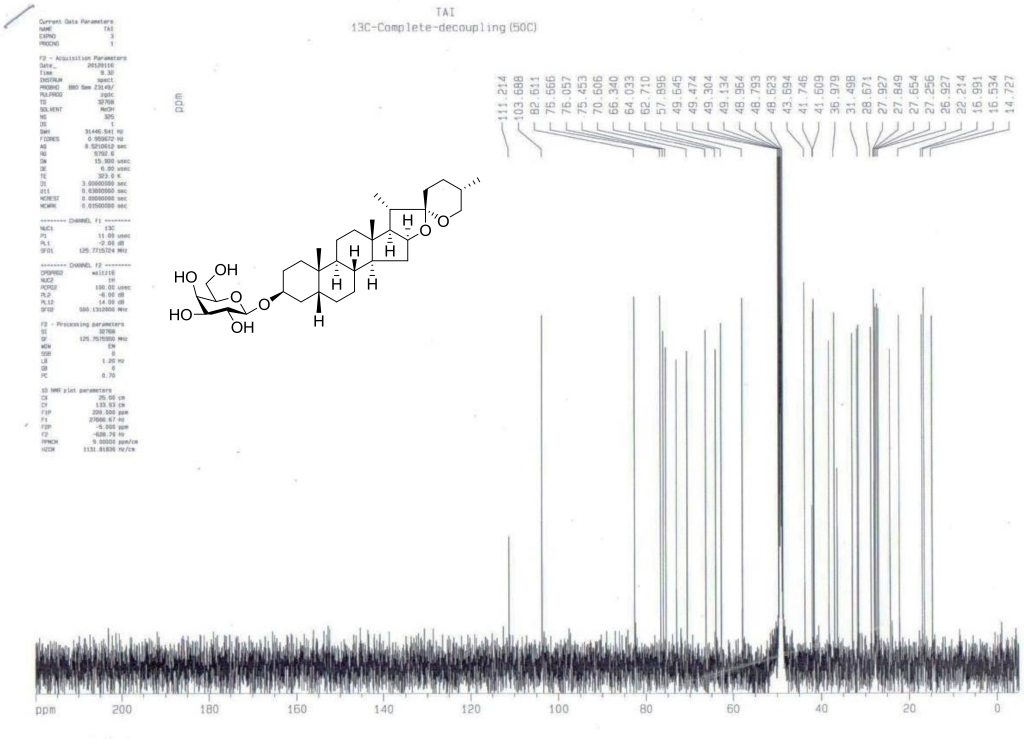

Dept-135 of Timosaponin A I (**3**)

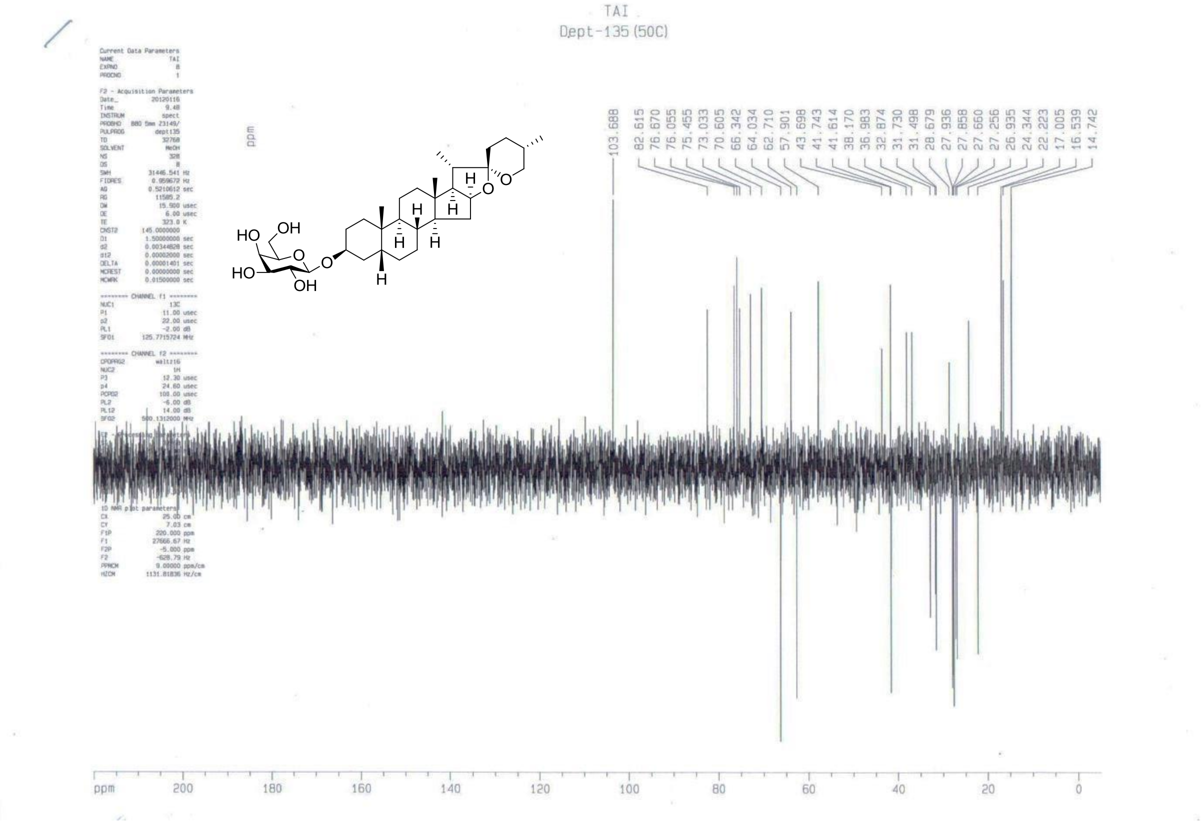

^{1}H NMR of Timosaponin A V (**4**)

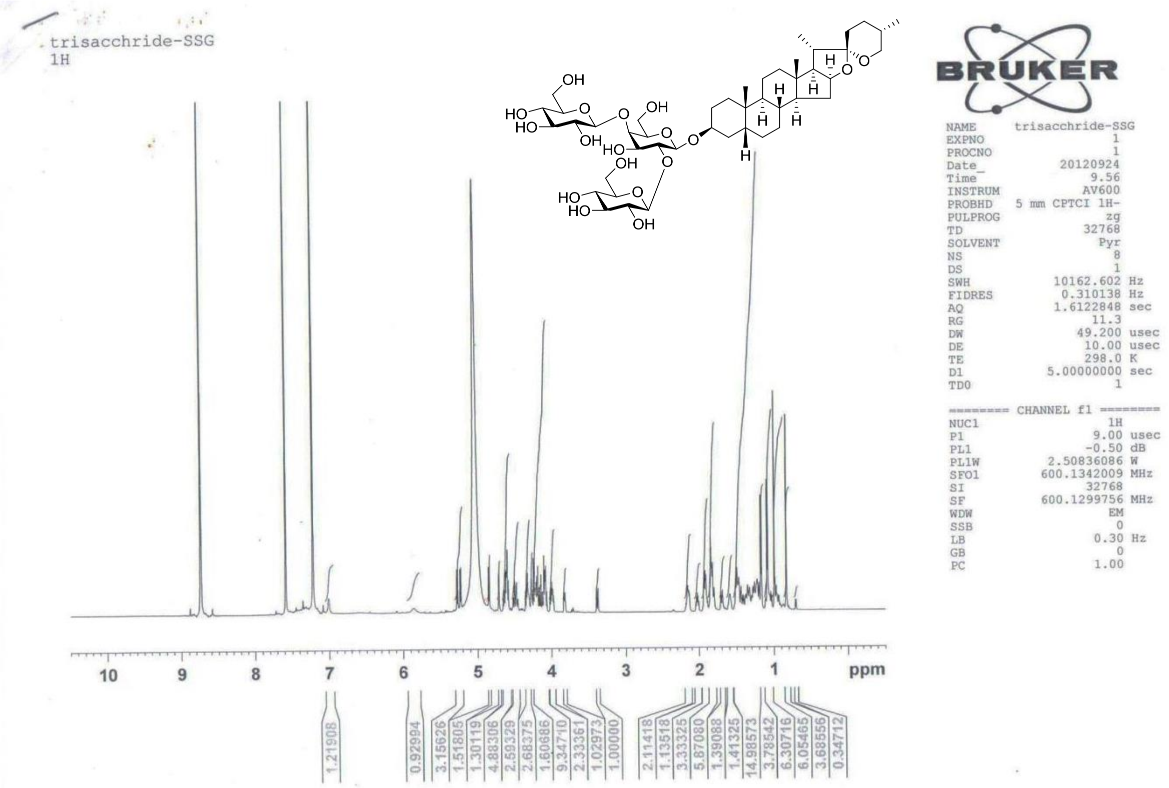

^{13}C NMR of Timosaponin A V (**4**)

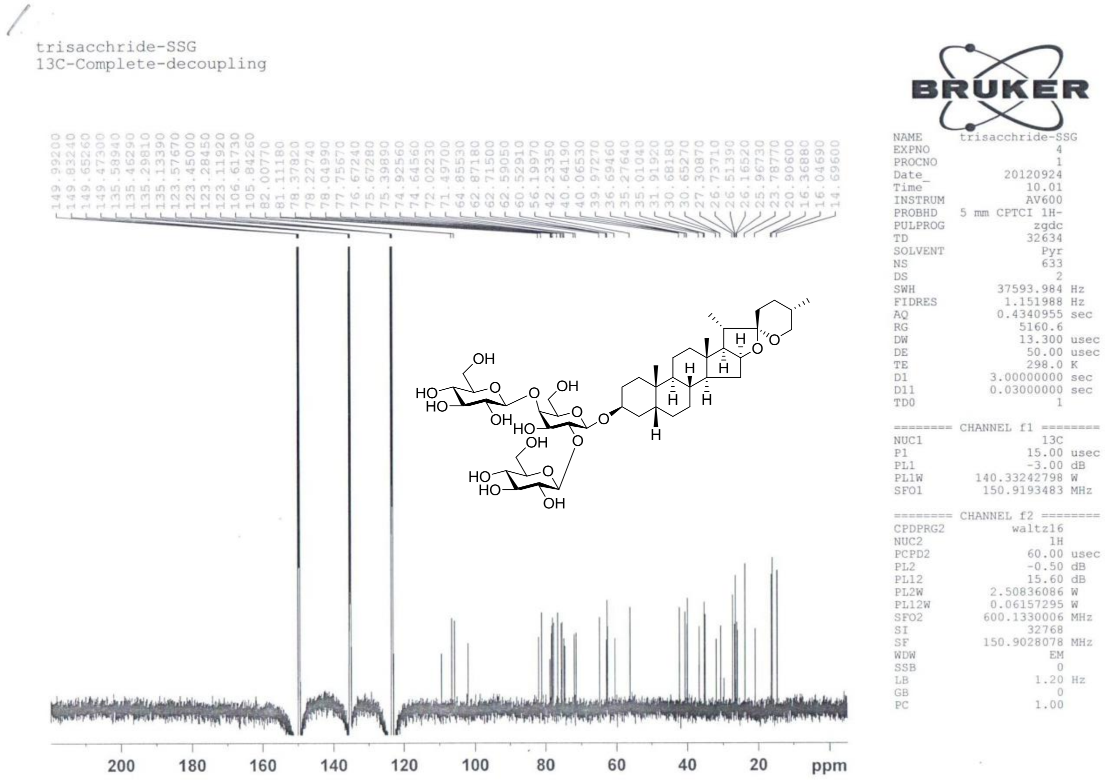

Dept-135 of Timosaponin A V (**4**)

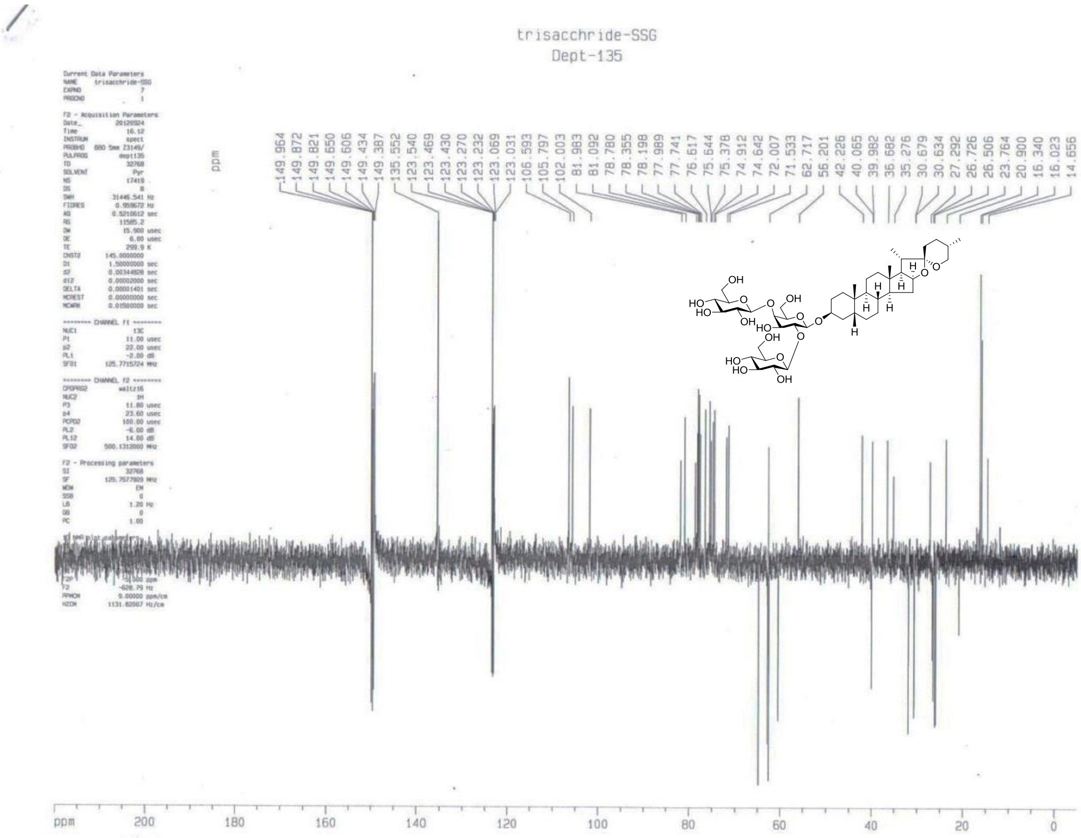

HSQC of Timosaponin A V (**4**)

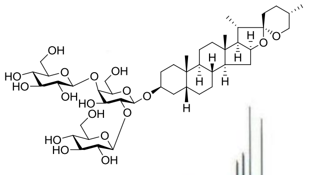

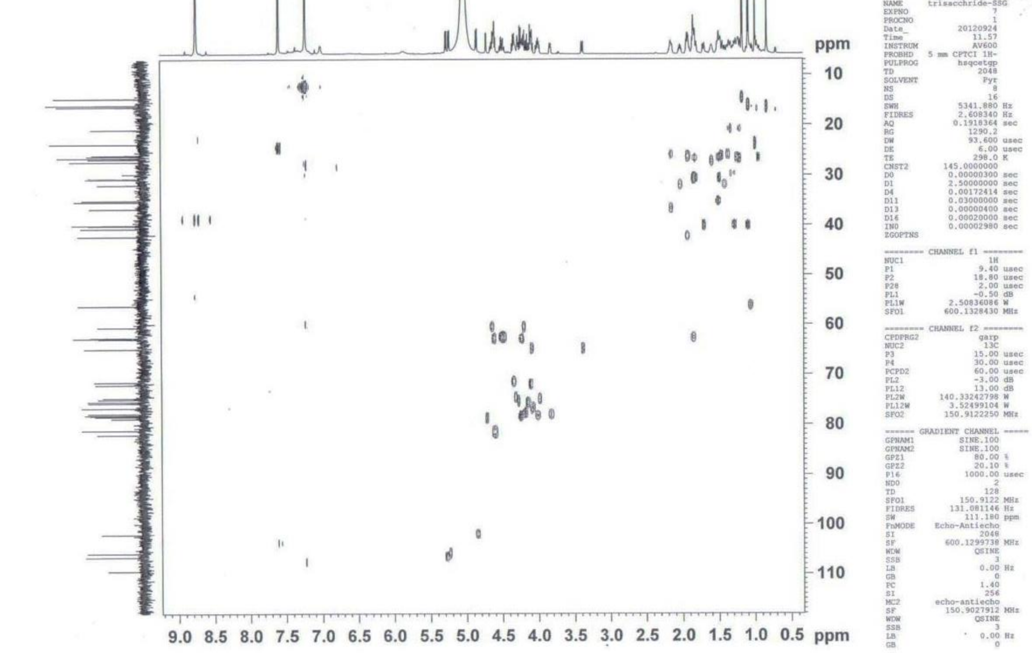

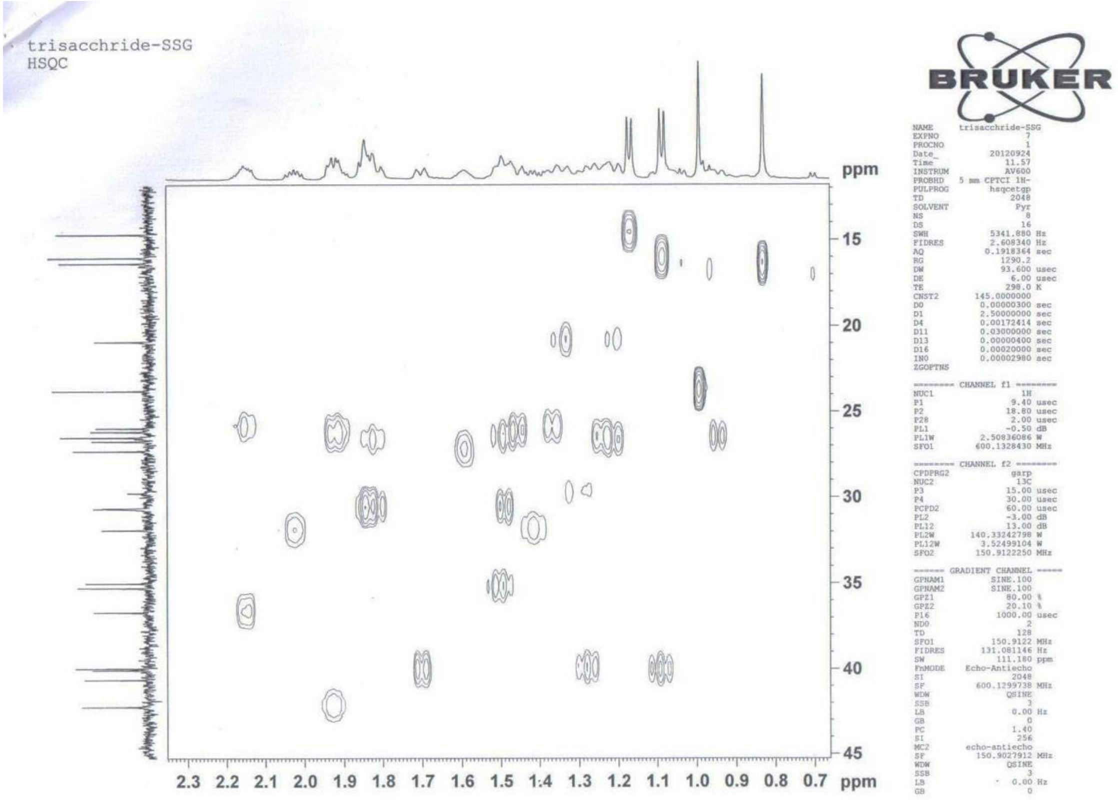

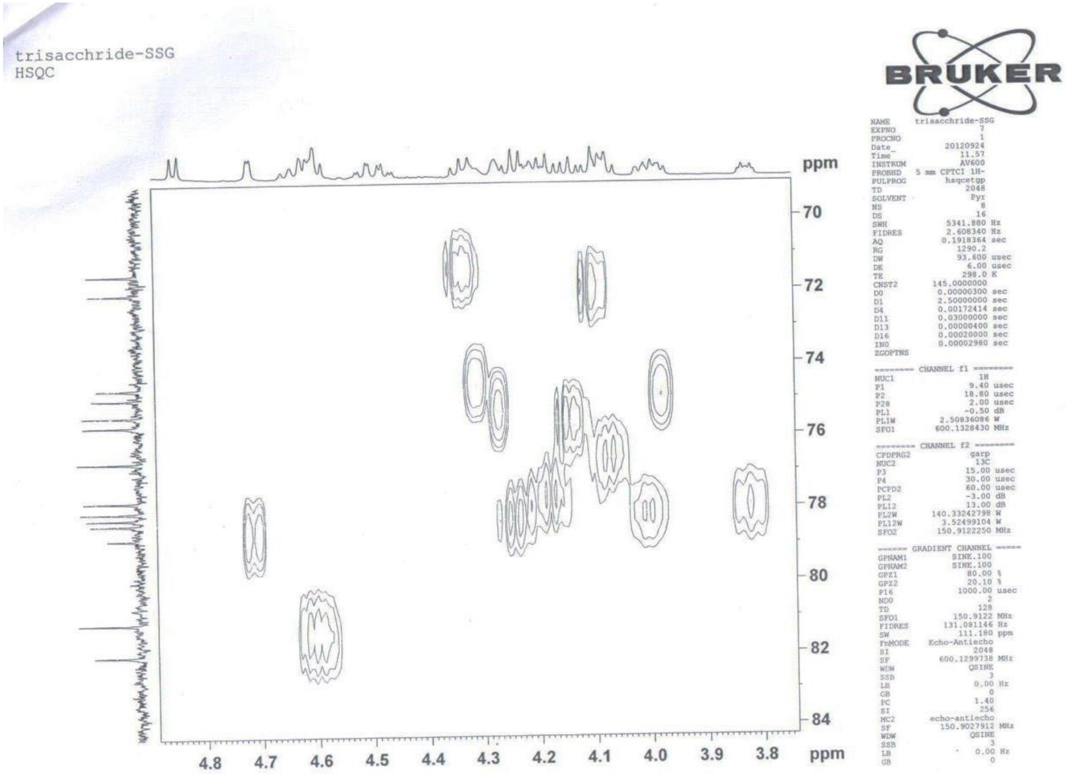

HH-COSY of Timosaponin A V (**4**)

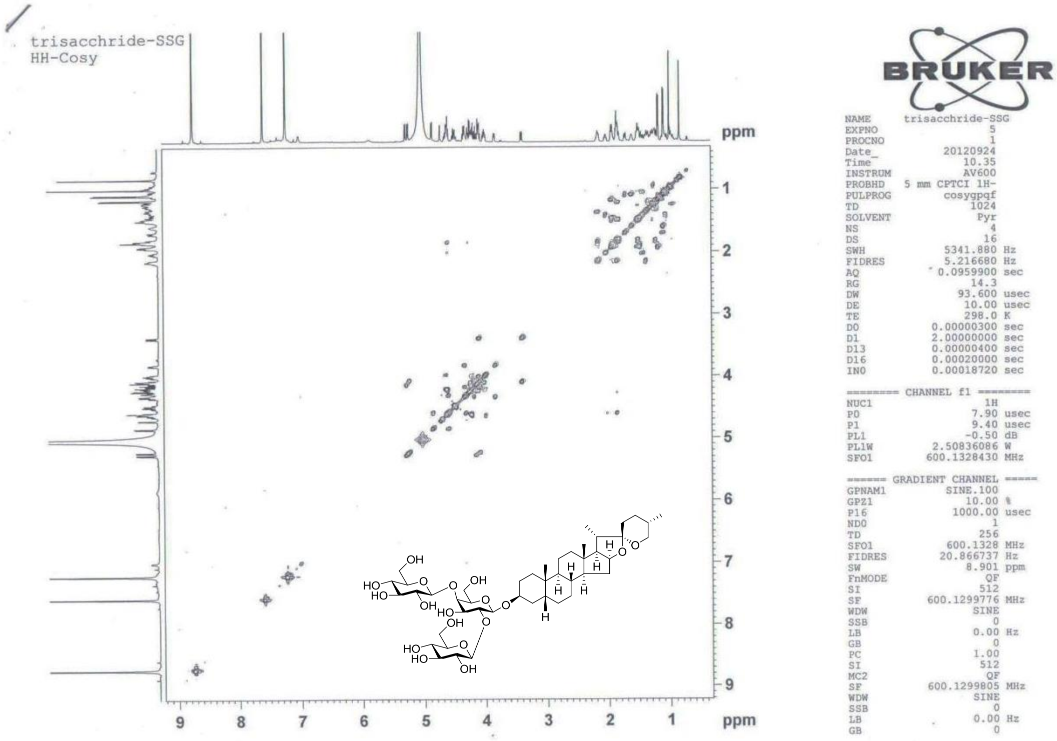

trisacchride-SSG
HH-Cosy

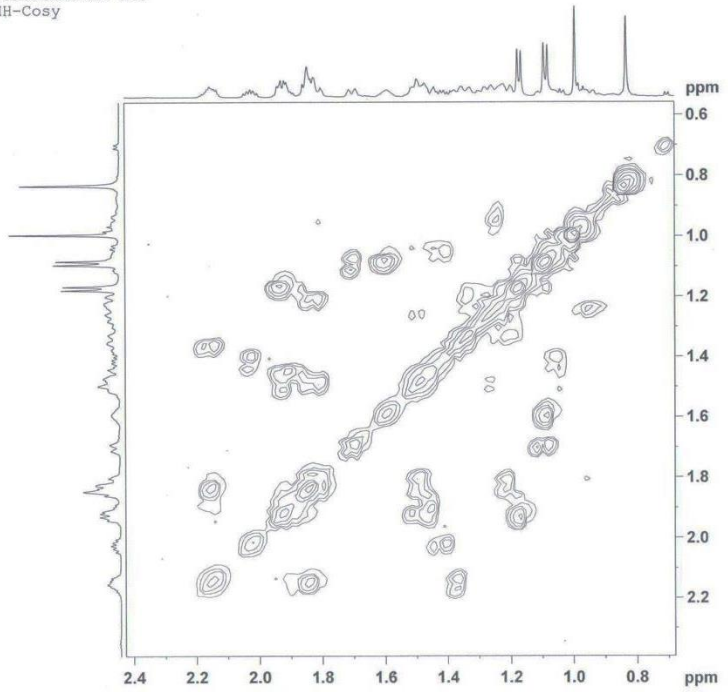

BRUKER

```
NAME       trisacchride-SSG
EXPNO                     5
PROCNO                    1
Date_              20120924
Time                  10.35
INSTRUM               AV600
PROBHD   5 mm CPTCI 1H-
PULPROG           cosygpqf
TD                     1024
SOLVENT                 Pyr
NS                        4
DS                       16
SWH            5341.880 Hz
FIDRES         5.216680 Hz
AQ            0.0959900 sec
RG                     14.3
DW               93.600 usec
DE                10.00 usec
TE                298.0 K
D0           0.00000300 sec
D1           2.00000000 sec
D13          0.00000400 sec
D16          0.00020000 sec
IN0          0.00018720 sec

======== CHANNEL f1 ========
NUC1                     1H
P0                 7.90 usec
P1                 9.40 usec
PL1               -0.50 dB
PL1W         2.50836086 W
SFO1       600.1328430 MHz

====== GRADIENT CHANNEL =====
GPNAM1             SINE.100
GPZ1               10.00 %
P16              1000.00 usec
ND0                       1
TD                      256
SFO1           600.1328 MHz
FIDRES        20.866737 Hz
SW                8.901 ppm
FnMODE                   QF
SI                      512
SF          600.1299776 MHz
WDW                    SINE
SSB                       0
LB                 0.00 Hz
GB                        0
PC                     1.00
SI                      512
MC2                      QF
SF          600.1299805 MHz
WDW                    SINE
SSB                       0
LB                 0.00 Hz
GB                        0
```

trisacchride-SSG
HH-Cosy

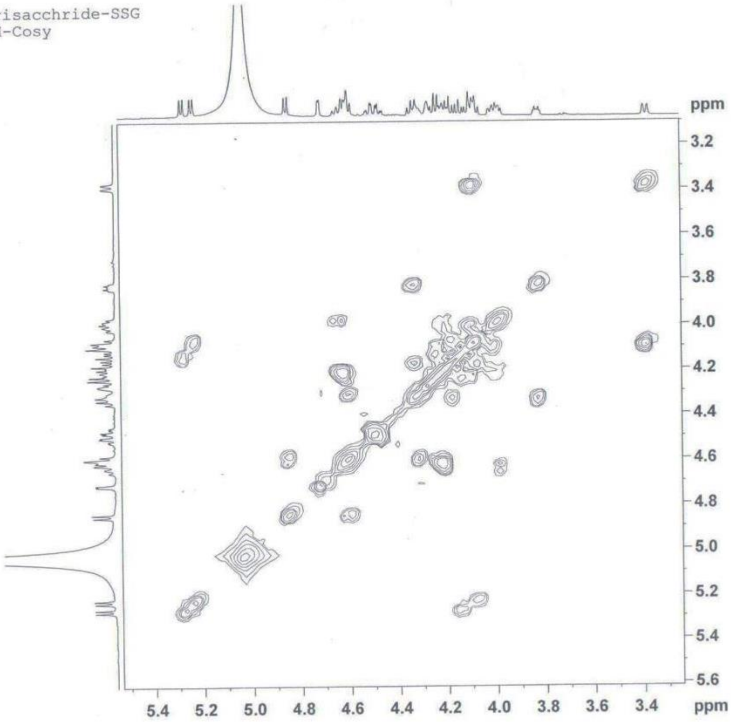

BRUKER

```
NAME       trisacchride-SSG
EXPNO                     5
PROCNO                    1
Date_              20120924
Time                  10.35
INSTRUM               AV600
PROBHD   5 mm CPTCI 1H-
PULPROG           cosygpqf
TD                     1024
SOLVENT                 Pyr
NS                        4
DS                       16
SWH            5341.880 Hz
FIDRES         5.216680 Hz
AQ            0.0959900 sec
RG                     14.3
DW               93.600 usec
DE                10.00 usec
TE                298.0 K
D0           0.00000300 sec
D1           2.00000000 sec
D13          0.00000400 sec
D16          0.00020000 sec
IN0          0.00018720 sec

======== CHANNEL f1 ========
NUC1                     1H
P0                 7.90 usec
P1                 9.40 usec
PL1               -0.50 dB
PL1W         2.50836086 W
SFO1       600.1328430 MHz

====== GRADIENT CHANNEL =====
GPNAM1             SINE.100
GPZ1               10.00 %
P16              1000.00 usec
ND0                       1
TD                      256
SFO1           600.1328 MHz
FIDRES        20.866737 Hz
SW                8.901 ppm
FnMODE                   QF
SI                      512
SF          600.1299776 MHz
WDW                    SINE
SSB                       0
LB                 0.00 Hz
GB                        0
PC                     1.00
SI                      512
MC2                      QF
SF          600.1299805 MHz
WDW                    SINE
SSB                       0
LB                 0.00 Hz
GB                        0
```

HMBC of Timosaponin A V (**4**)

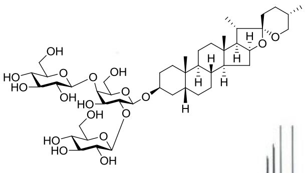

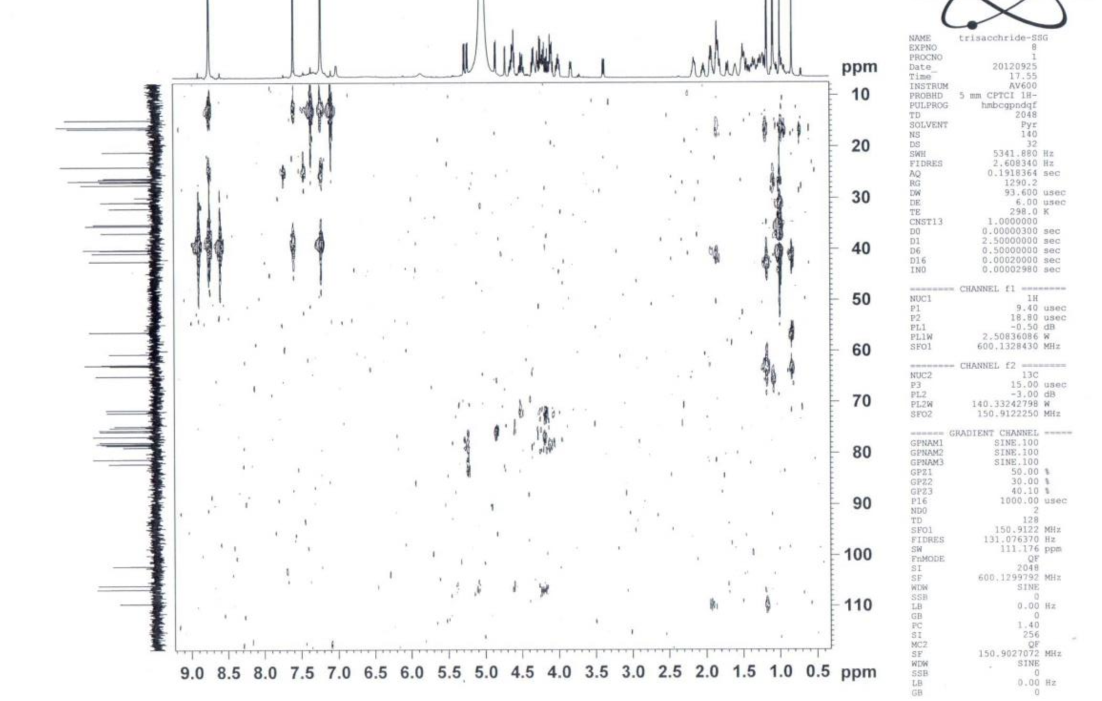

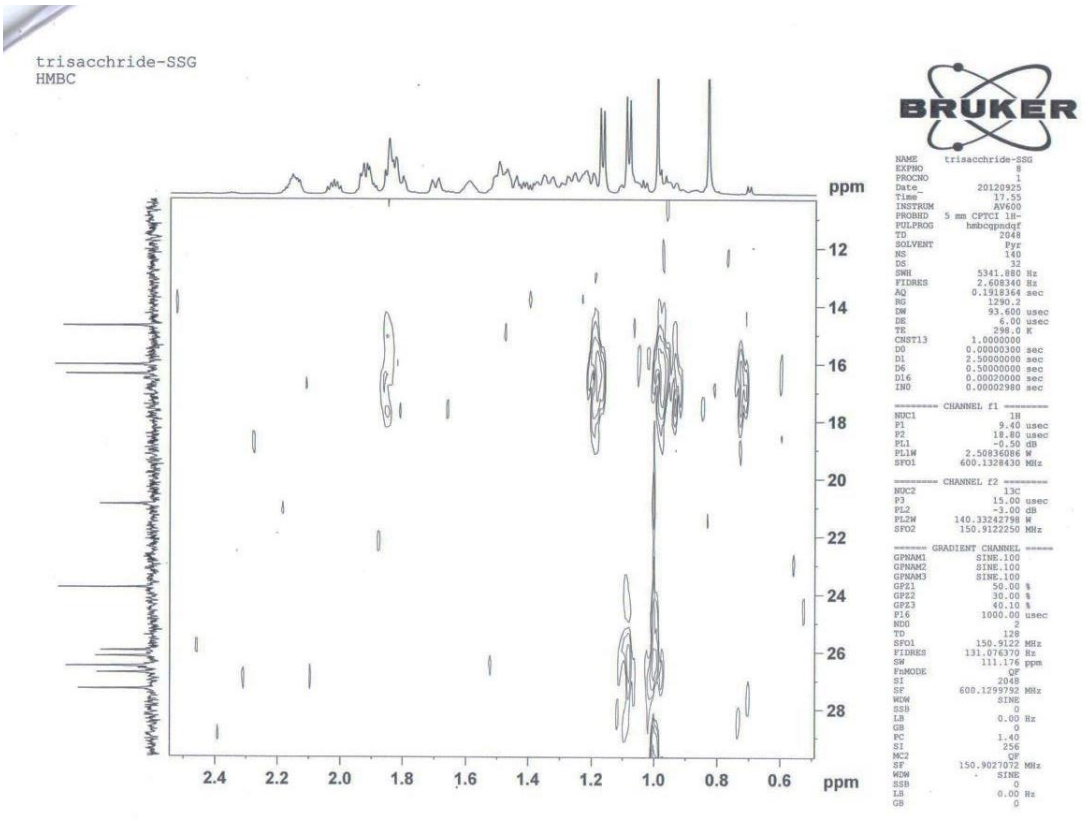

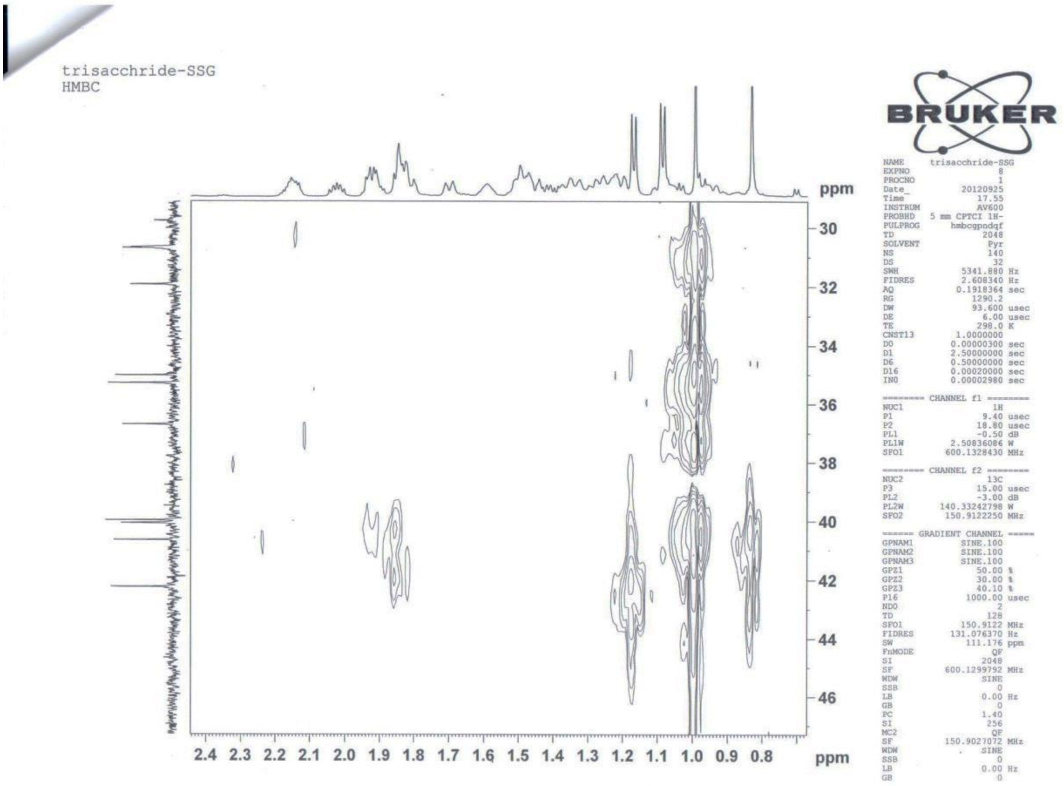
trisacchride-SSG
HMBC
ppm
30
32
34
36
38
40
42
44
46
2.4 2.3 2.2 2.1 2.0 1.9 1.8 1.7 1.6 1.5 1.4 1.3 1.2 1.1 1.0 0.9 0.8
ppm
NAME trisacchride-SSG
EXPNO 8
PROCNO 1
Date_ 20120925
Time 17.55
INSTRUM AV600
PROBHD 5 mm CPTCI 1H-
PULPROG hmbcgpndqf
TD 2048
SOLVENT Pyr
NS 140
DS 32
SWH 5341.880 Hz
FIDRES 2.608340 Hz
AQ 0.1918364 sec
RG 1290.2
DW 93.600 usec
DE 6.00 usec
TE 298.0 K
CNST13 1.0000000
D0 0.00000300 sec
D1 2.50000000 sec
D6 0.50000000 sec
D16 0.00020000 sec
IN0 0.00002980 sec
======== CHANNEL f1 ========
NUC1 1H
P1 9.40 usec
P2 18.80 usec
PL1 -0.50 dB
PL1W 2.50836086 W
SFO1 600.1328430 MHz
======== CHANNEL f2 ========
NUC2 13C
P3 15.00 usec
PL2 -3.00 dB
PL2W 140.33242798 W
SFO2 150.9122250 MHz
====== GRADIENT CHANNEL =====
GPNAM1 SINE.100
GPNAM2 SINE.100
GPNAM3 SINE.100
GPZ1 50.00 %
GPZ2 30.00 %
GPZ3 40.10 %
P16 1000.00 usec
ND0 2
TD 128
SFO1 150.9122 MHz
FIDRES 131.076370 Hz
SW 111.176 ppm
FnMODE QF
SI 2048
SF 600.1299792 MHz
WDW SINE
SSB 0
LB 0.00 Hz
GB 0
PC 1.40
SI 256
MC2 QF
SF 150.9027072 MHz
WDW SINE
SSB 0
LB 0.00 Hz
GB 0

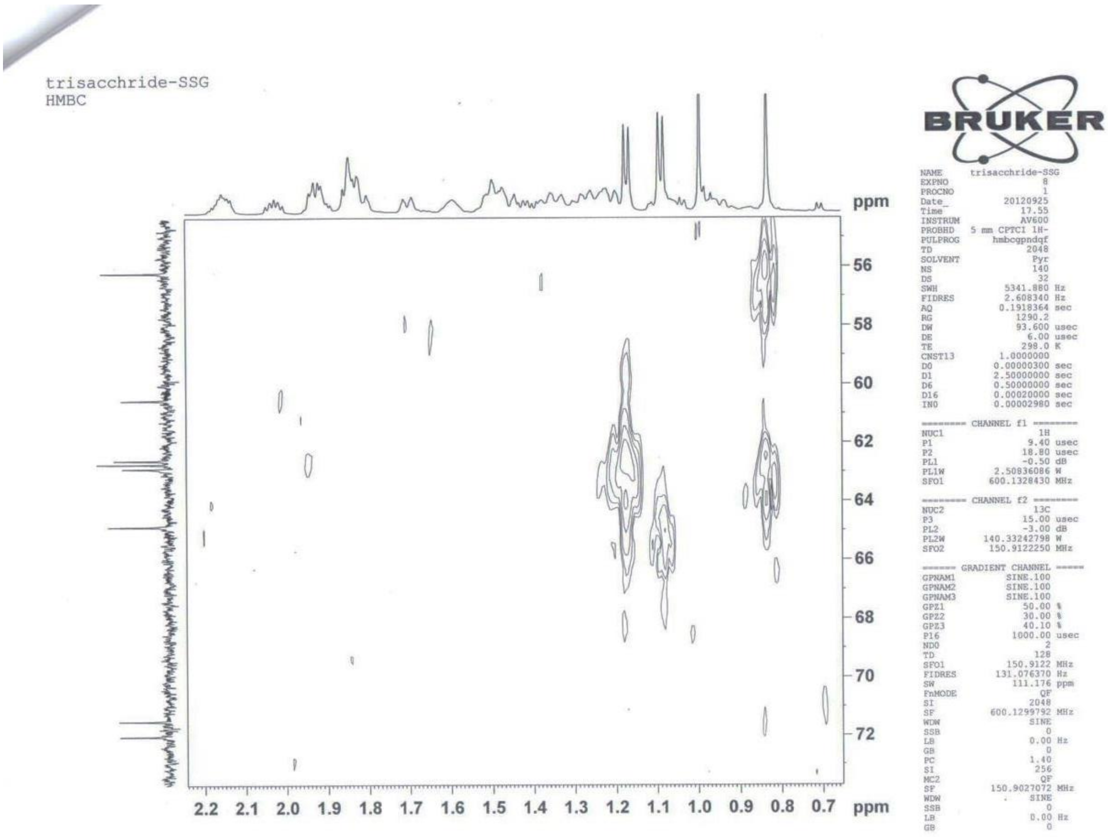
trisacchride-SSG
HMBC
ppm
56
58
60
62
64
66
68
70
72
2.2 2.1 2.0 1.9 1.8 1.7 1.6 1.5 1.4 1.3 1.2 1.1 1.0 0.9 0.8 0.7
ppm
NAME trisacchride-SSG
EXPNO 8
PROCNO 1
Date_ 20120925
Time 17.55
INSTRUM AV600
PROBHD 5 mm CPTCI 1H-
PULPROG hmbcgpndqf
TD 2048
SOLVENT Pyr
NS 140
DS 32
SWH 5341.880 Hz
FIDRES 2.608340 Hz
AQ 0.1918364 sec
RG 1290.2
DW 93.600 usec
DE 6.00 usec
TE 298.0 K
CNST13 1.0000000
D0 0.00000300 sec
D1 2.50000000 sec
D6 0.50000000 sec
D16 0.00020000 sec
IN0 0.00002980 sec
======== CHANNEL f1 ========
NUC1 1H
P1 9.40 usec
P2 18.80 usec
PL1 -0.50 dB
PL1W 2.50836086 W
SFO1 600.1328430 MHz
======== CHANNEL f2 ========
NUC2 13C
P3 15.00 usec
PL2 -3.00 dB
PL2W 140.33242798 W
SFO2 150.9122250 MHz
====== GRADIENT CHANNEL =====
GPNAM1 SINE.100
GPNAM2 SINE.100
GPNAM3 SINE.100
GPZ1 50.00 %
GPZ2 30.00 %
GPZ3 40.10 %
P16 1000.00 usec
ND0 2
TD 128
SFO1 150.9122 MHz
FIDRES 131.076370 Hz
SW 111.176 ppm
FnMODE QF
SI 2048
SF 600.1299792 MHz
WDW SINE
SSB 0
LB 0.00 Hz
GB 0
PC 1.40
SI 256
MC2 QF
SF 150.9027072 MHz
WDW SINE
SSB 0
LB 0.00 Hz
GB 0

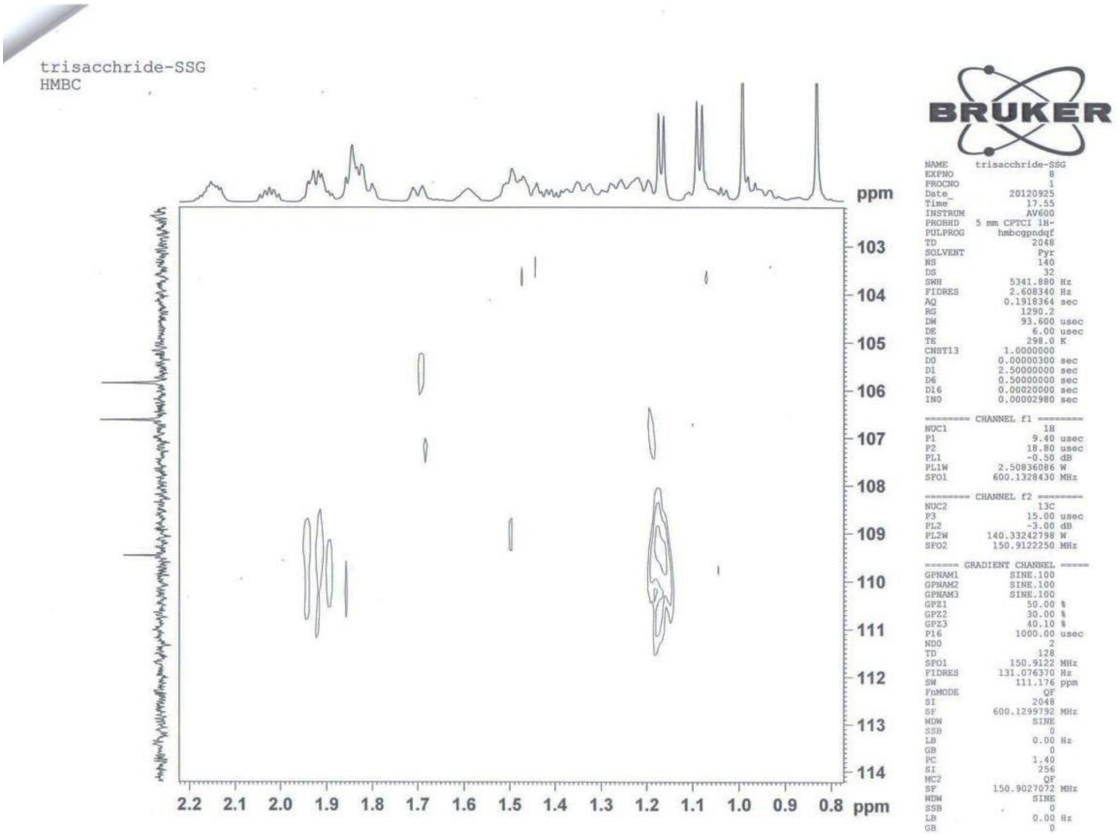
trisacchride-SSG
HMBC
ppm
103
104
105
106
107
108
109
110
111
112
113
114
2.2 2.1 2.0 1.9 1.8 1.7 1.6 1.5 1.4 1.3 1.2 1.1 1.0 0.9 0.8 ppm
NAME trisacchride-SSG
EXPNO 8
PROCNO 1
Date_ 20120925
Time 17.55
INSTRUM AV600
PROBHD 5 mm CPTCI 1H-
PULPROG hmbcgpndqf
TD 2048
SOLVENT Pyr
NS 140
DS 32
SWH 5341.880 Hz
FIDRES 2.608340 Hz
AQ 0.1918364 sec
RG 1290.2
DW 93.600 usec
DE 6.00 usec
TE 298.0 K
CNST13 1.0000000
D0 0.00000300 sec
D1 2.50000000 sec
D6 0.50000000 sec
D16 0.00020000 sec
IN0 0.00002980 sec
======== CHANNEL f1 ========
NUC1 1H
P1 9.40 usec
P2 18.80 usec
PL1 -0.50 dB
PL1W 2.50836086 W
SFO1 600.1328430 MHz
======== CHANNEL f2 ========
NUC2 13C
P3 15.00 usec
PL2 -3.00 dB
PL2W 140.33242798 W
SFO2 150.9122250 MHz
====== GRADIENT CHANNEL =====
GPNAM1 SINE.100
GPNAM2 SINE.100
GPNAM3 SINE.100
GPZ1 50.00 %
GPZ2 30.00 %
GPZ3 40.10 %
P16 1000.00 usec
ND0 2
TD 128
SFO1 150.9122 MHz
FIDRES 131.076370 Hz
SW 111.176 ppm
FnMODE QF
SI 2048
SF 600.1299792 MHz
WDW SINE
SSB 0
LB 0.00 Hz
GB 0
PC 1.40
SI 256
MC2 QF
SF 150.9027072 MHz
WDW SINE
SSB 0
LB 0.00 Hz
GB 0

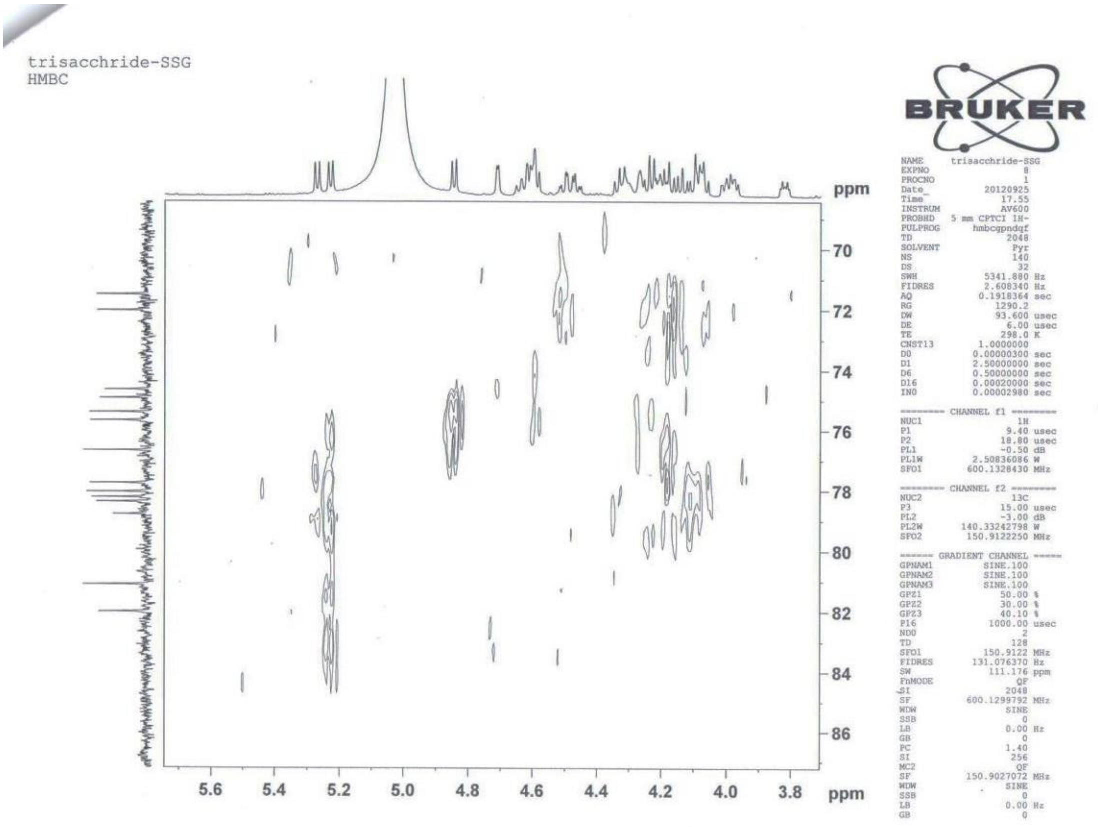
trisacchride-SSG
HMBC
ppm
70
72
74
76
78
80
82
84
86
5.6 5.4 5.2 5.0 4.8 4.6 4.4 4.2 4.0 3.8 ppm
NAME trisacchride-SSG
EXPNO 8
PROCNO 1
Date_ 20120925
Time 17.55
INSTRUM AV600
PROBHD 5 mm CPTCI 1H-
PULPROG hmbcgpndqf
TD 2048
SOLVENT Pyr
NS 140
DS 32
SWH 5341.880 Hz
FIDRES 2.608340 Hz
AQ 0.1918364 sec
RG 1290.2
DW 93.600 usec
DE 6.00 usec
TE 298.0 K
CNST13 1.0000000
D0 0.00000300 sec
D1 2.50000000 sec
D6 0.50000000 sec
D16 0.00020000 sec
IN0 0.00002980 sec
======== CHANNEL f1 ========
NUC1 1H
P1 9.40 usec
P2 18.80 usec
PL1 -0.50 dB
PL1W 2.50836086 W
SFO1 600.1328430 MHz
======== CHANNEL f2 ========
NUC2 13C
P3 15.00 usec
PL2 -3.00 dB
PL2W 140.33242798 W
SFO2 150.9122250 MHz
====== GRADIENT CHANNEL =====
GPNAM1 SINE.100
GPNAM2 SINE.100
GPNAM3 SINE.100
GPZ1 50.00 %
GPZ2 30.00 %
GPZ3 40.10 %
P16 1000.00 usec
ND0 2
TD 128
SFO1 150.9122 MHz
FIDRES 131.076370 Hz
SW 111.176 ppm
FnMODE QF
SI 2048
SF 600.1299792 MHz
WDW SINE
SSB 0
LB 0.00 Hz
GB 0
PC 1.40
SI 256
MC2 QF
SF 150.9027072 MHz
WDW SINE
SSB 0
LB 0.00 Hz
GB 0

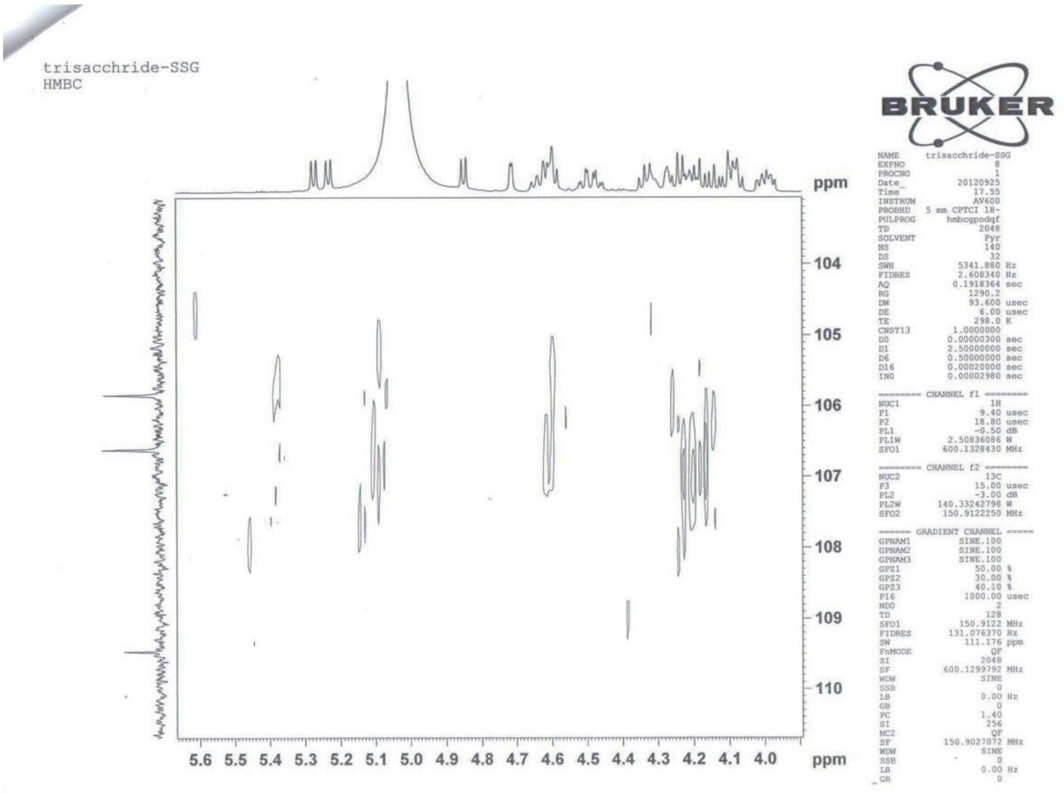

^{1}H NMR of Asparagoside A (**5**)

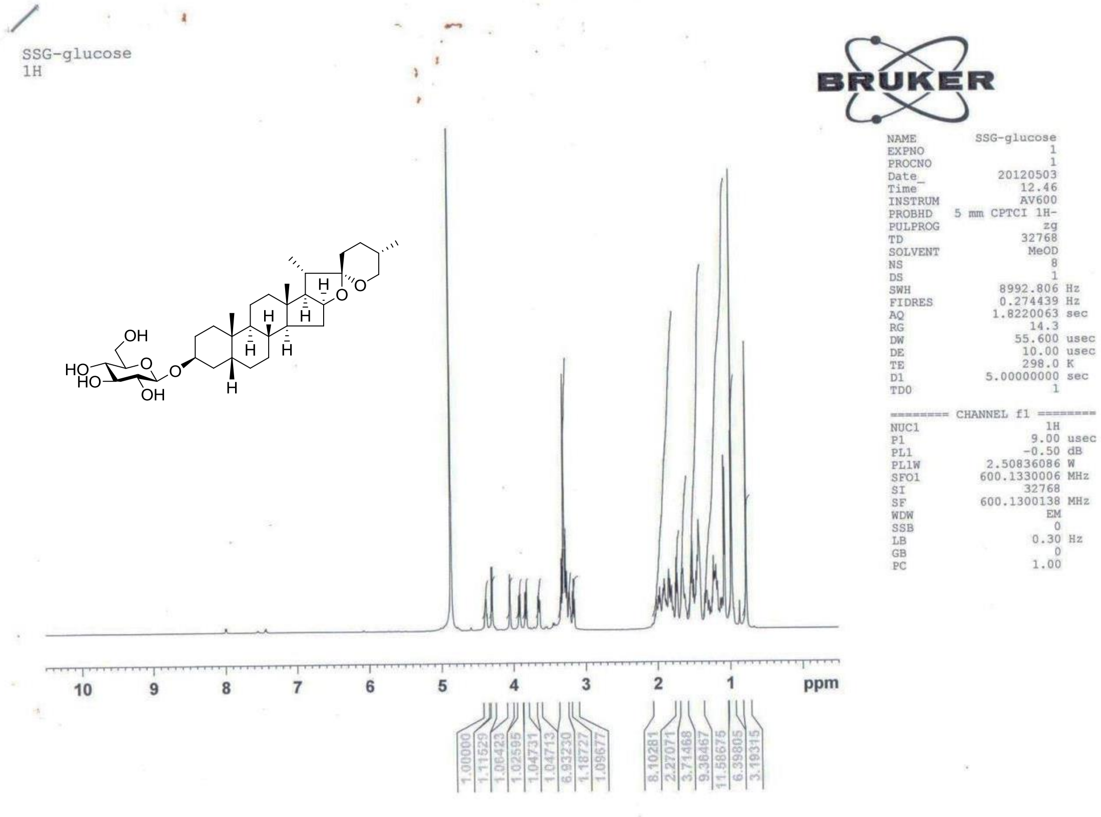

^{13}C NMR of Asparagoside A (**5**)

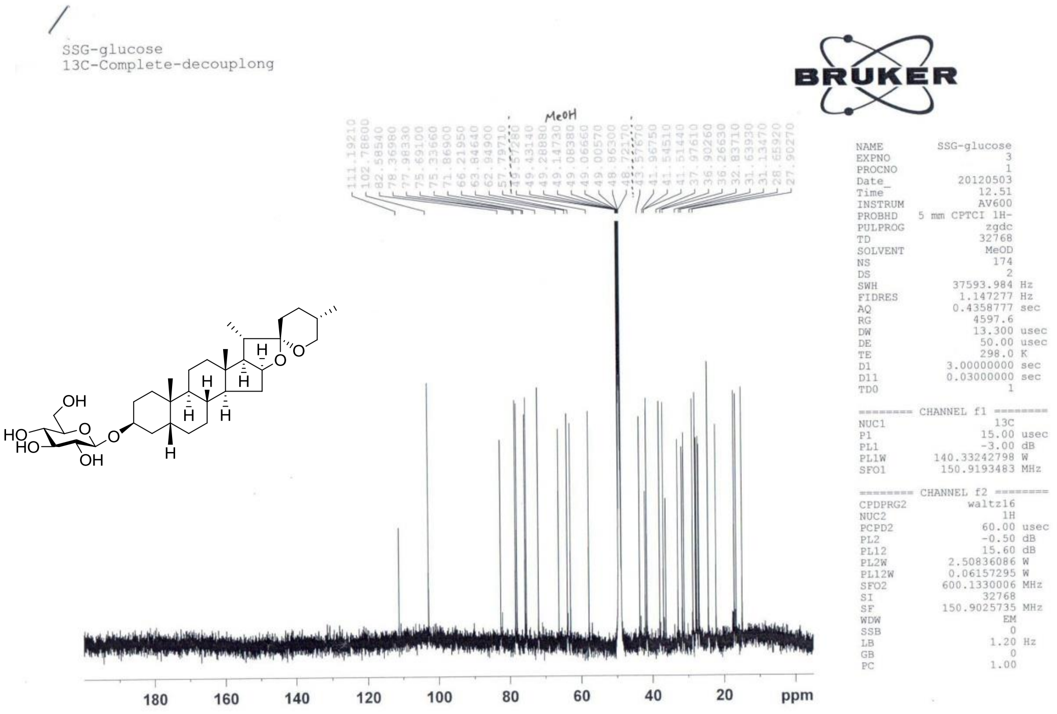

Dept-135 of Asparagoside A (**5**)

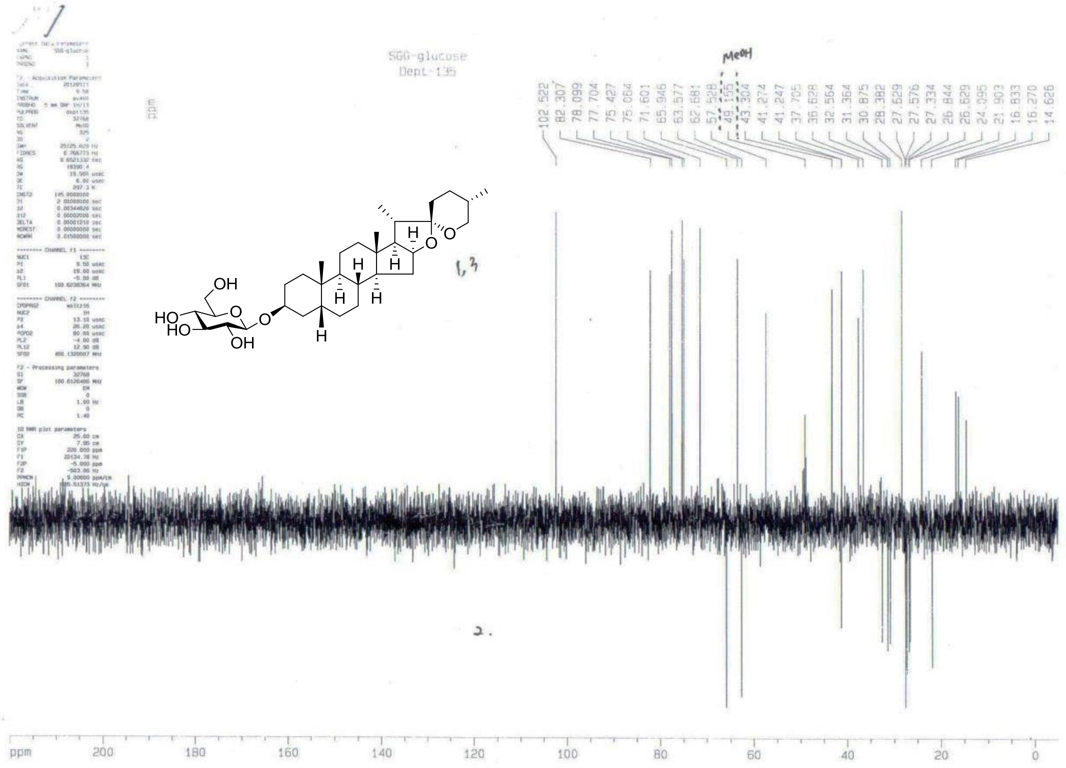

^{1}H NMR of Capsicoside A_3 (**8**)

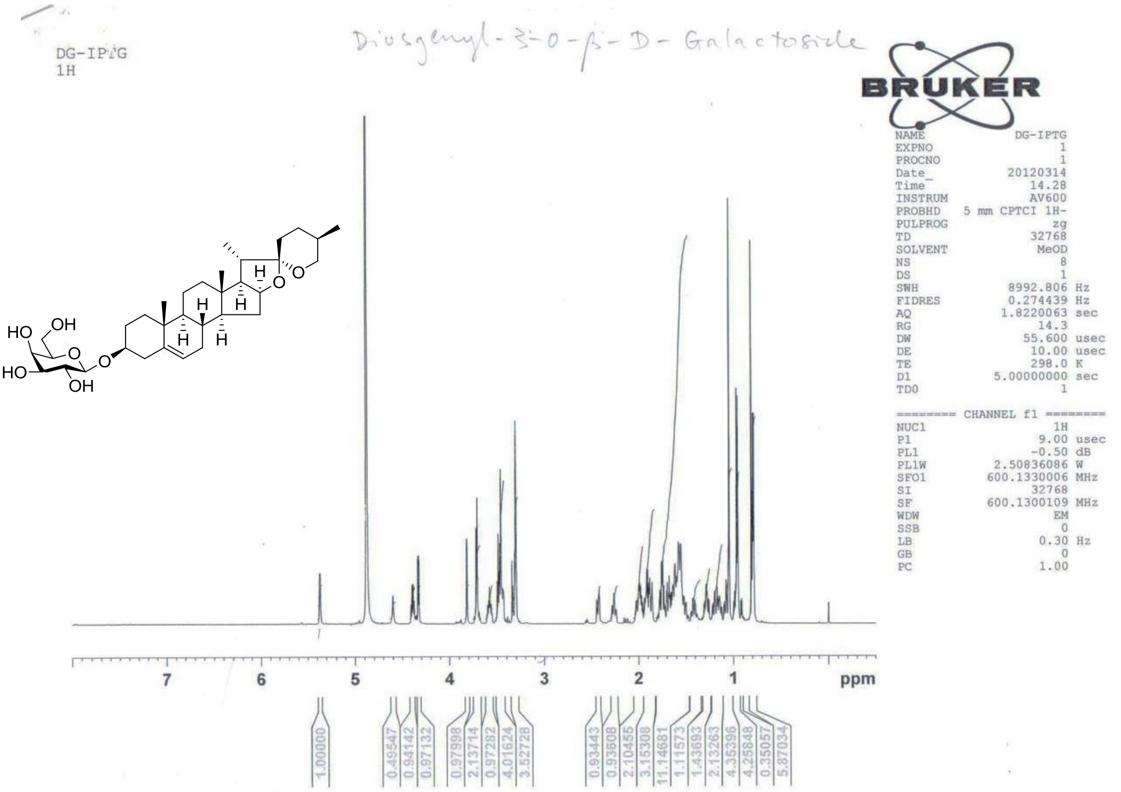

^{13}C NMR of Capsicoside A_3 (**8**)

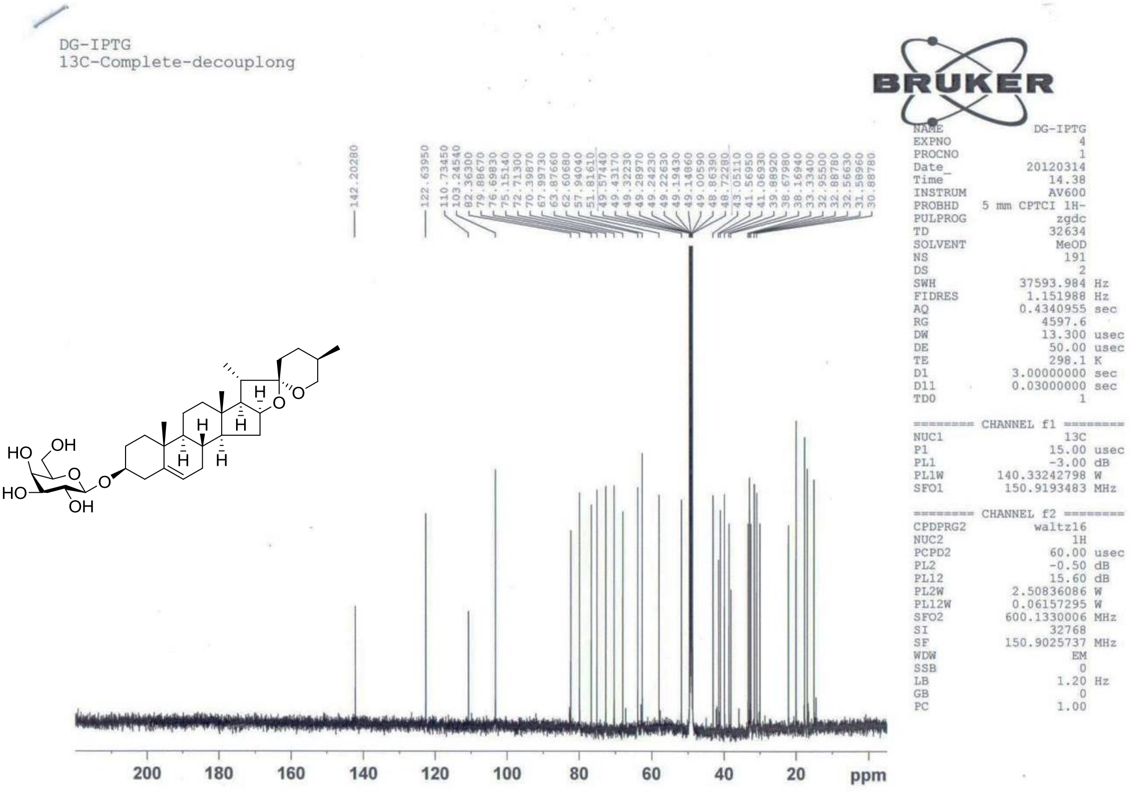

HH-COSY of Capsicoside A_3 (**8**)

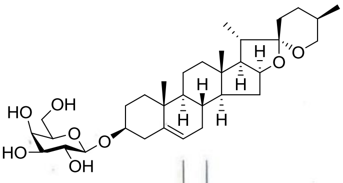

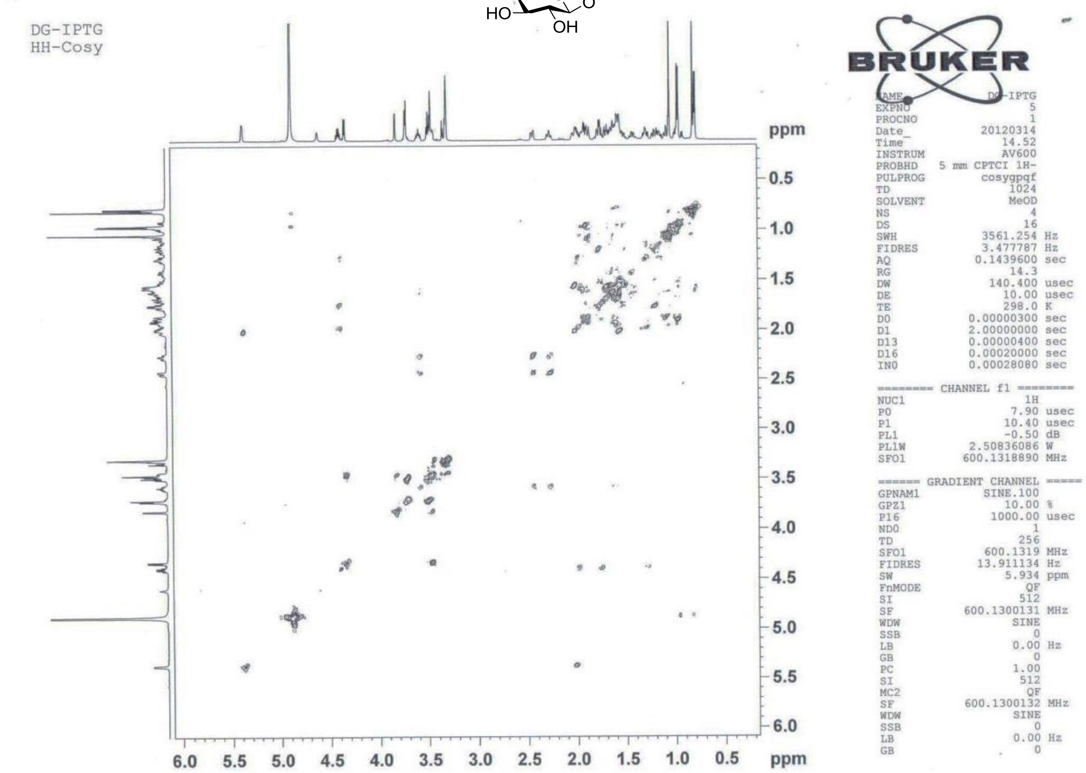

HSQC of Capsicoside A_3 (**8**)

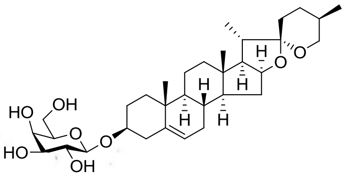

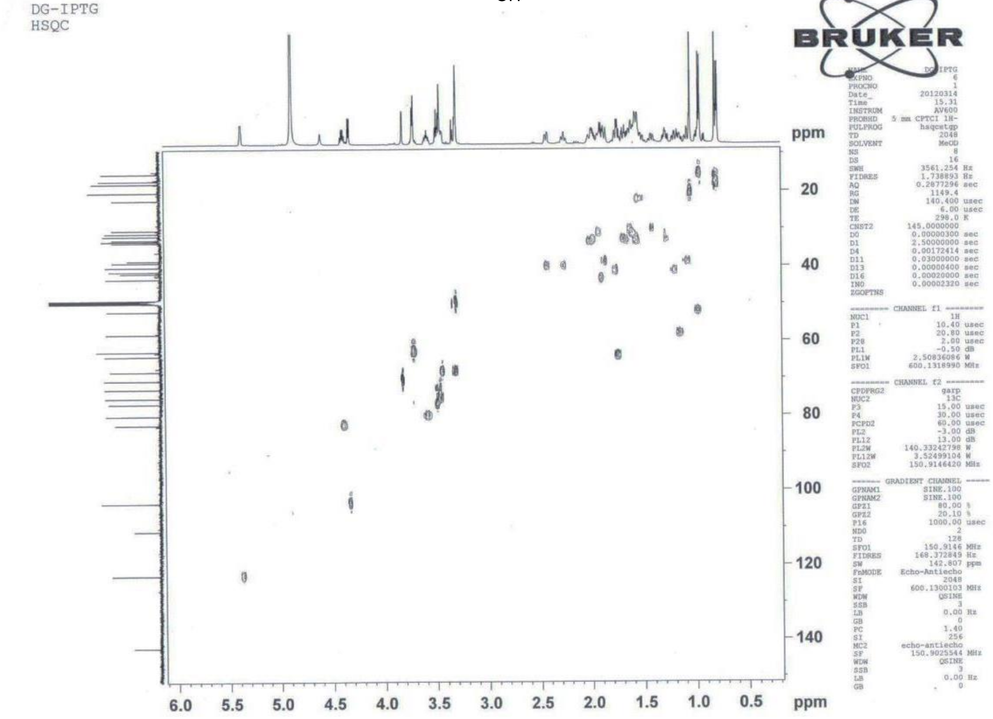

^{1}H NMR of Dihydrodioscin (**11**)

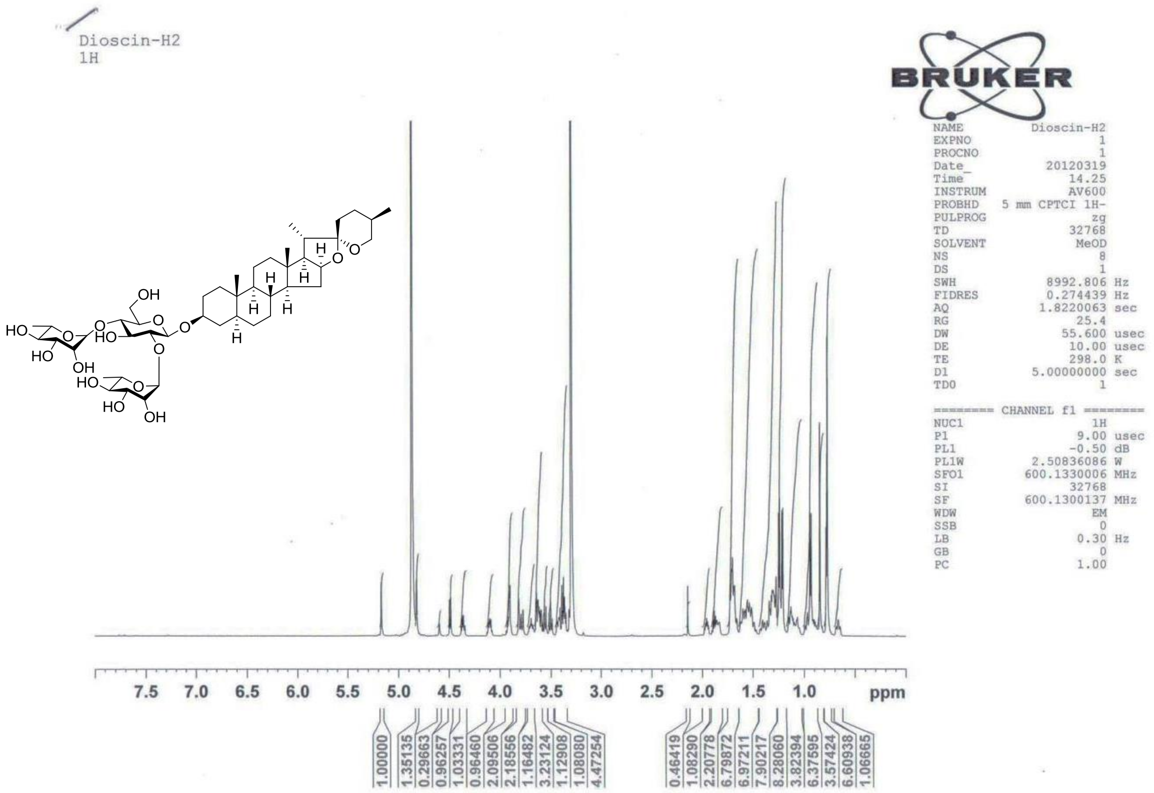

^{13}C NMR of Dihydrodioscin (**11**)

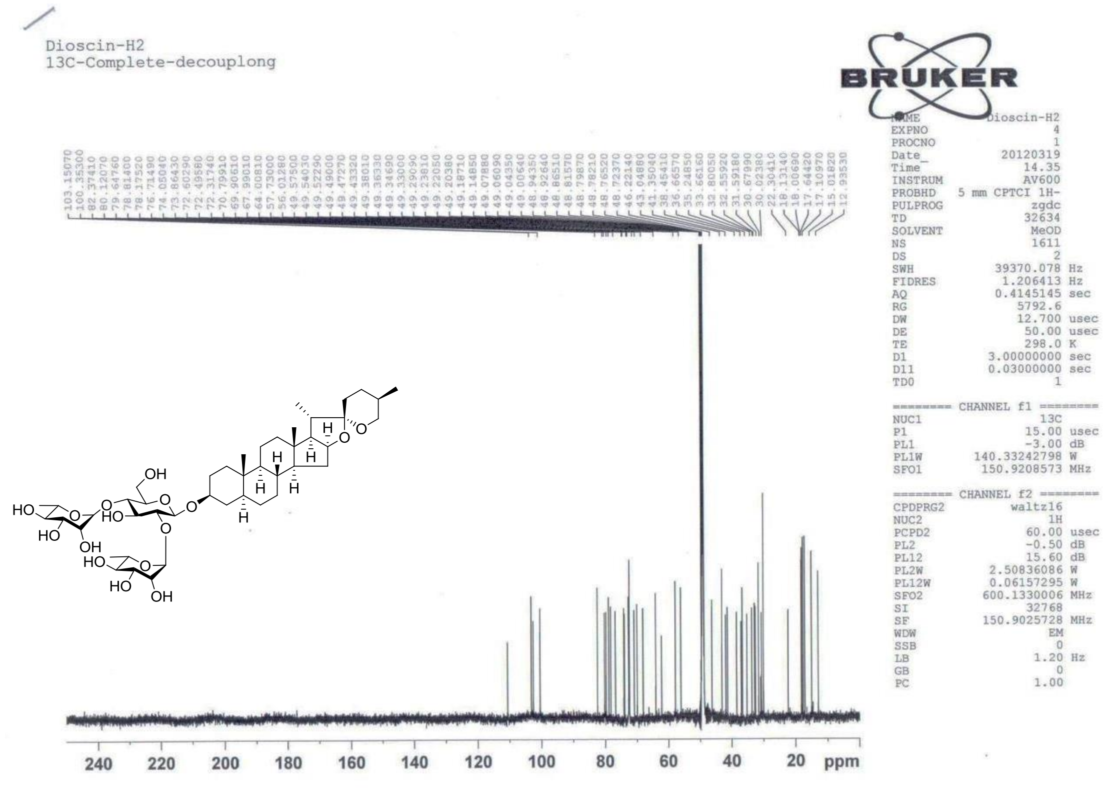

^{1}H NMR of Dihydropolyphyllin D (**12**)

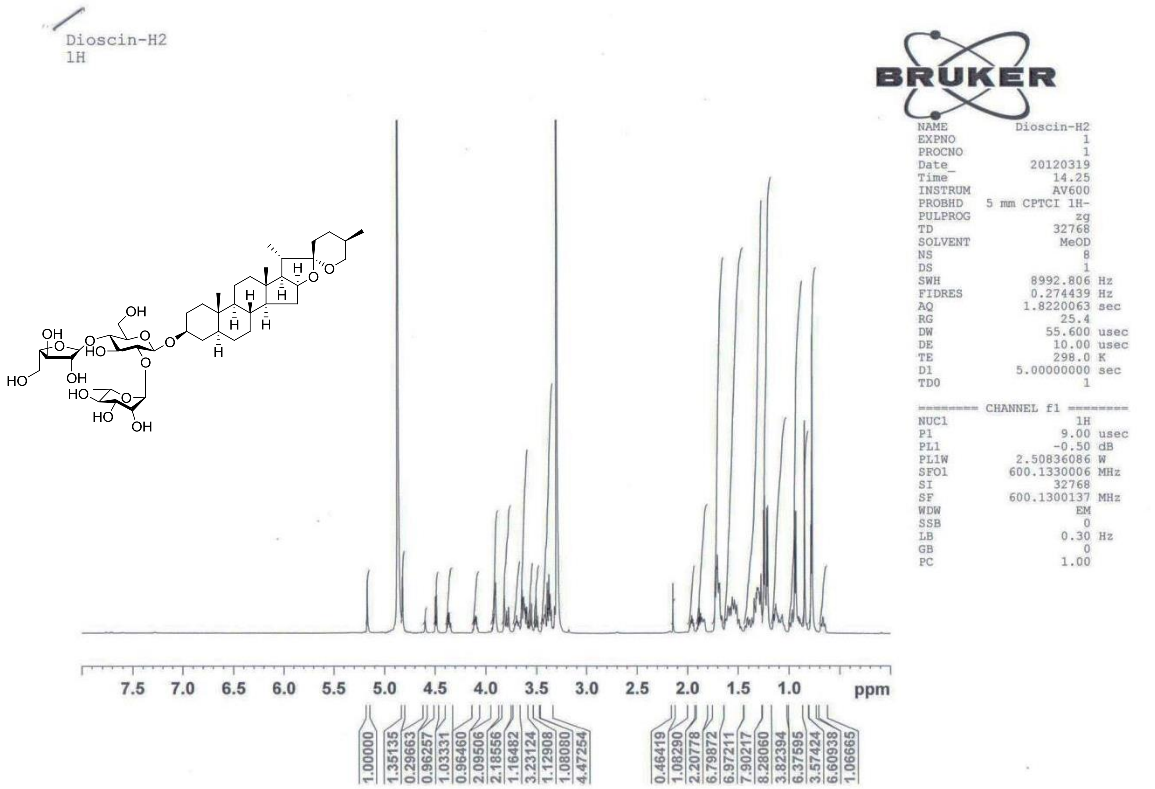

^{13}C NMR of Dihydropolyphyllin D (**12**)

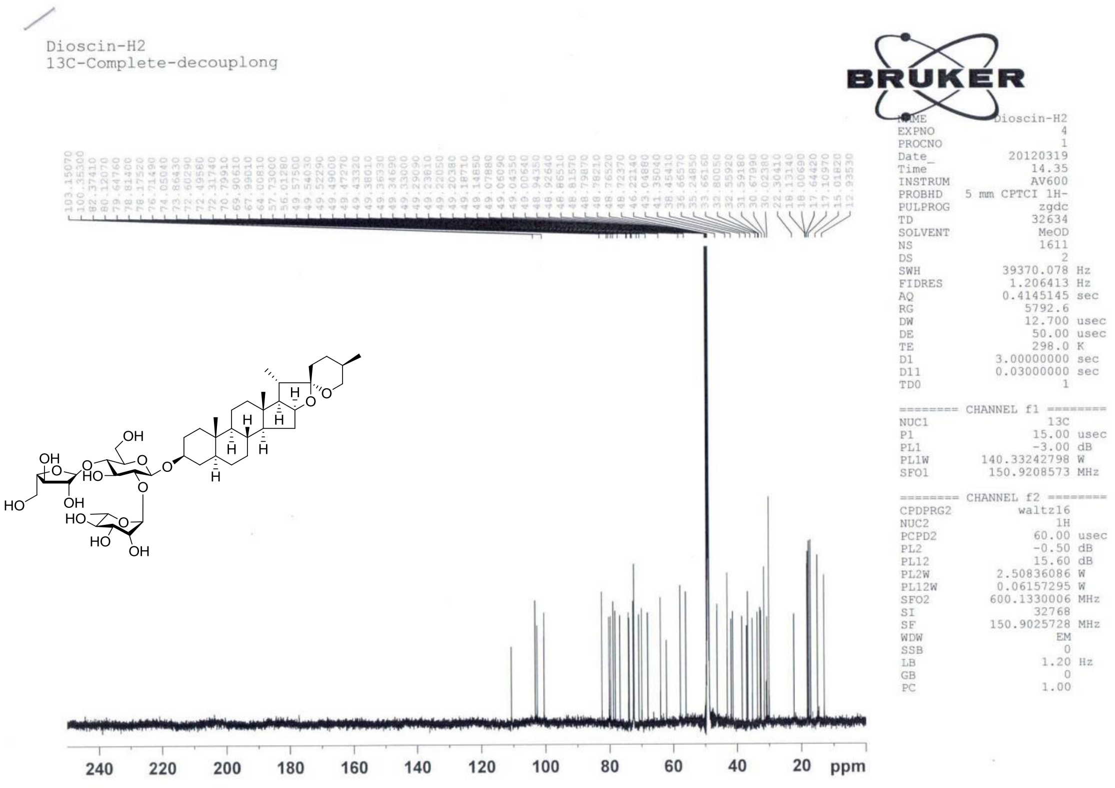

^{1}H NMR of Tigogenin (**13**)

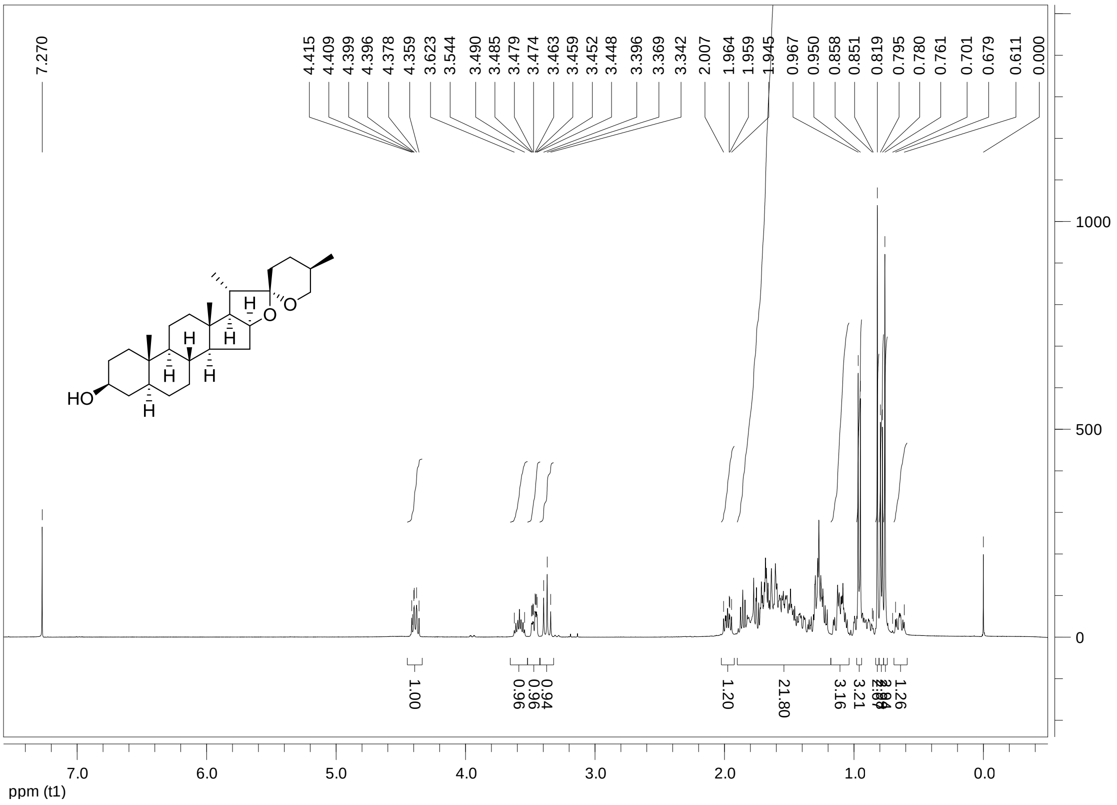

^{13}C NMR of Tigogenin (**13**)

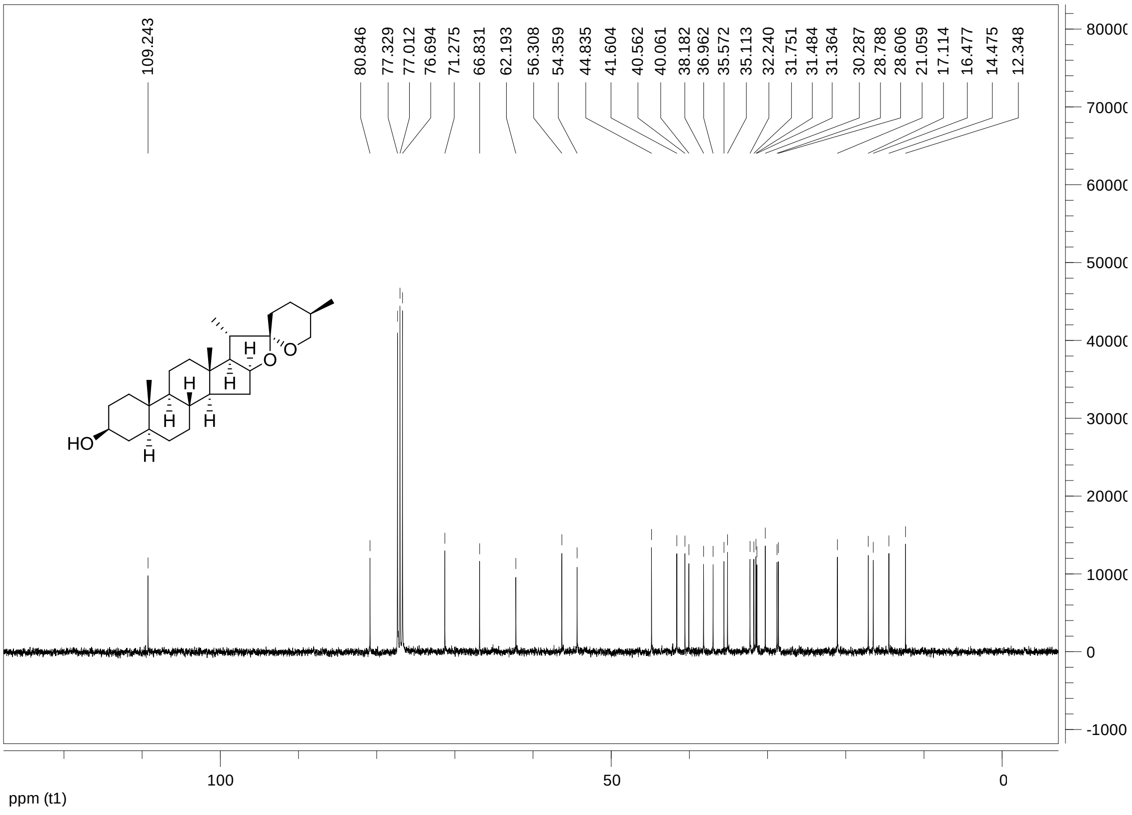

^{1}H NMR of Neotigogenin (**14**)

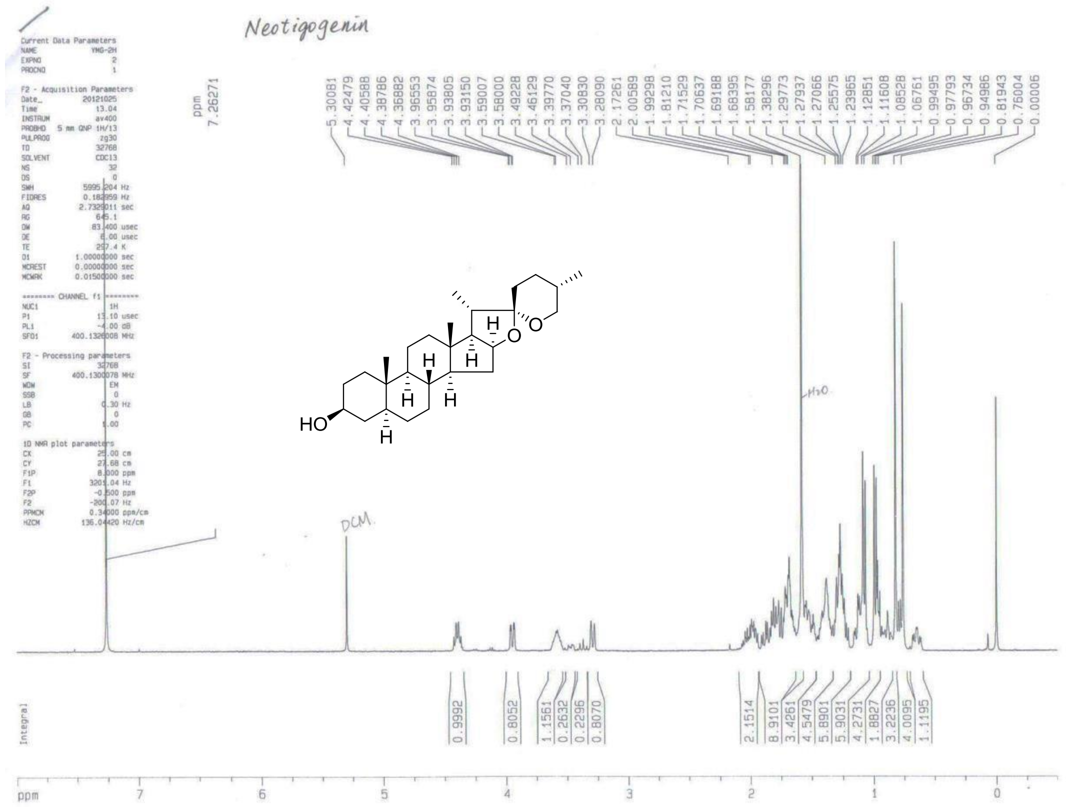

^{1}H NMR of 3-benzoylated diosgenin

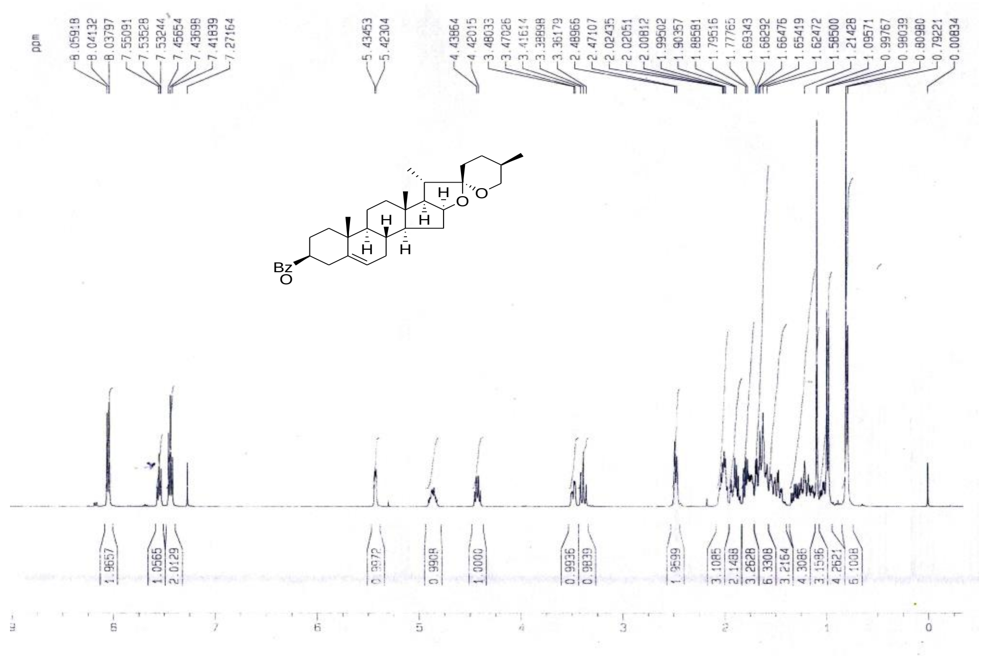

^{13}C NMR of 3-benzoylated diosgenin

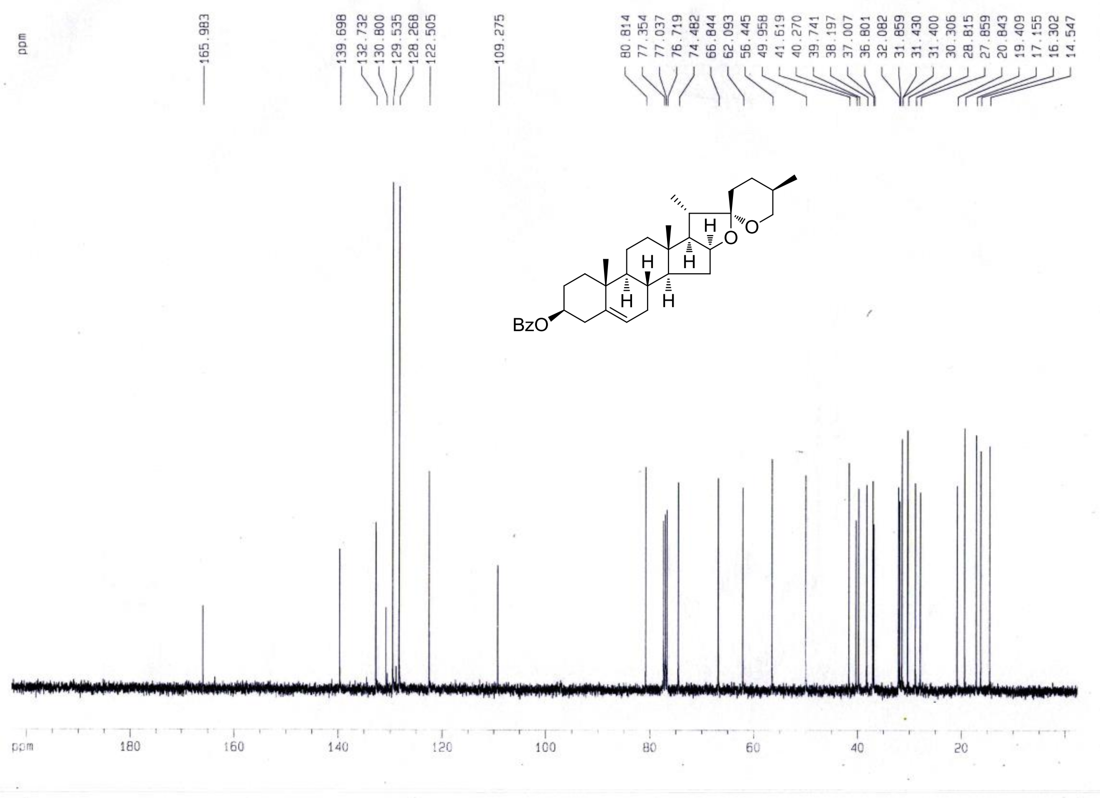

^{1}H NMR of 3-benzoylated 5α-H, 6α-OH diosgenin

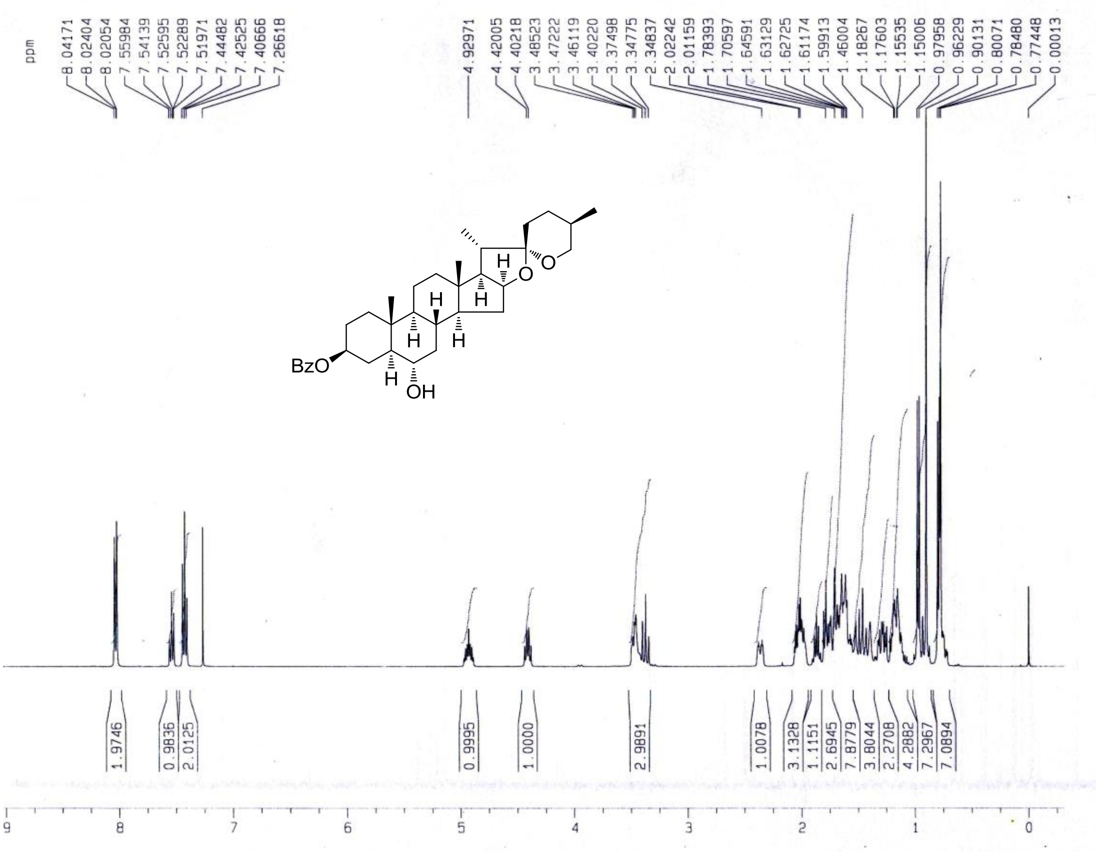

^{13}C NMR of 3-benzoylated 5α-H, 6α-OH diosgenin

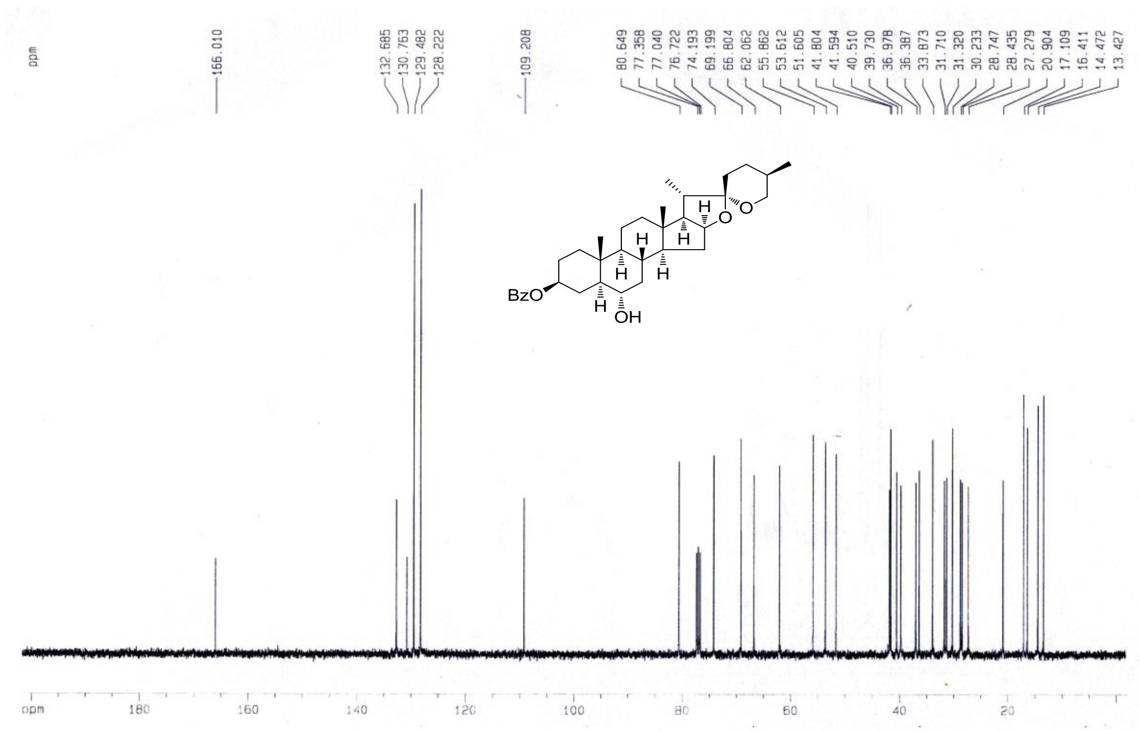

^{1}H NMR of 3-benzoylated 5β-H, 6β-OH diosgenin

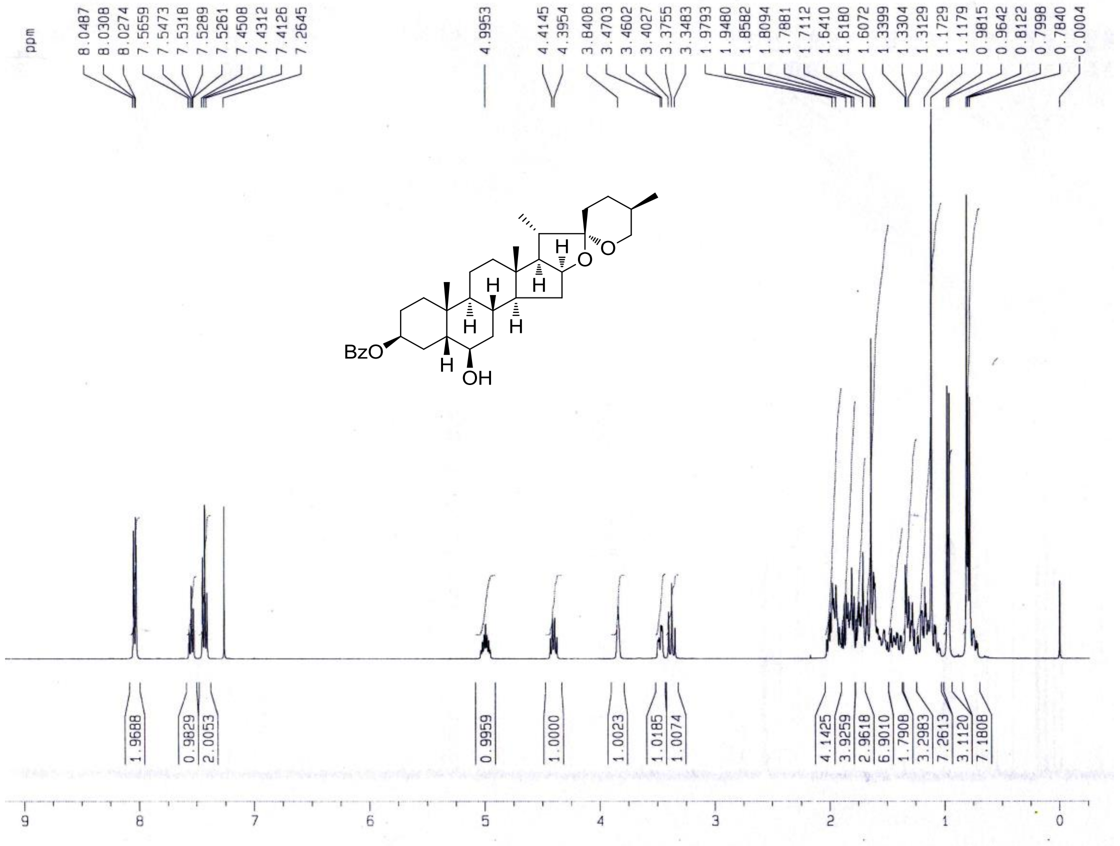

^{13}CNMR of 3-benzoylated 5β-H, 6β-OH diosgenin

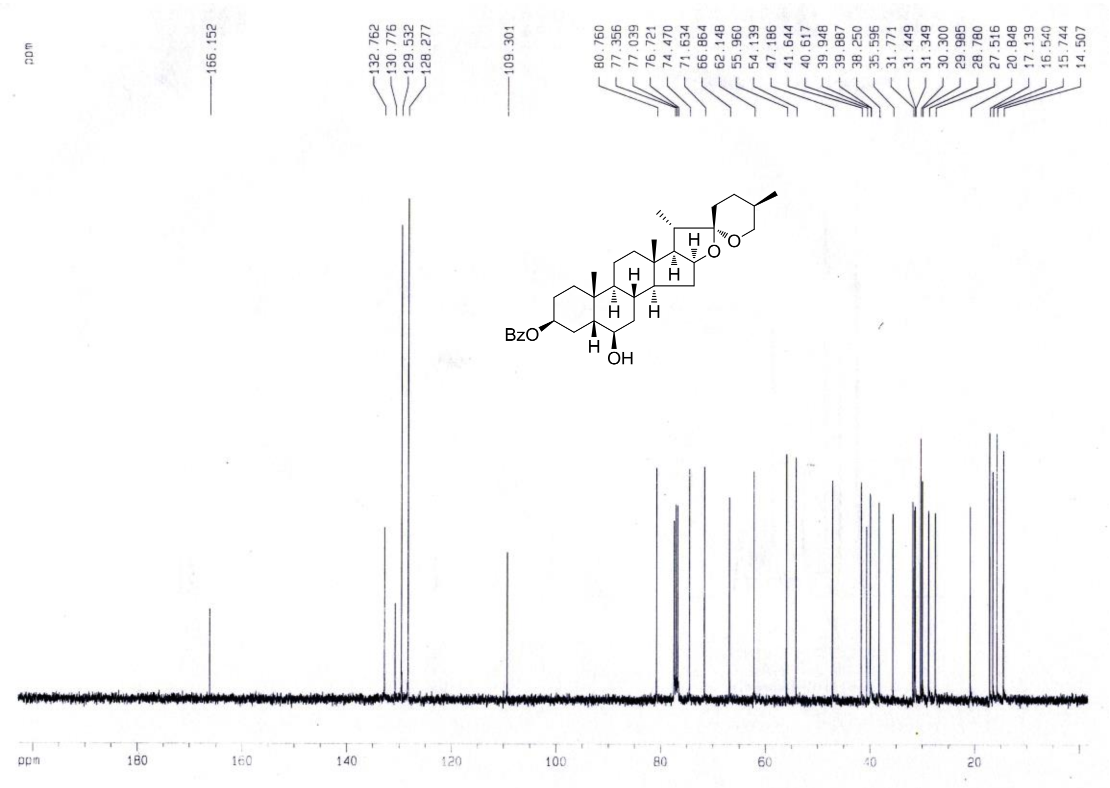

^{1}H NMR of 5α-H, 6α-OH diosgenin (**15**)

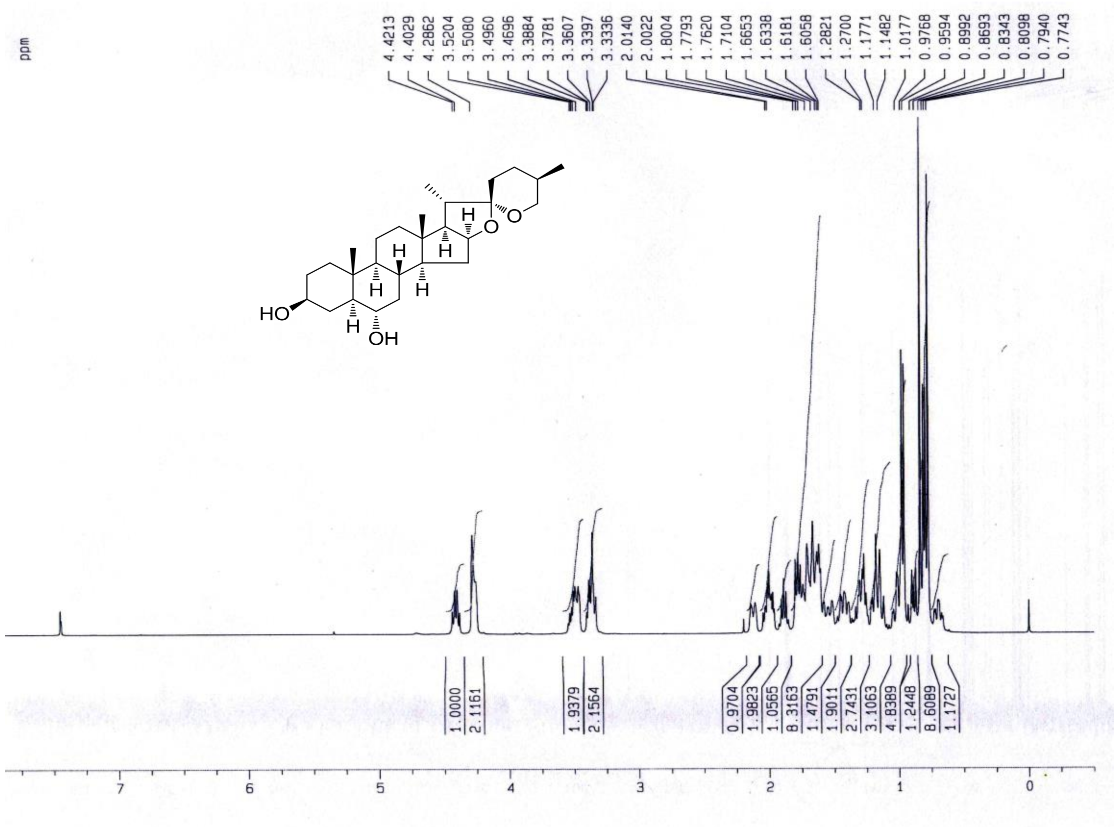

^{13}C NMR of 5α-H, 6α-OH diosgenin (**15**)

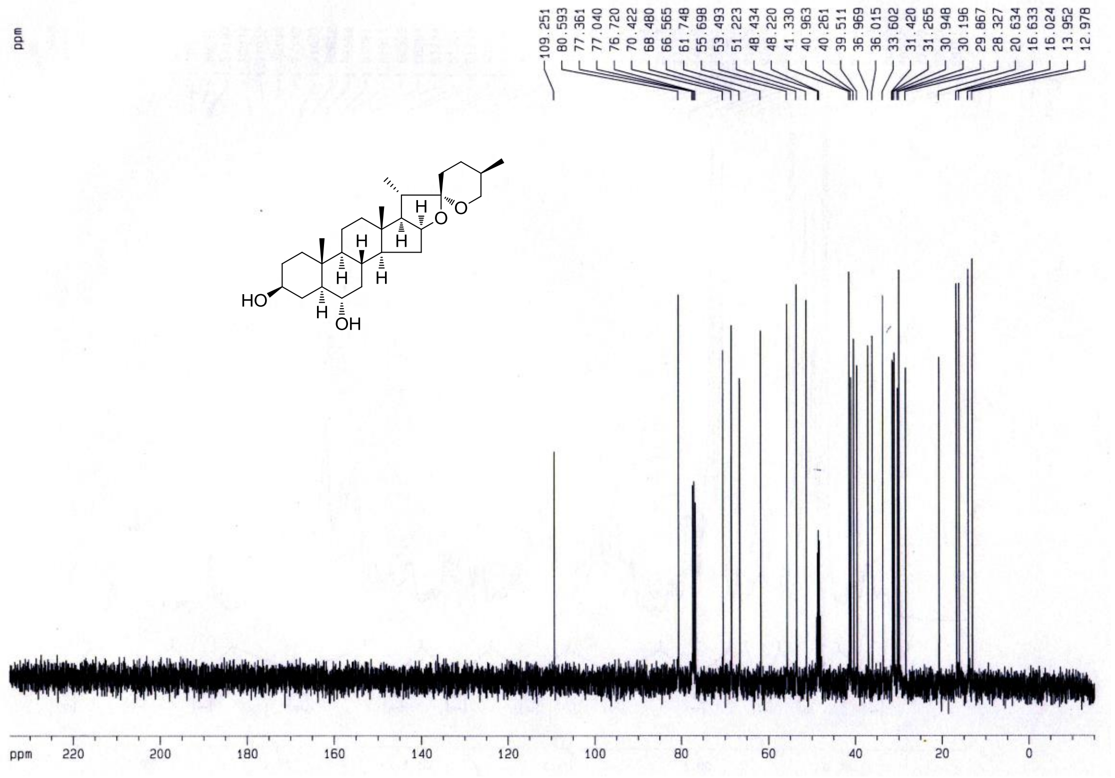

^{1}H NMR of 5β-H, 6β-OH diosgenin (**16**)

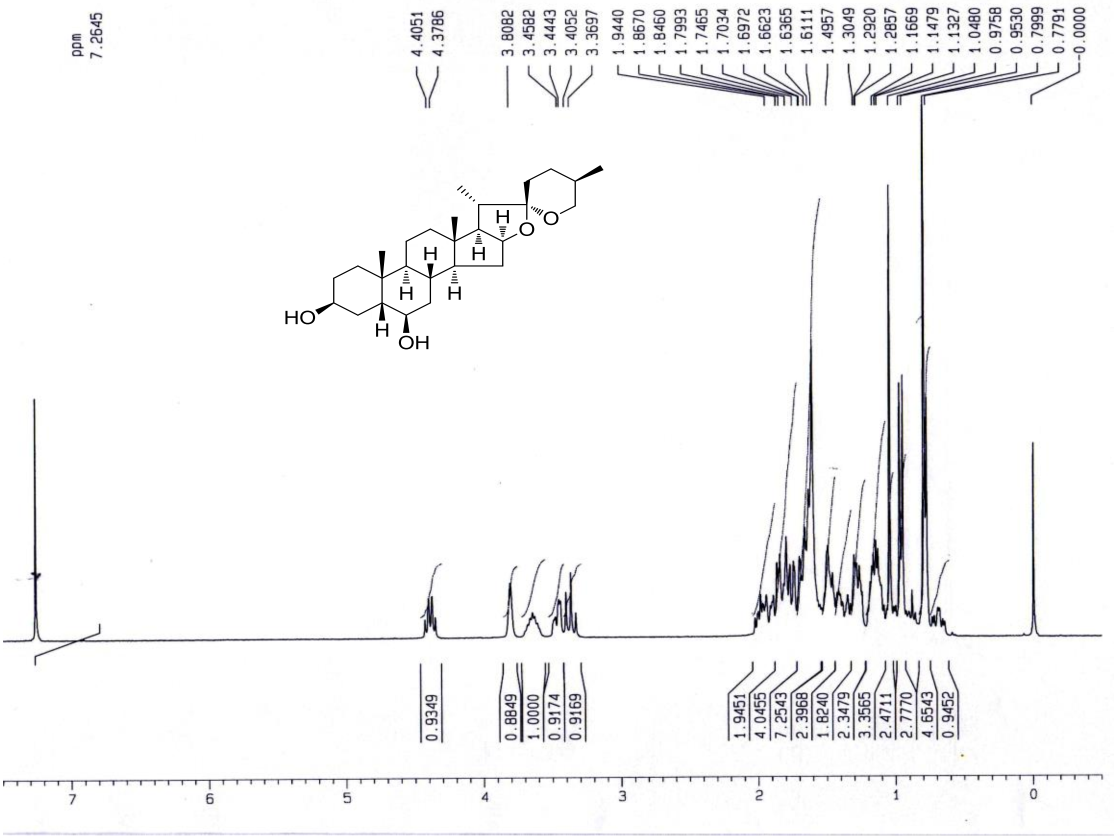

^{13}C NMR of 5β-H, 6β-OH diosgenin (**16**)

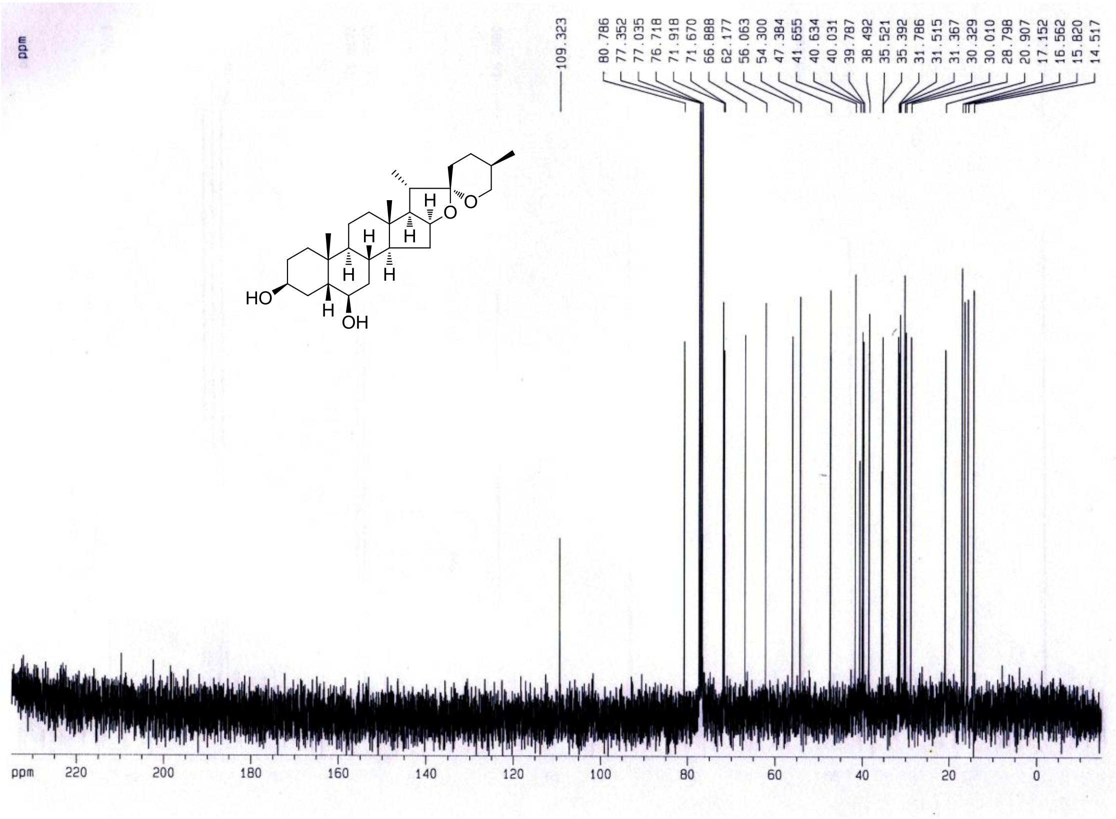

^{1}H NMR of 5α-H, 6α-OH yamogenin (**17**)

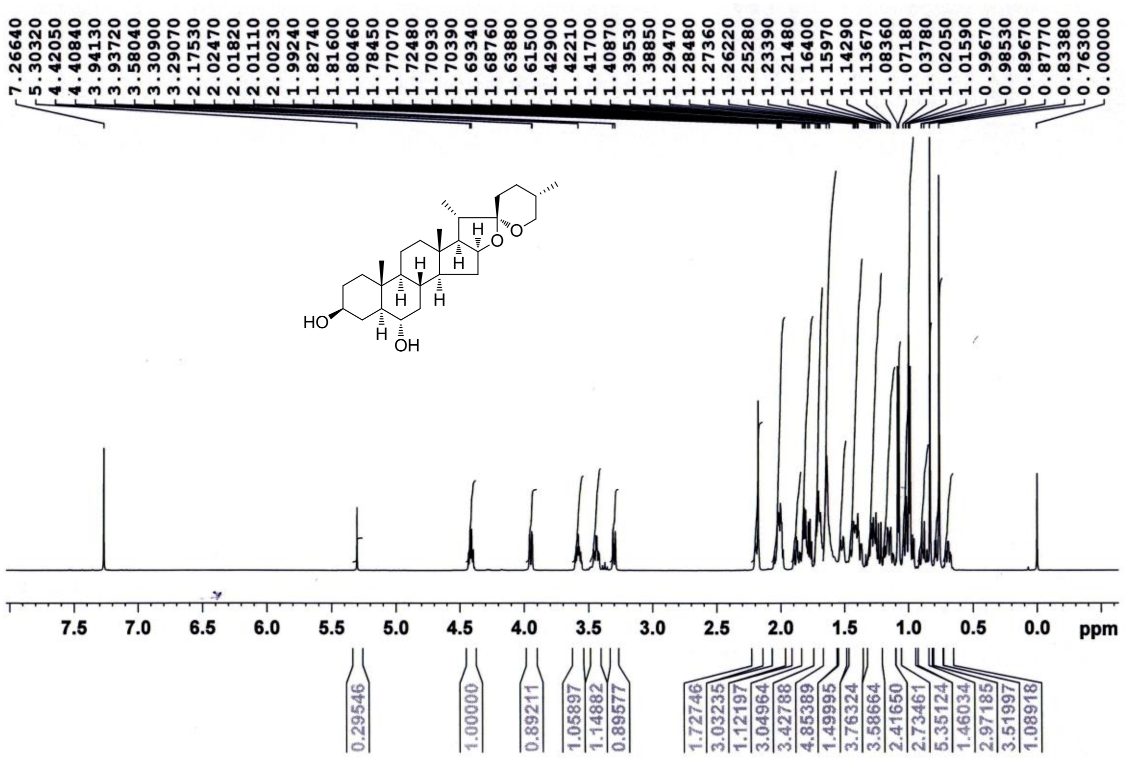

^{13}C NMR of 5α-H, 6α-OH yamogenin (**17**)

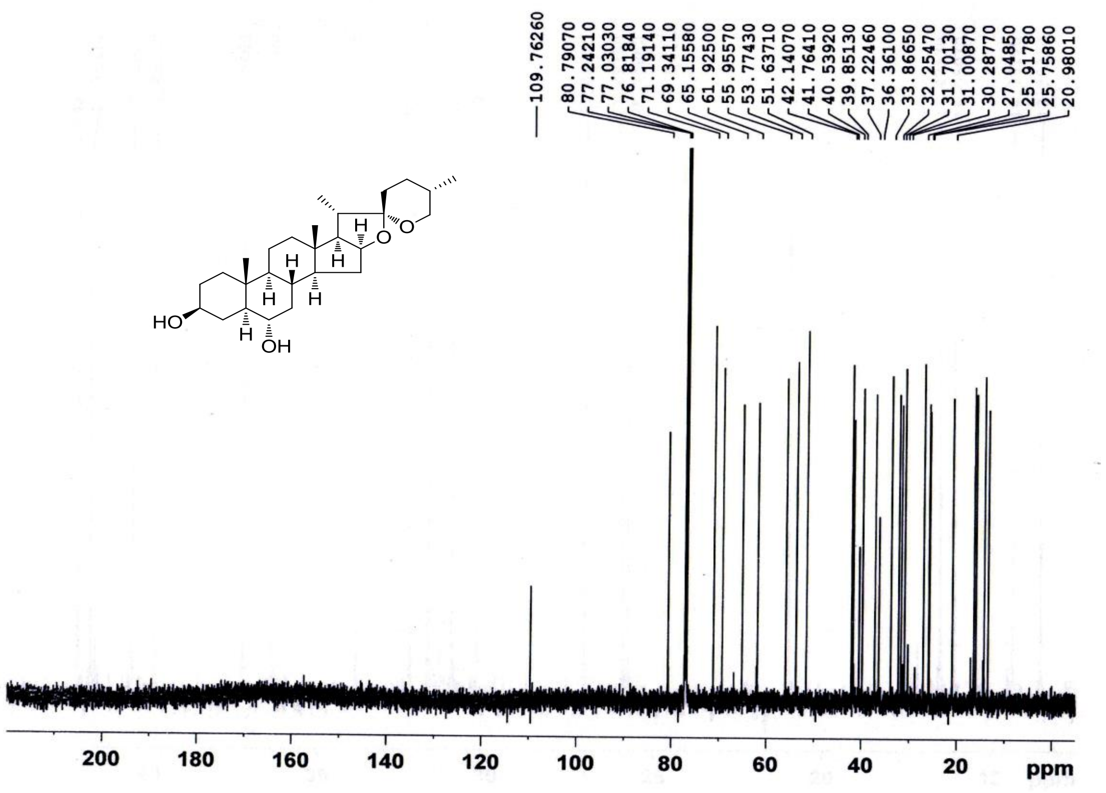

NOE of 5α-H 6α-OH yamogenin (**17**)

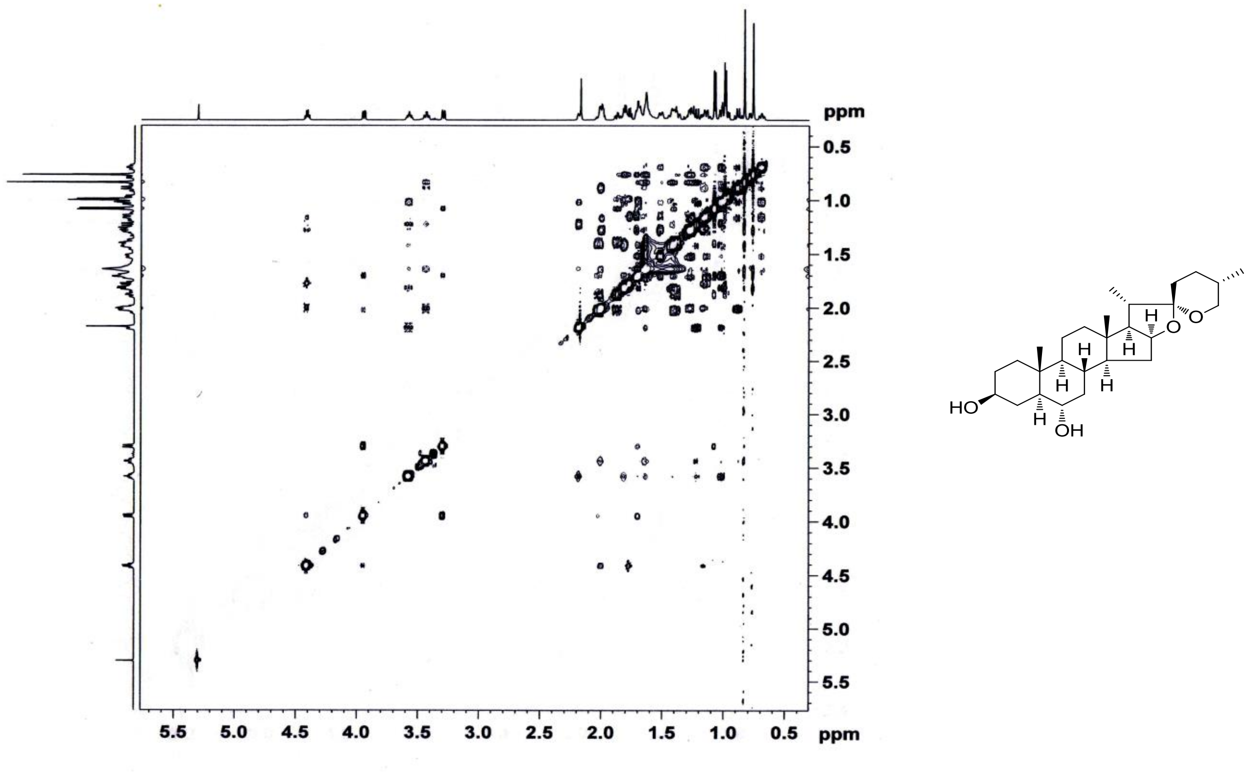

^{1}H NMR of 5β-H, 6β-OH yamogenin (**18**)

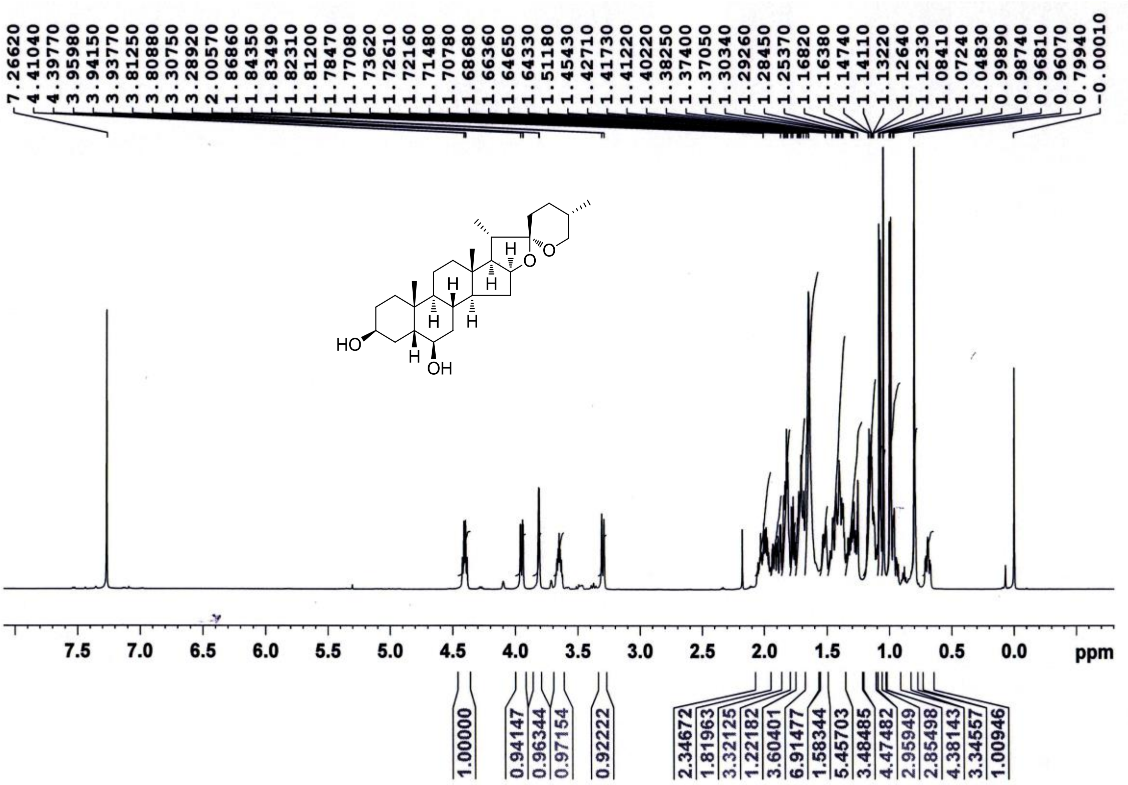

^{13}C NMR of 5β-H, 6β-OH yamogenin (**18**)

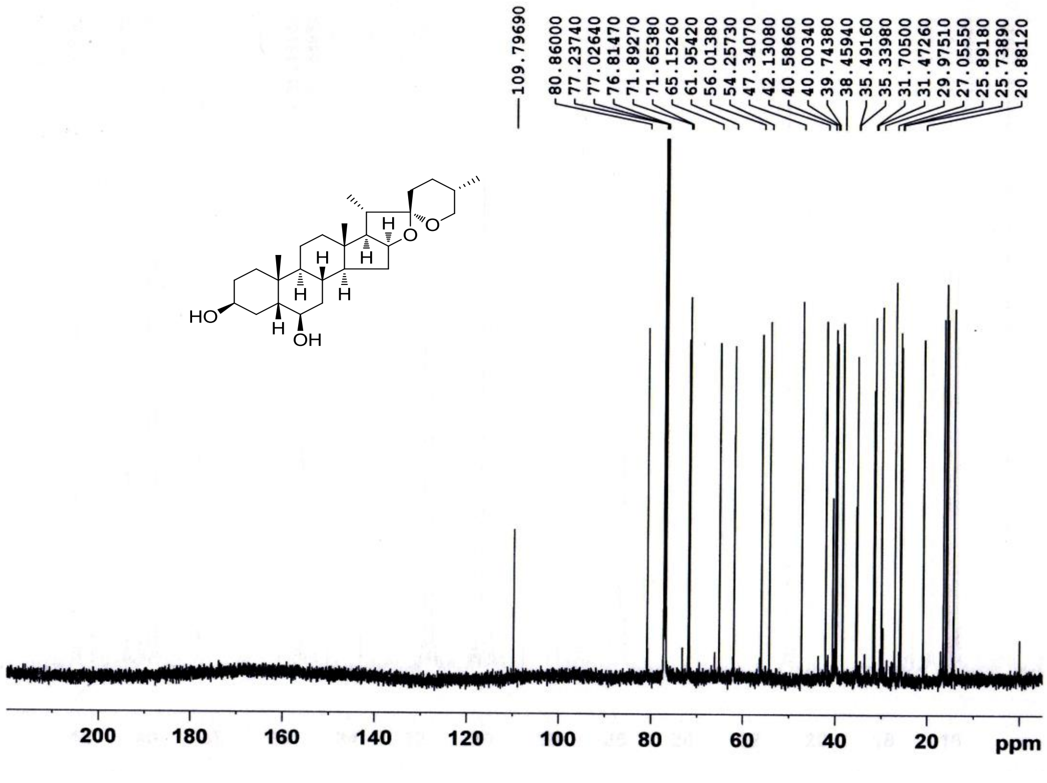

^{1}H NMR of Sarsasapogenone (**19**)

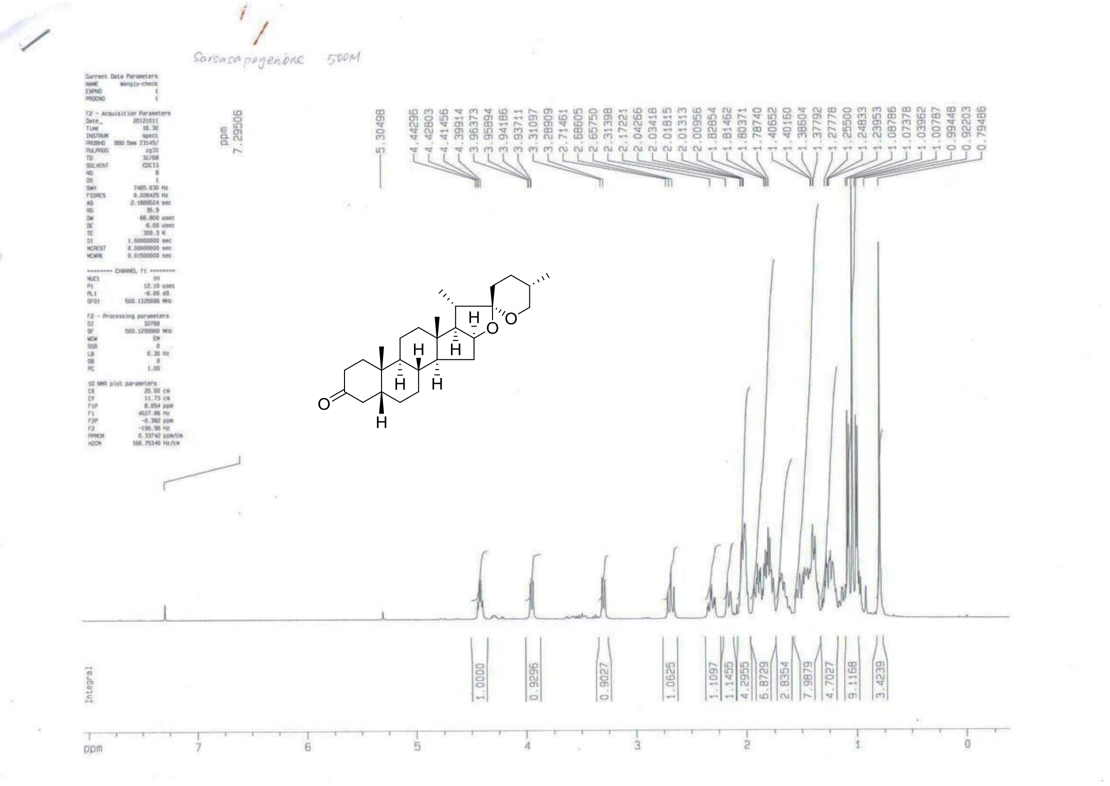

^{13}C NMR of Sarsasapogenone (**19**)

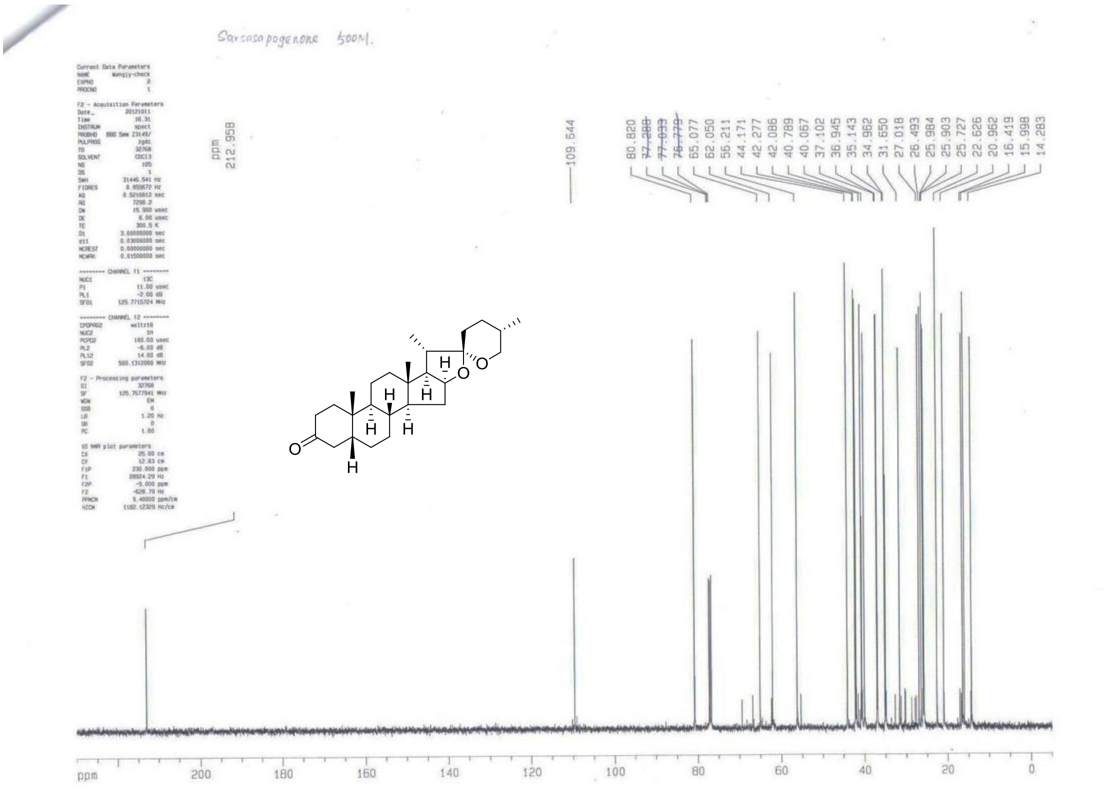

Dept-135 of Sarsasapogenone (**19**)

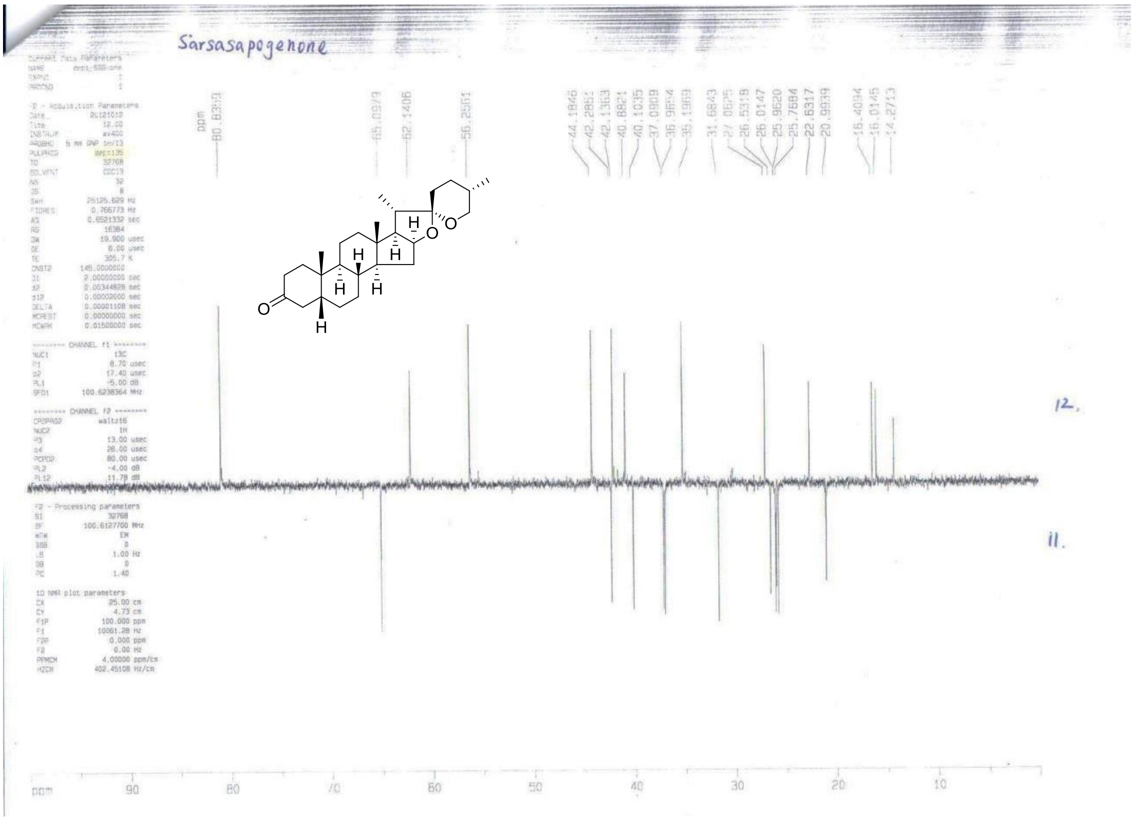

^{1}H NMR of Episarsasapogenin (**20**)

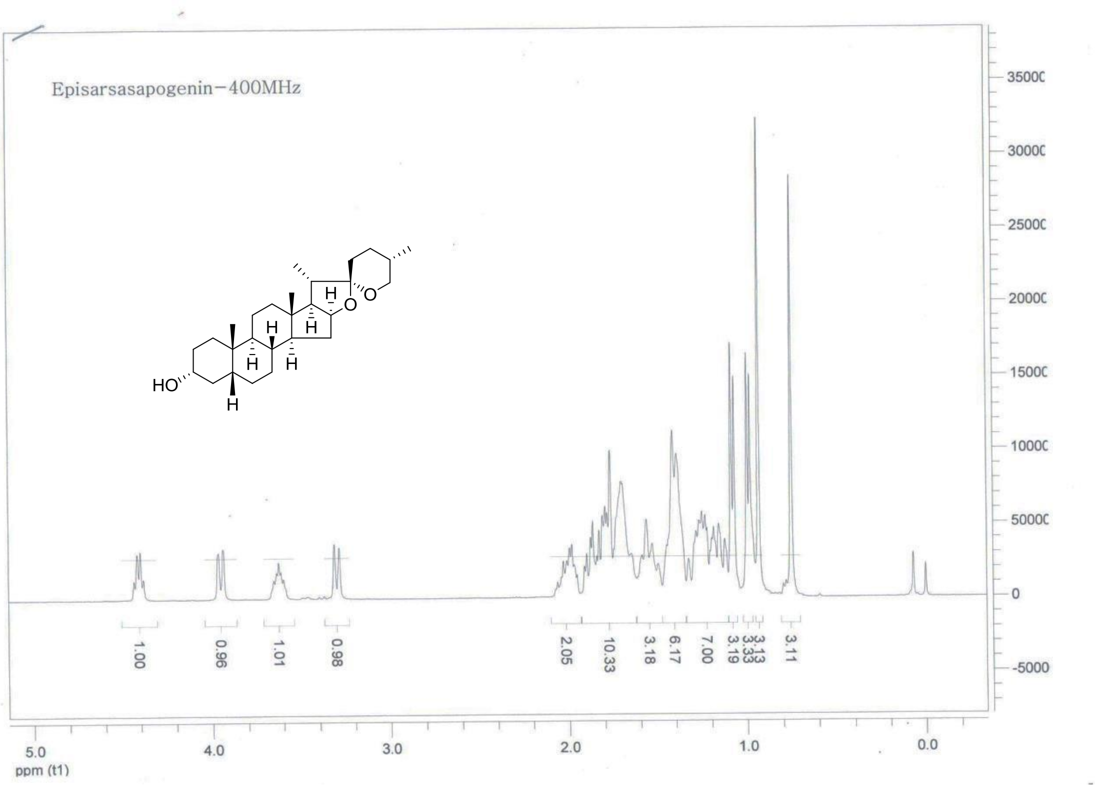

^{13}C NMR of Episarsasapogenin (**20**)

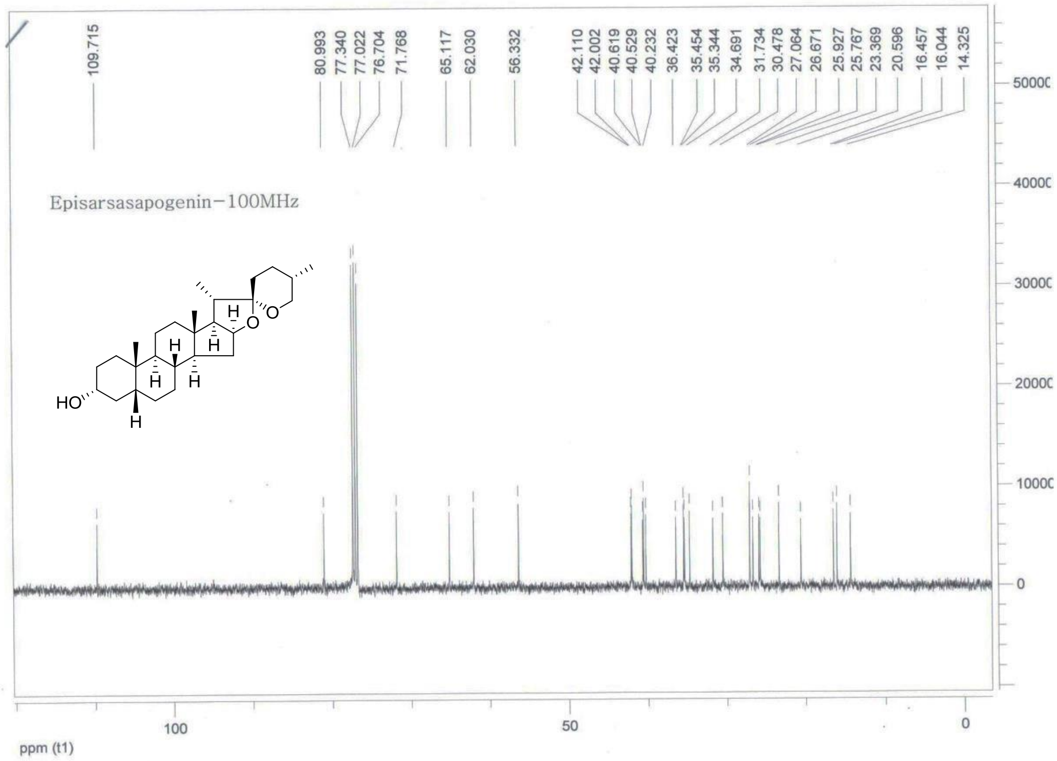

^{1}H NMR of dihydrosarsasapogenin (dSSG, **21**)

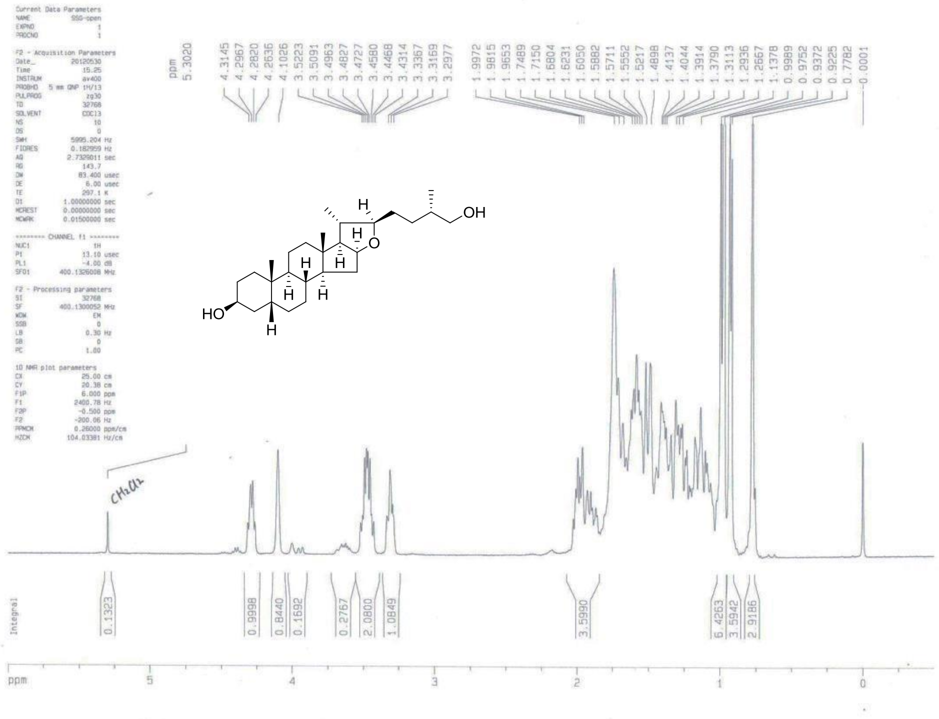

^{13}C NMR of dihydrosarsasapogenin (dSSG, **21**)

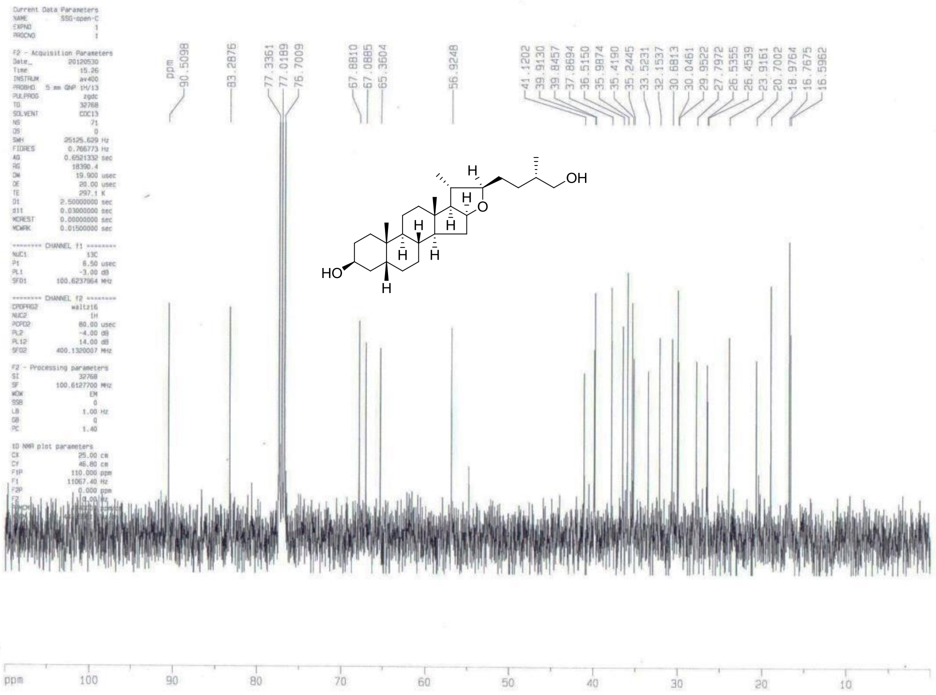

^{1}H NMR of Propargyl SSG (**25**)

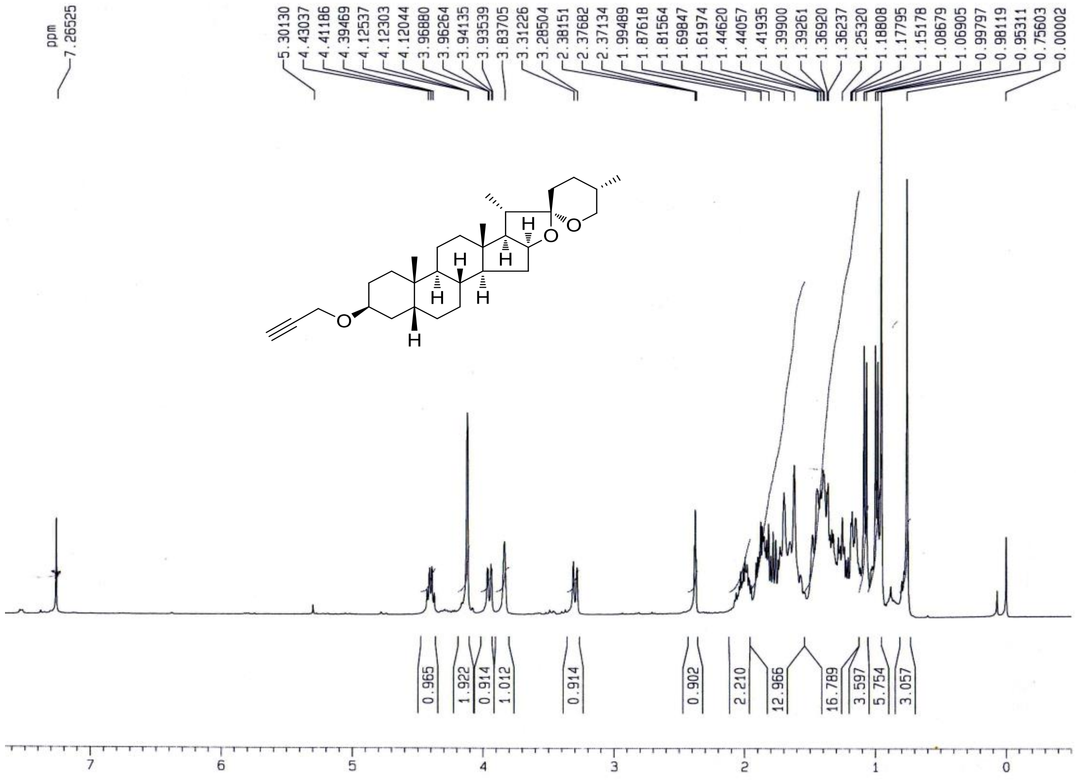

^{13}C NMR of Propargyl SSG (**25**)

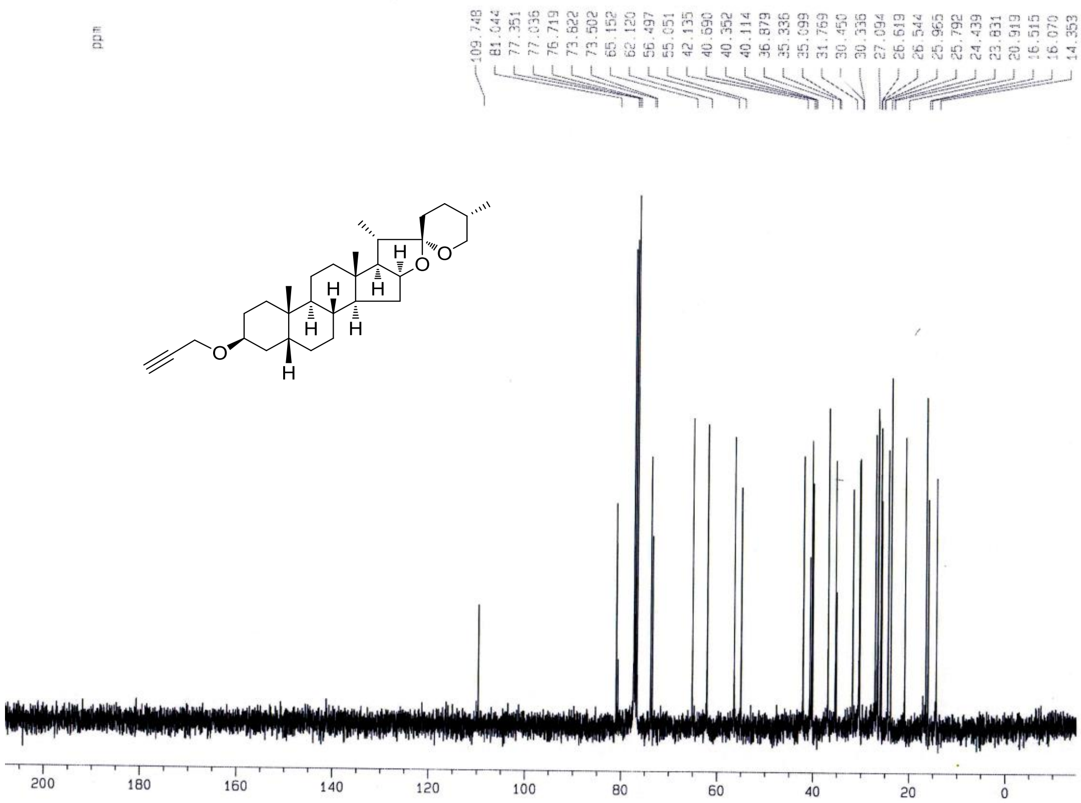

^{1}H NMR of Compound **26a**

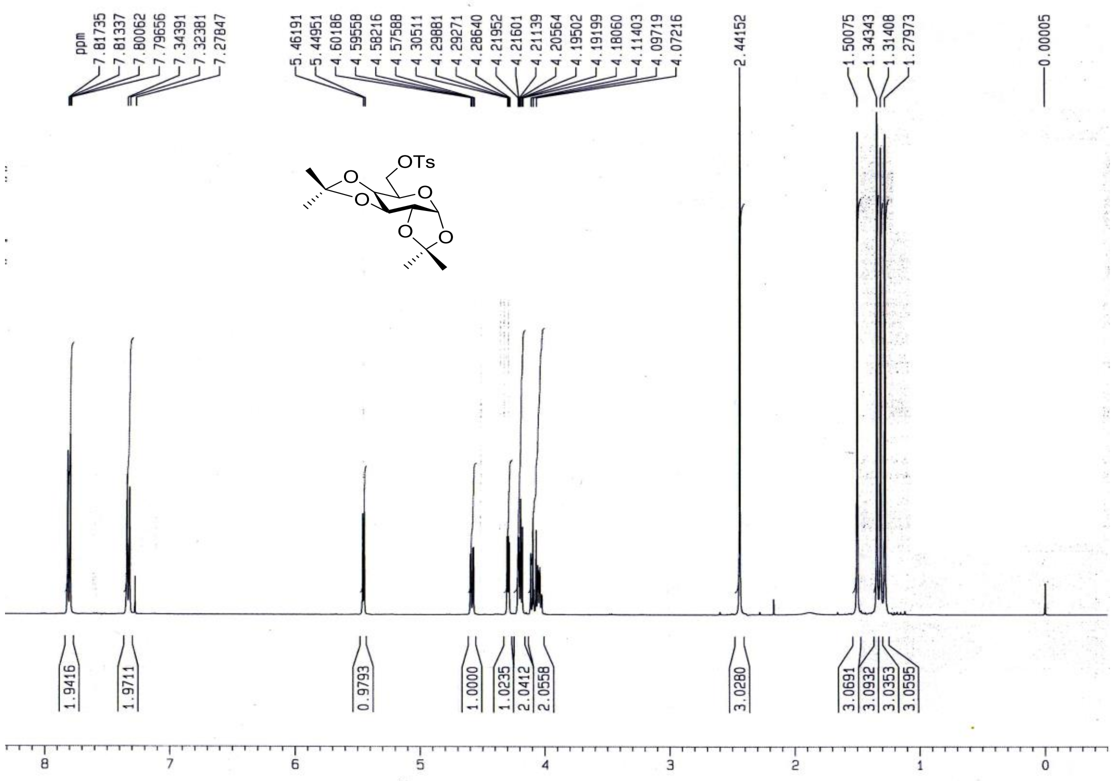

^{13}C NMR of Compound **26a**

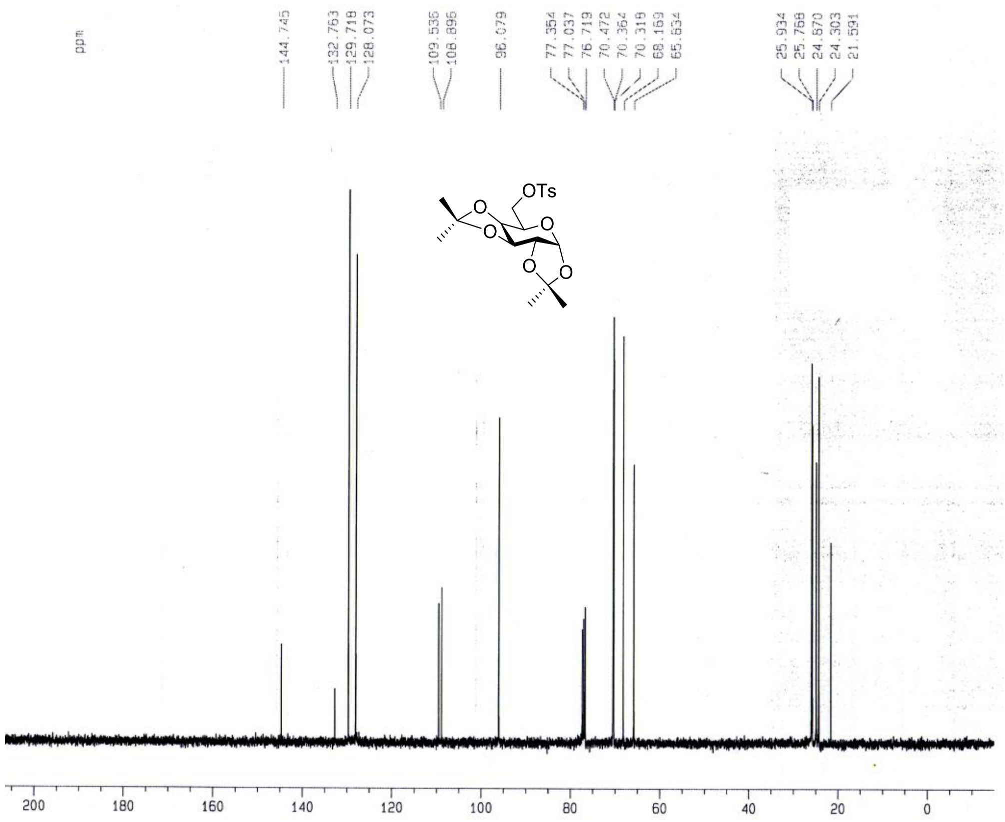

^{1}H NMR of Compound **26**

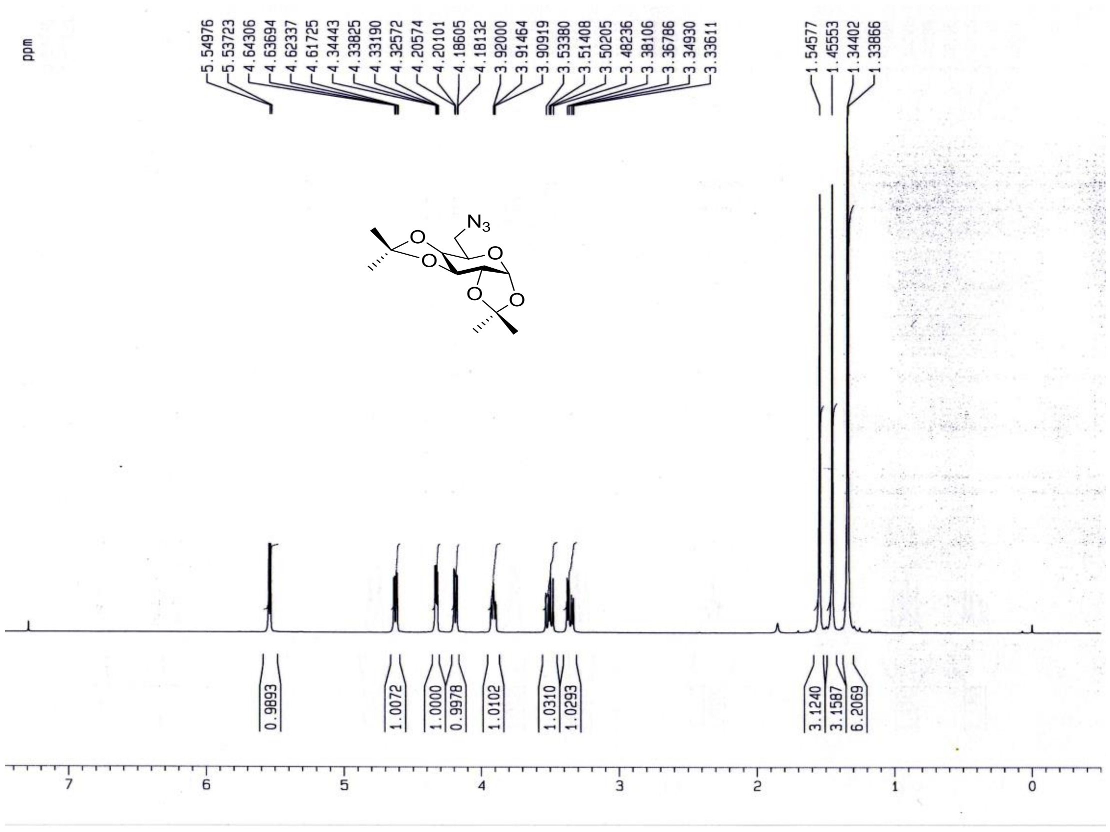

^{13}C NMR of Compound **26**

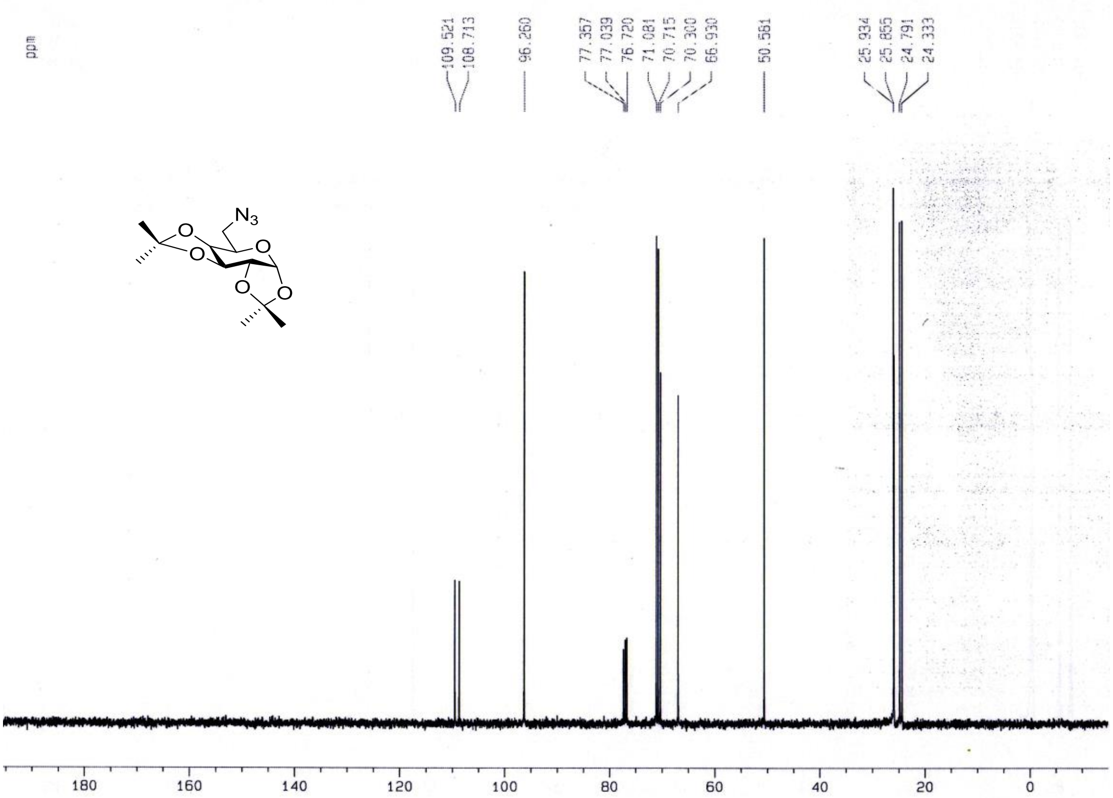

^{1}H NMR of Triazole SSG (**27**)

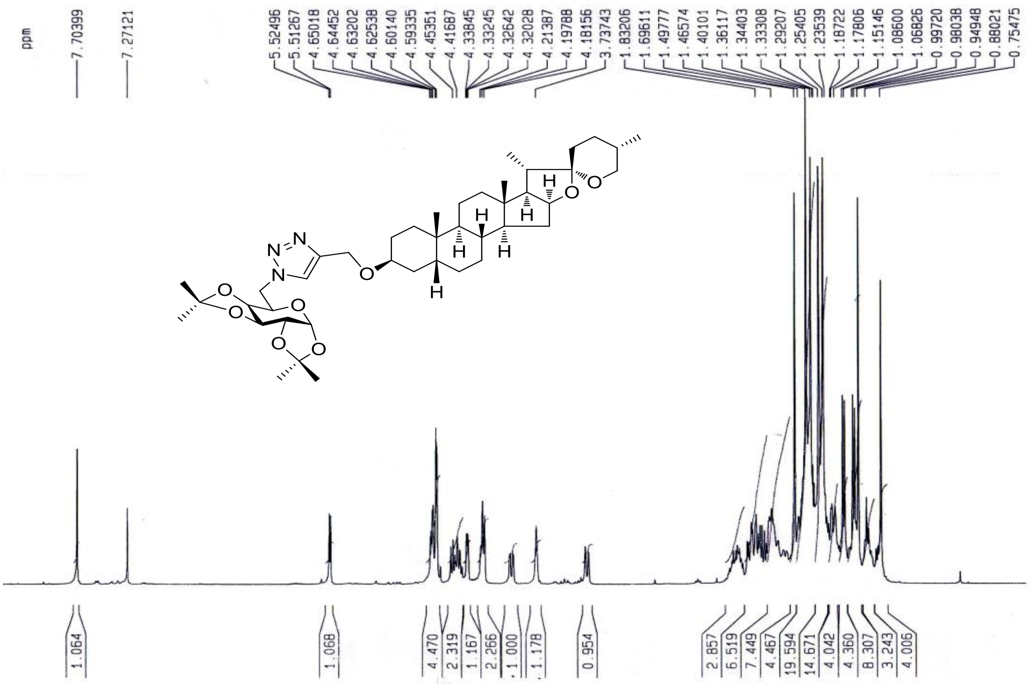

^{13}C NMR of Triazole SSG (**27**)

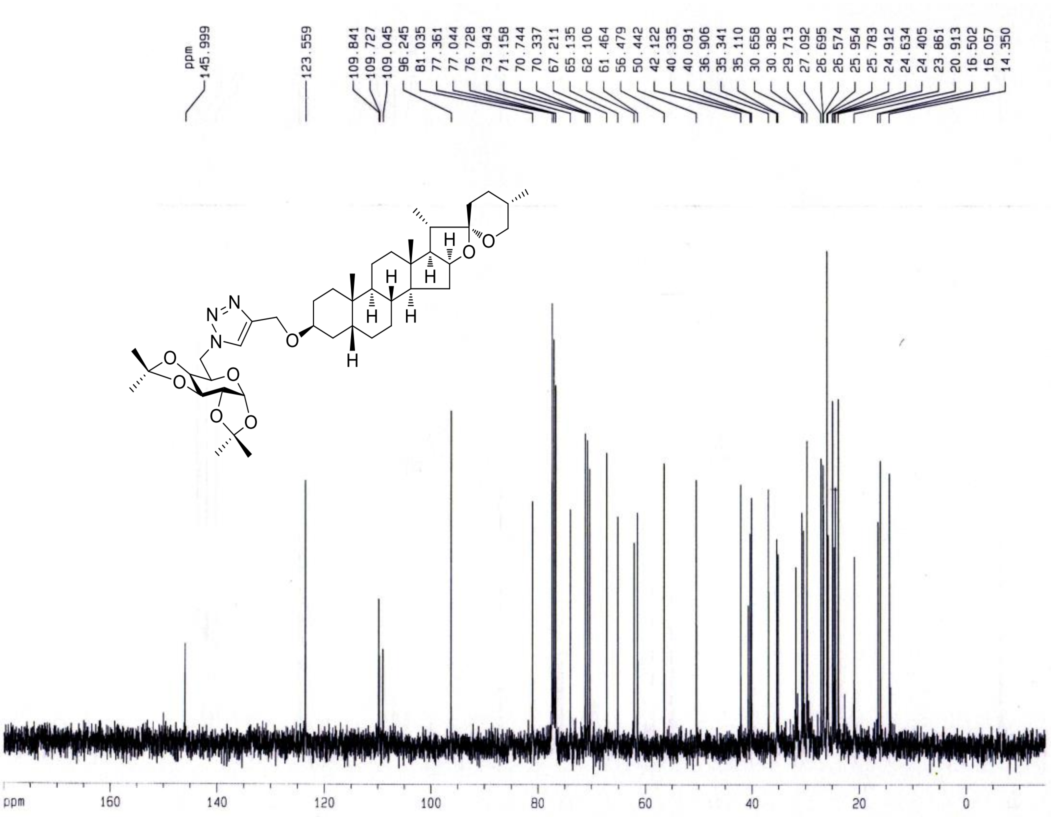

^{1}H NMR of α-OMe triazole SSG (**28**)

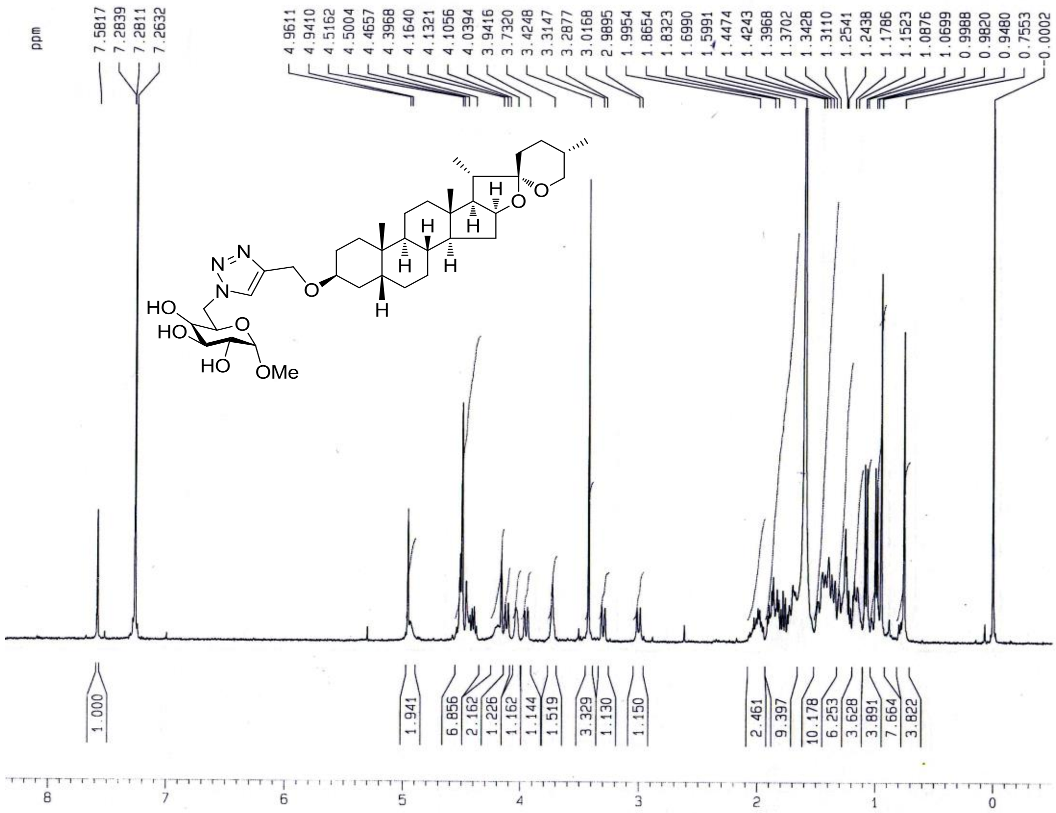

^{13}C NMR of α-OMe triazole SSG (**28**)

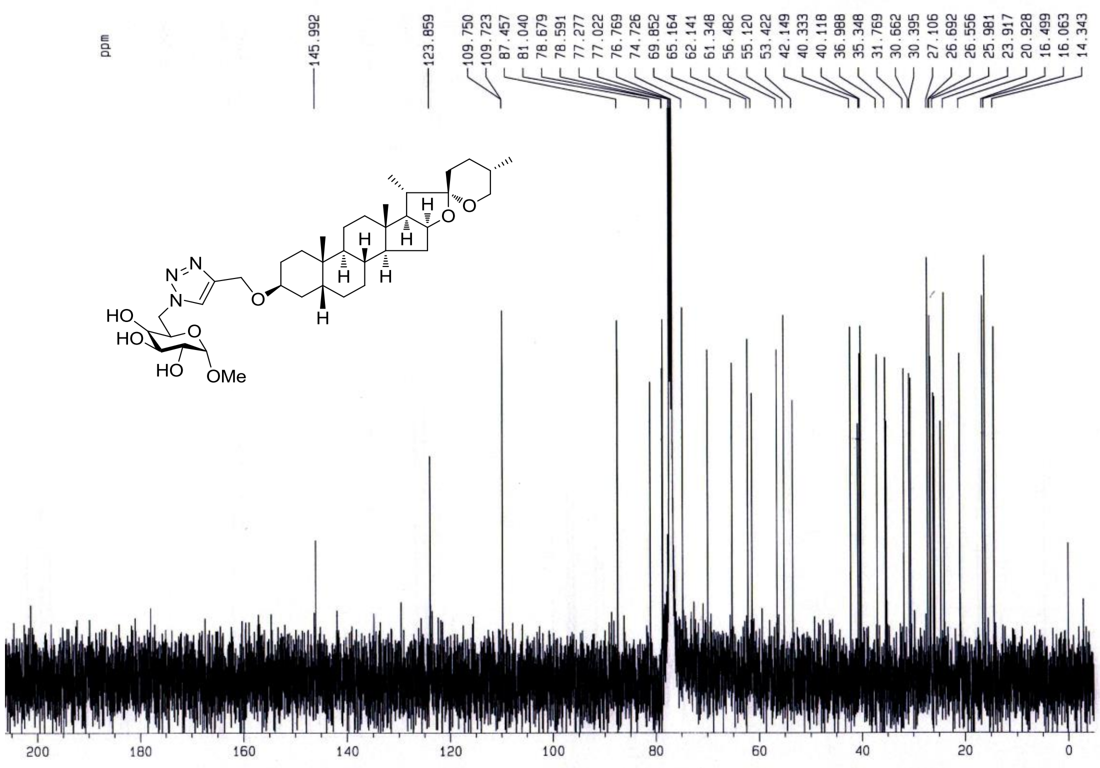

^{1}H NMR of Carboxylate ethereal SSG (**29a**)

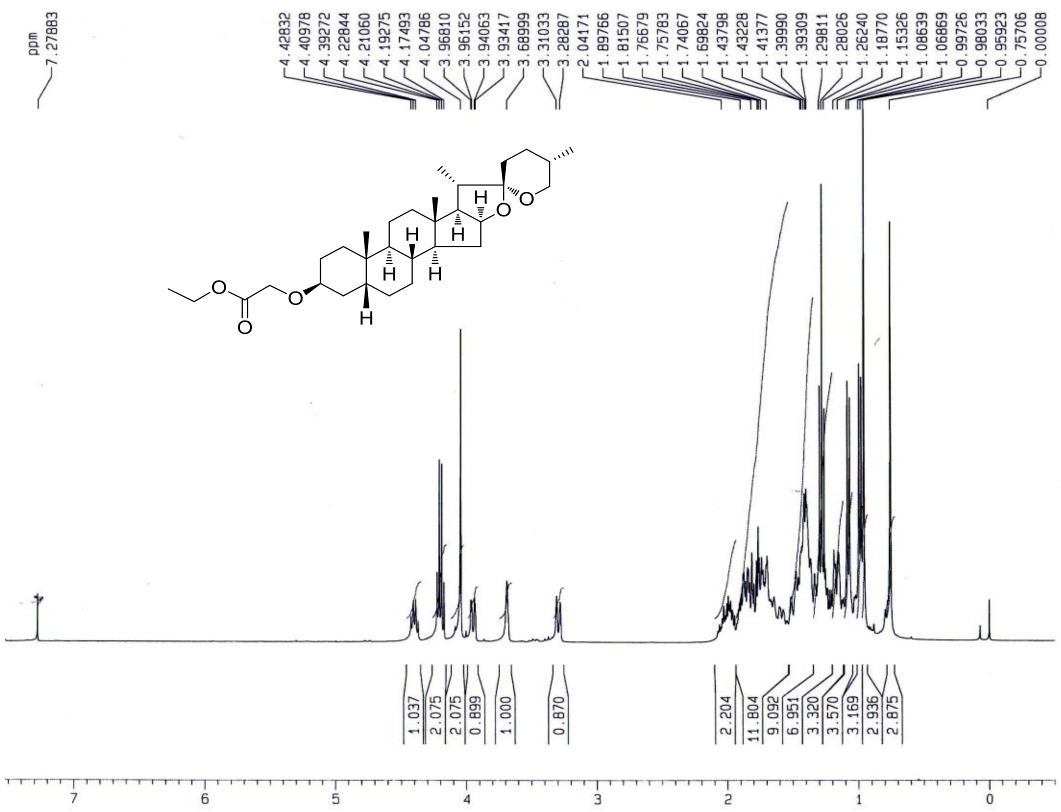

^{13}C NMR of Carboxylate ethereal SSG (**29a**)

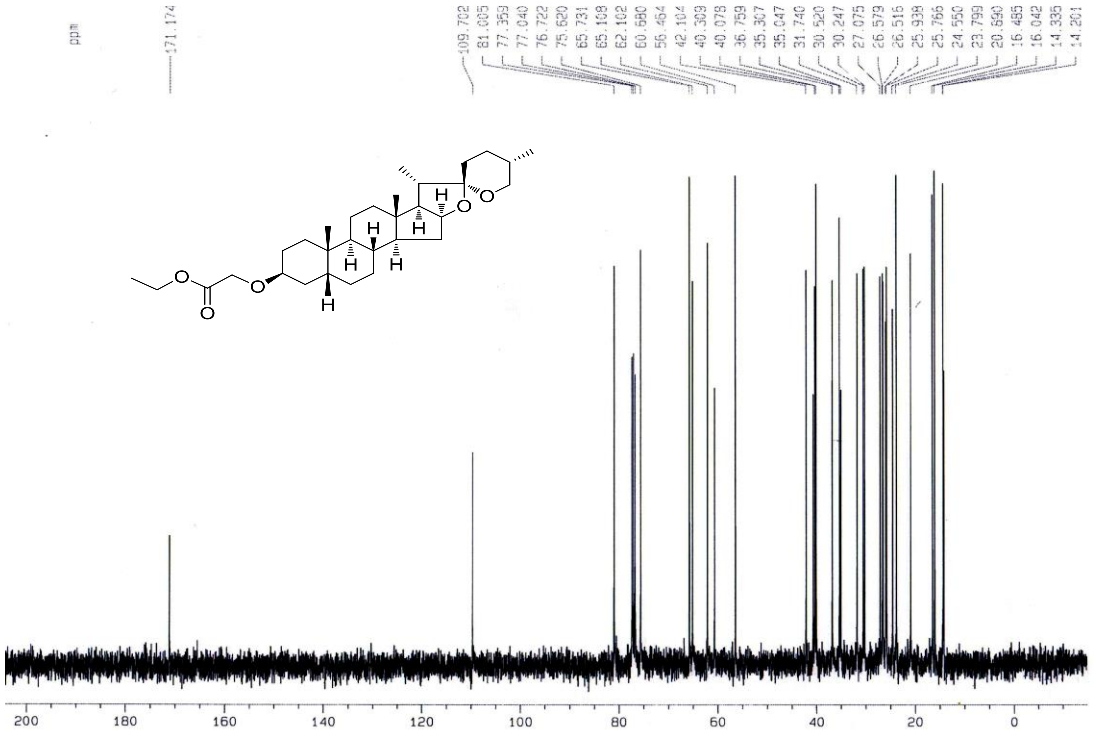

^{1}H NMR of Ethereal SSG (**29**)

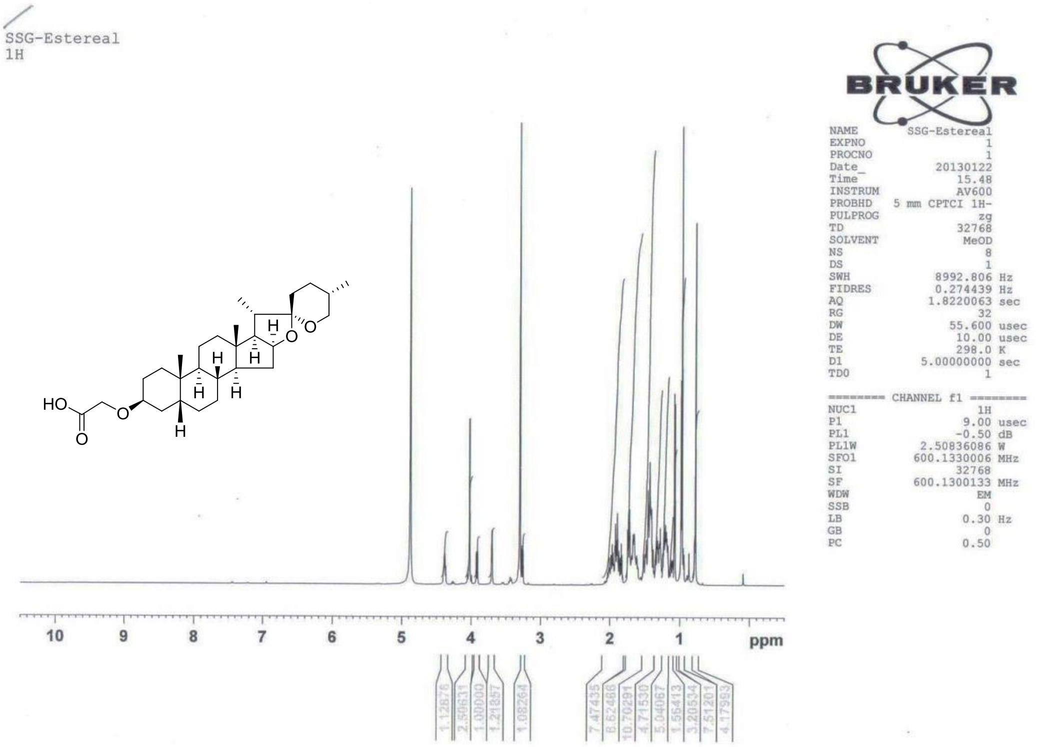

^{13}C NMR of Ethereal SSG (**29**)

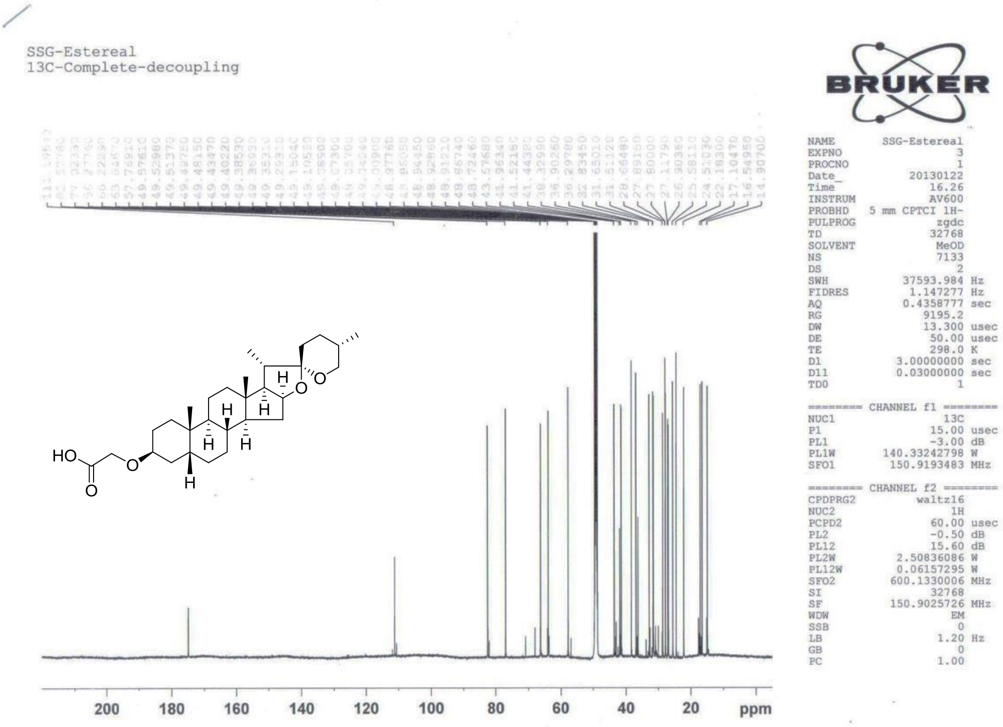

^{1}H NMR of Compound **30**

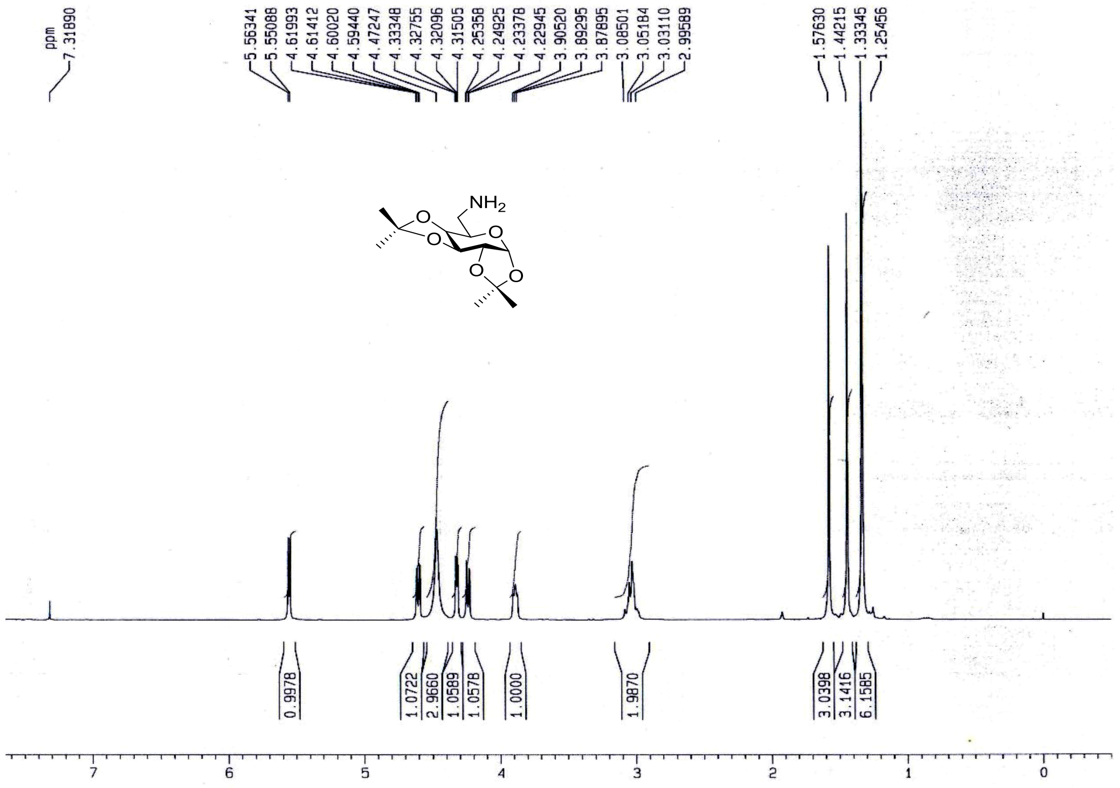

^{13}C NMR of Compound **30**

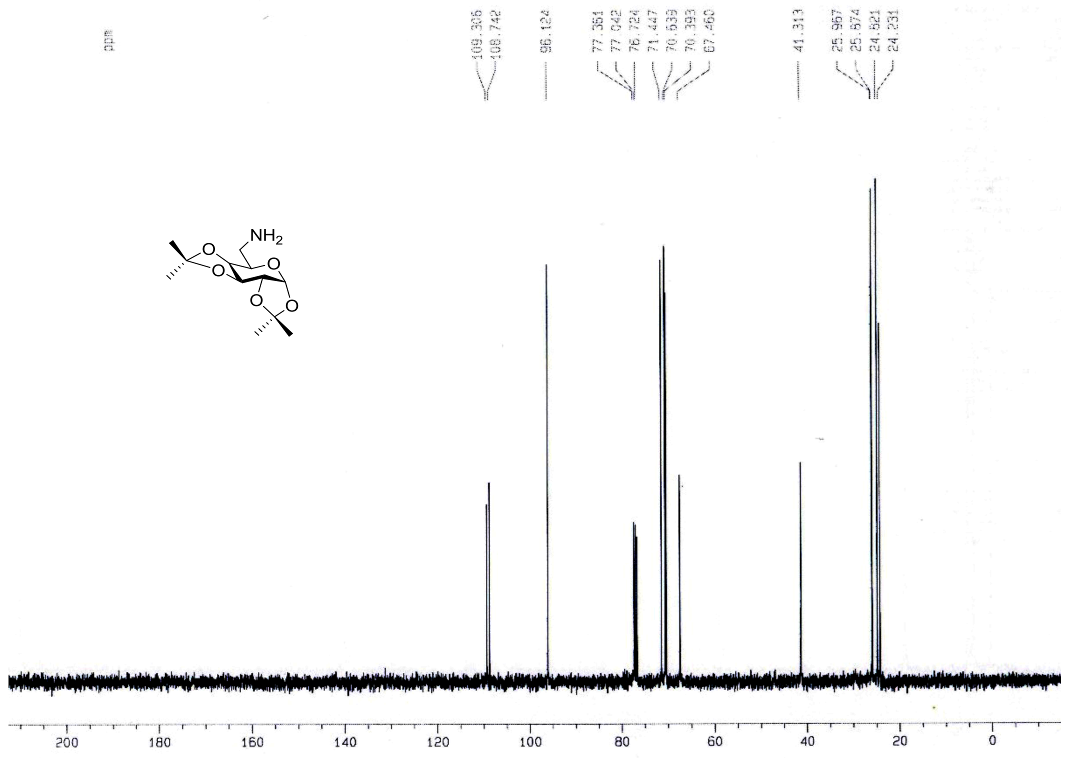

^{1}H NMR of Amide SSG (**31**)

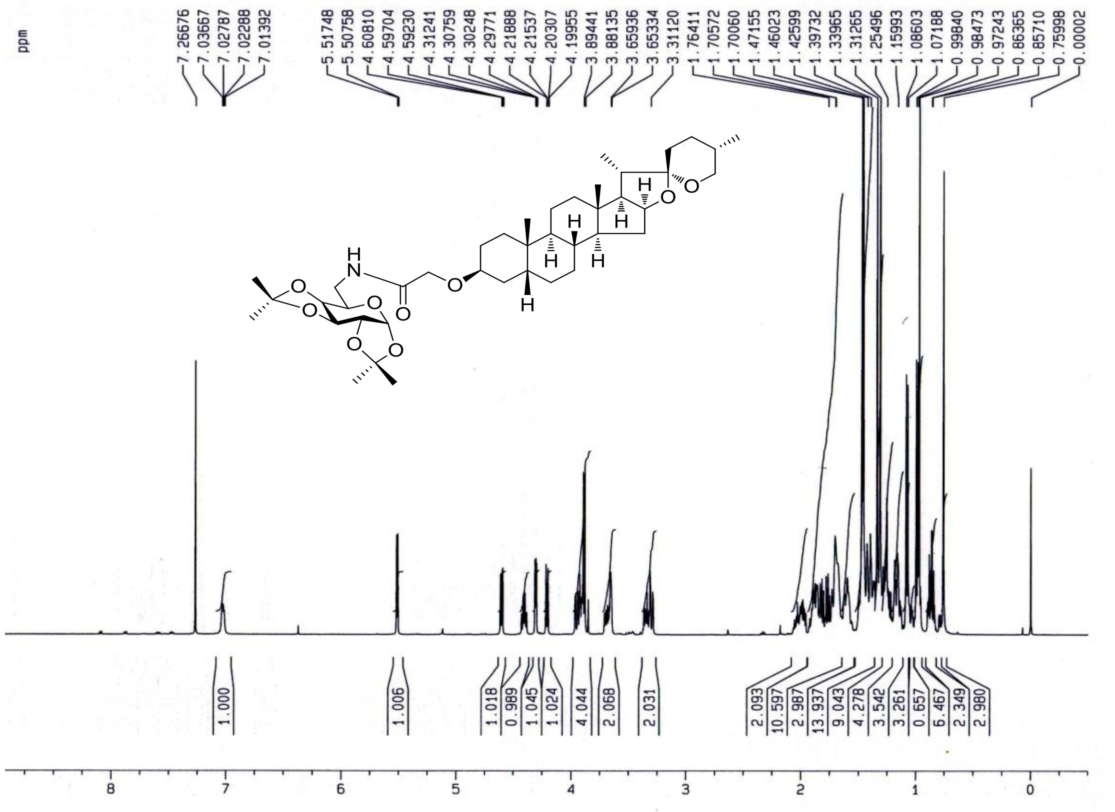

^{13}C NMR of Amide SSG (**31**)

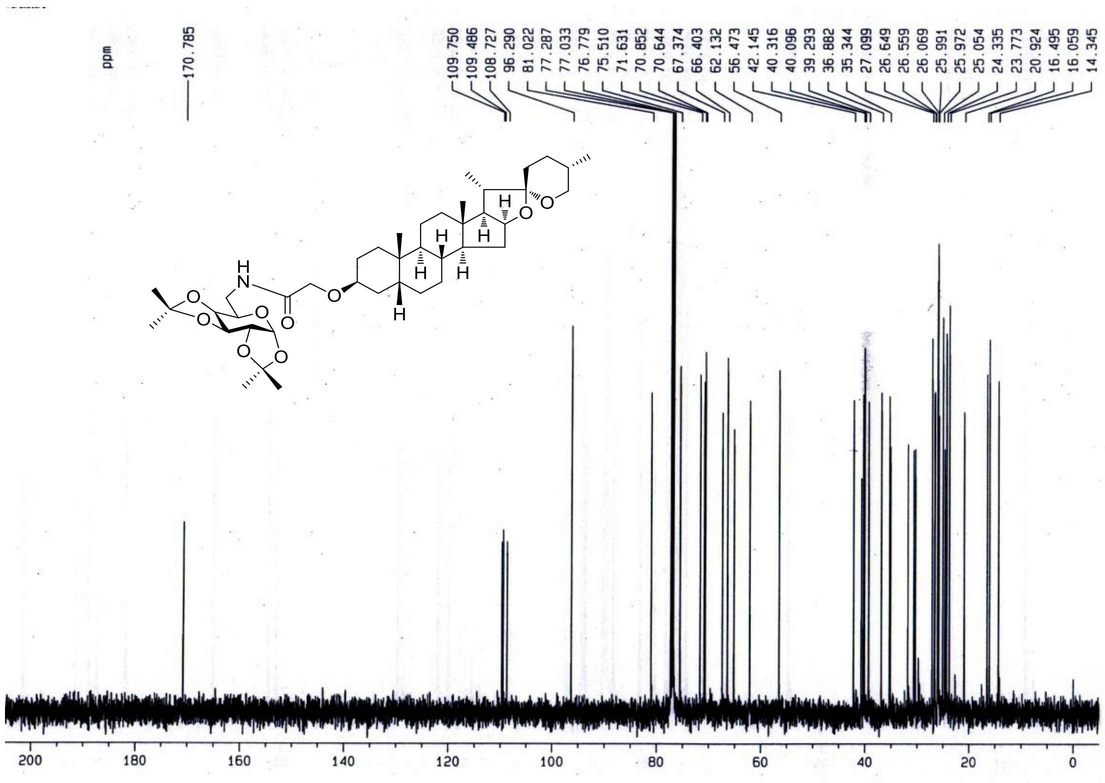

^{1}H NMR of α-OMe SSG (**32**)

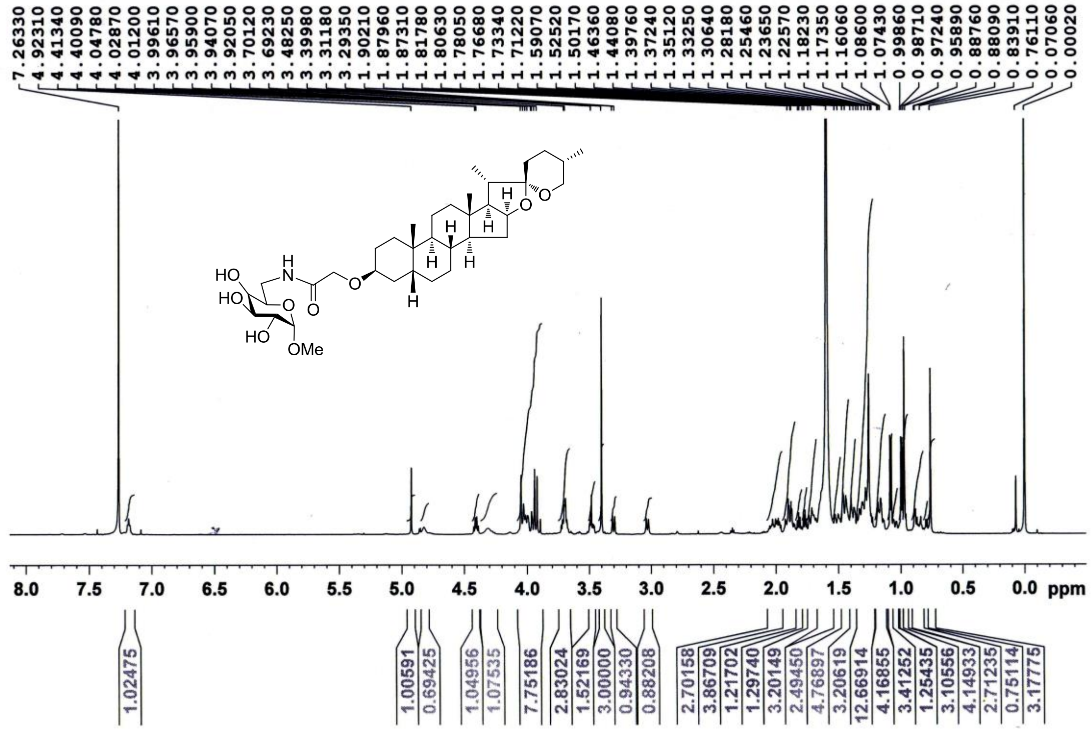

^{13}C NMR of α-OMe SSG (**32**)

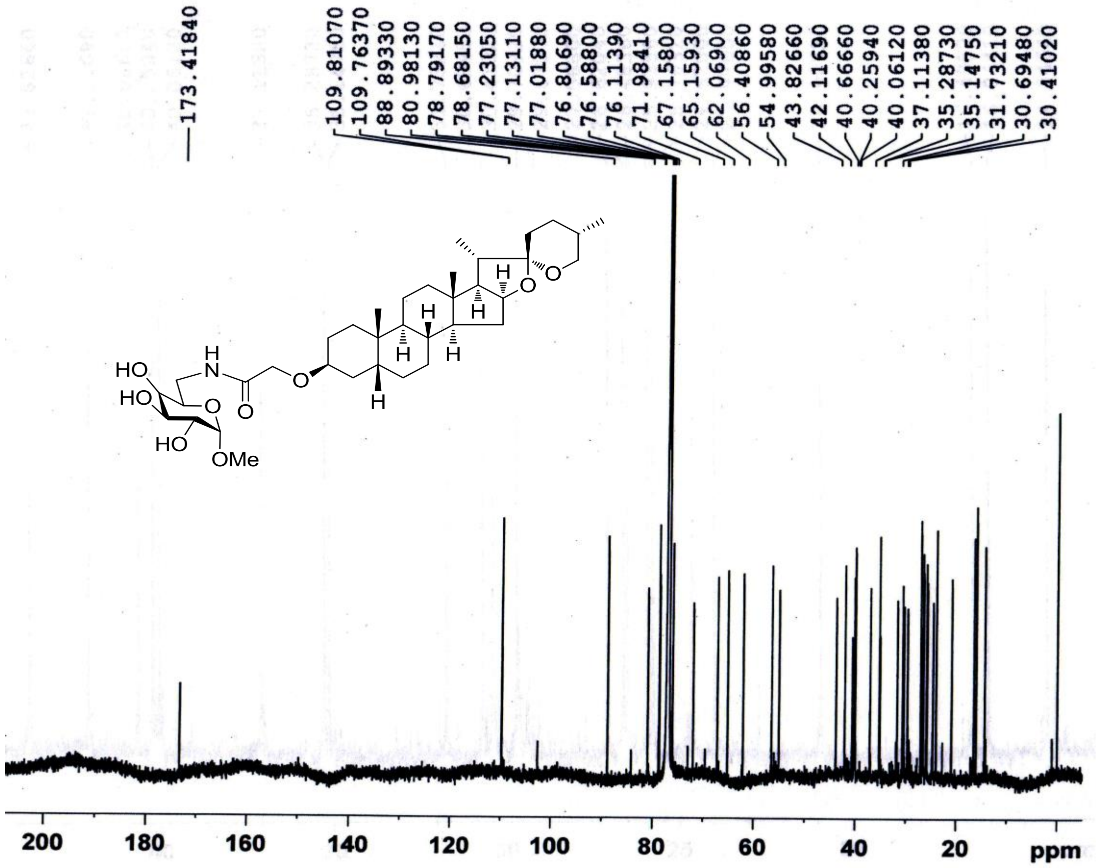

^{1}H NMR of β-OMe SSG (**33**)

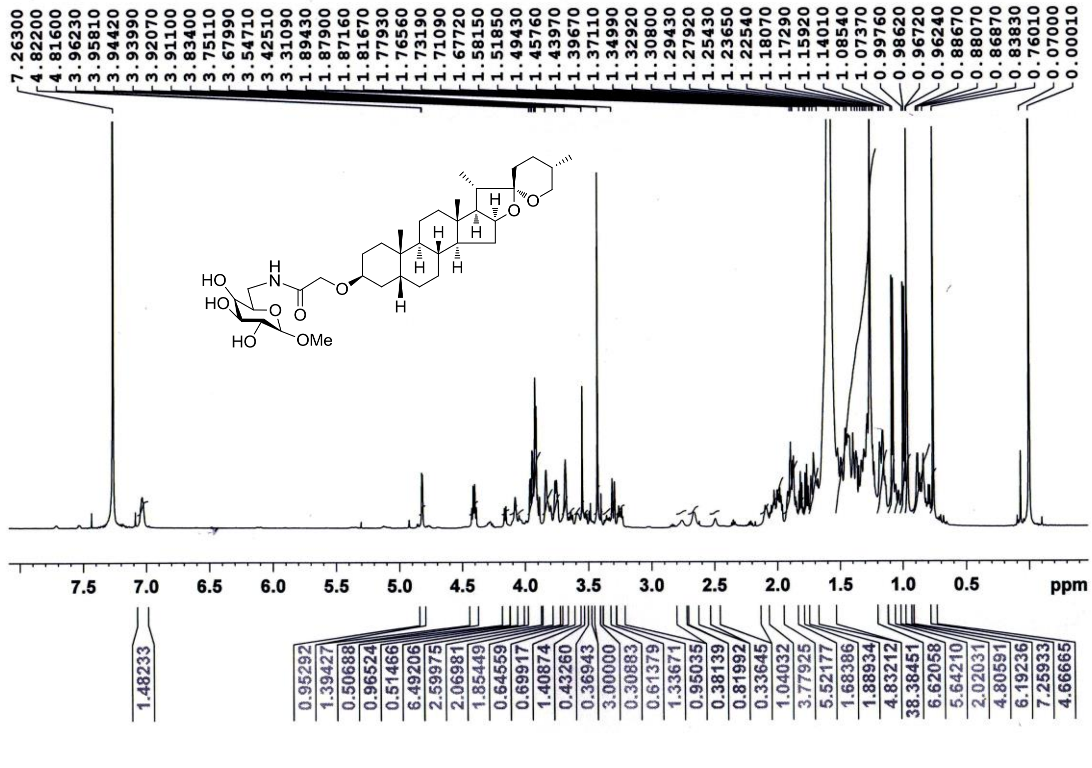

^{13}C NMR of β-OMe SSG (**33**)

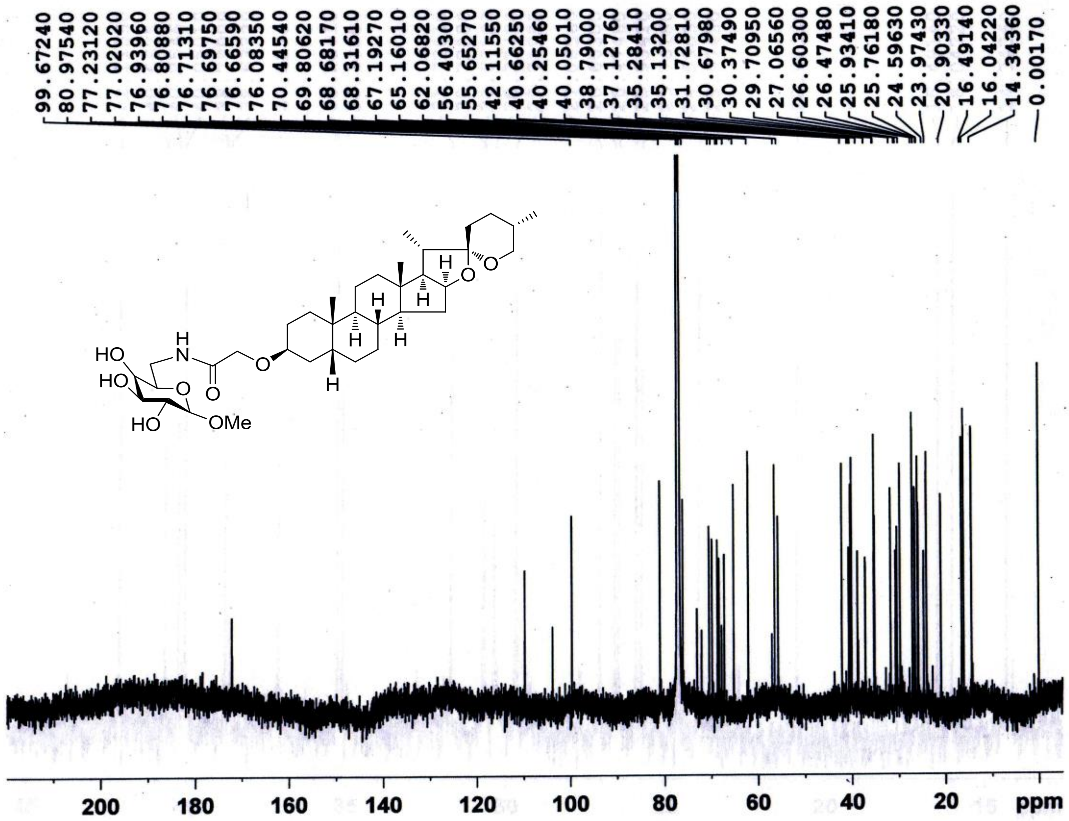

^{1}H NMR of α-,β-OH SSG (**34**)

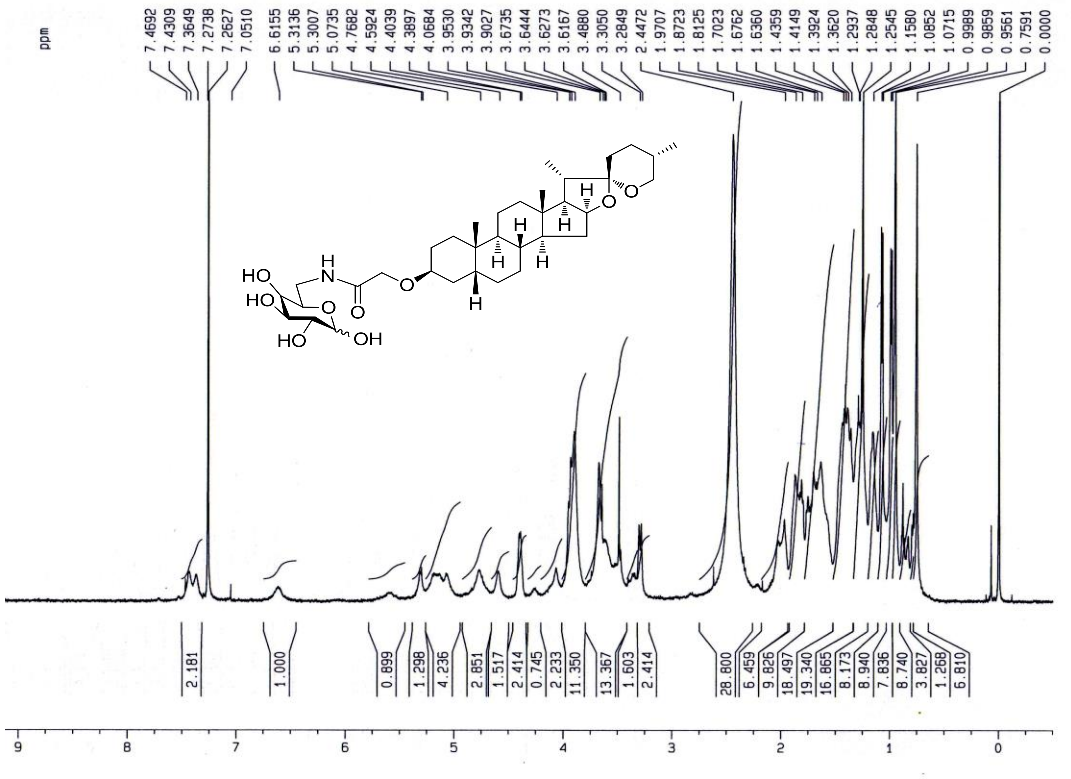

^{13}C NMR of α-,β-OH SSG (**34**)

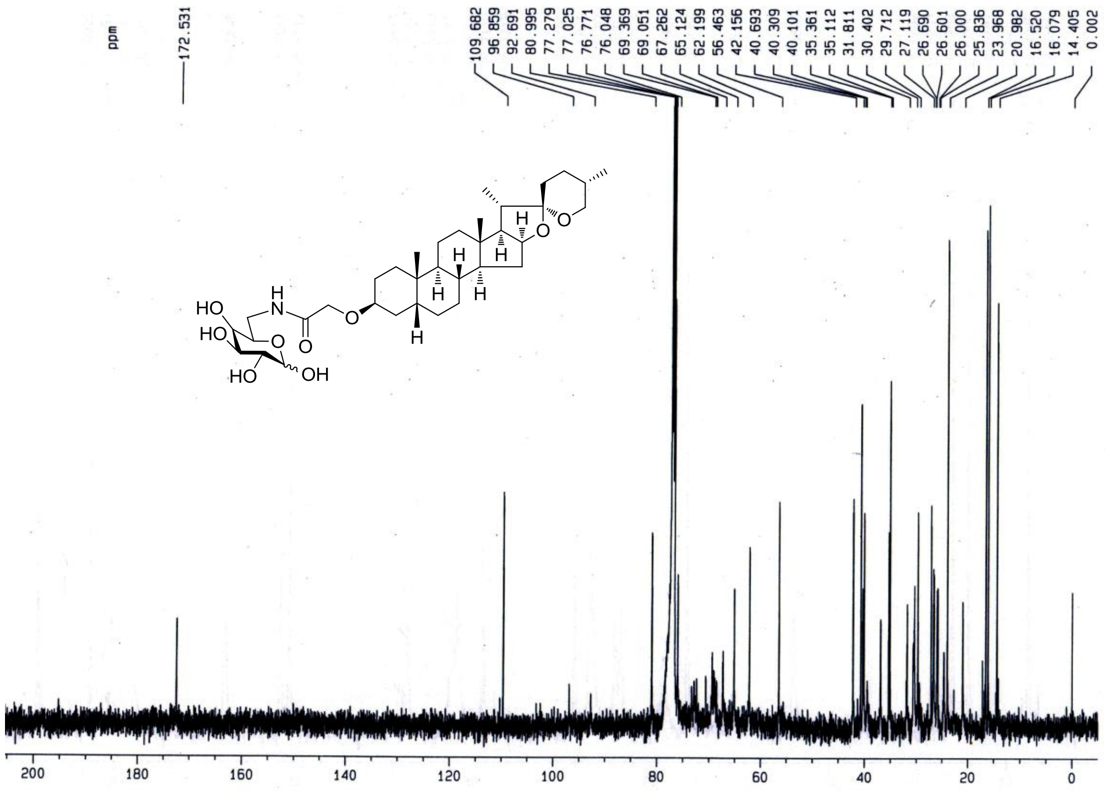

Supplement: Supplementary file 1 [file SC-007-C5SC02377G-s001.pdf]
