# Supplementary material for: Identification of “sarsasapogenin-aglyconed” timosaponins as novel Aβ-lowering modulators of amyloid precursor protein processing
Source: Chem Sci. 2016 Jan 22;7(5):3206–14. doi: 10.1039/c5sc02377g (PMC6005341; doi:10.1039/c5sc02377g)
Supplement: Supplementary file 2 [file SC-007-C5SC02377G-s002.pdf]

Electronic Supplementary Material (ESI) for Chemical Science.
This journal is © The Royal Society of Chemistry 2016

Electronic Supplementary Information

Identification of "sarsasapogenin-aglyconed" timosaponins as novel Aβ lowering modulators of amyloid precursor protein (APP) processing

Table of contents

A. Materials and methods S2

B. Determination of timosaponin levels in mice plasma and brain by ultra-high pressure liquid chromatography coupled with time of flight mass spectrometer (UPLC-QToF/MS) S4

C. Preparation and chemical analysis S6

1. Timosaponin A I (TAI, **3**) S6
2. Timosaponin A III (TAIII, **2**) and Timosaponin A V (TAV, **4**) S8
3. Asparagoside A (AA, **5**) S11
4. Capsicoside A_3 (**8**) S12
5. Dihydrodioscin (**11**) and dihydropolyphyllin D (**12**) S14
6. Tigogenin (**13**) and Neotigogenin (**14**) S15
7. 5α-H, 6α-OH and 5β-H, 6β-OH of diosgenin and yamogenin (**15-18**) S16
8. Sarsasapogenone (**19**) and episarsasapogenin (**20**) S19
9. Dihydrosarsasapogenin (dSSG, **21**) S20
10. Propargyl SSG **25,** triazole SSG **27** and α-OMe triazole SSG **28** S21
11. Ethereal SSG (**29**) S23
12. α-, β-OMe SSG (**32**, **33**) and α-/β-OH SSG (**34**) S24

D. Tables and figures

Table S1 Some Aβ lowering compounds from natural products S27

Table S2 Plasma and brain concentrations of timosaponins in mice (n = 6) treated with three repeated oral doses (100 mg/kg) in two days. S29

Fig. S14 Molecular docking of SSG, TAI, TAIII and cholesterol to the transmembrane domain of APP. S30

E. References S31

A. **Materials and methods**

Reagents: All reagents were of analytical grade (purity > 98%) and were purchased from Sigma-Aldrich Chemical Co. unless otherwise specified. SSG (**1**) was purchased from Wako Pure Chemical Industries, Ltd. Diosgenin (**6**), Dioscin (**9**), Polyphyllin D (**10**), Timosaponin BI (**22**) and Timosaponin BII (**23**) were purchased from Chengdu Must Bio-Technology Co. Ltd. Yamogenin (**7**) was bought from Ambinter c/o Greenpharma while Smilagenin (**24**) was bought from Steraloids Inc. Substances dissolved in DMSO as stock solutions. Cell culture medium constituents were purchased from Life Technologies, Inc.

Cell culture: Neuro-2A cells expressing Swedish mutants of APP (N2A-APPswe provided by Prof. Yifan Han, The Hong Kong Polytechnic University)[1] was cultured in minimal essential medium with 2 mM glutamine, 2 mM pyruvate and 10% fetal bovine serum. Cells were seeded at 6-well plate at 1×10^6 cells/mL and grown for two days until confluence. The medium was replaced and the testing compounds were added at indicated concentrations for 18 h.

Primary cortical neurons were prepared from embryonic day 17 Sprague-Dawley rats (Laboratory Animal Unit, The University of Hong Kong). Cerebral cortices were mechanically dissociated in complete medium (MEM with 18 mM glucose; 2 mM L-glutamine; 5% FBS; hormone cocktail, penicillin (50 U/mL), streptomycin (50 μg/mL), and 25 mM β-mercaptoethanol). Cells were seeded onto six-well plate (0.5×10^6 cells/well) pre-coated with poly-L-lysine. Neurons were maintained in reduced serum Neurobasal Medium supplemented with 2% B-27 supplement, L-glutamine (2 mM), penicillin (50 U/mL), streptomycin (50 μg/mL), and 2-mercaptoethanol for 2 days, and then treated with the timosaponin compounds for another 5 days.

Immunoblot analysis: Cells were washed with phosphate buffered saline and lysed with buffer containing 50 mM Tris-Cl, pH 7.4, 150 mM NaCl, 1% Triton X-100 supplemented with protease inhibitors. Equal amount of proteins (30 μg) was resolved by 16.5% Tris-tricine gels and then transferred to nitrocellulose membrane with 0.2 μm pore size. For detection of secreted Aβ, sAPPα and sAPPβ, cells were treated with the timosaponins for 8 h in complete medium which were then replaced with medium containing 0.1% FBS. After further incubation of 16 h, 50 μL of the conditioned medium was mixed with SDS sample buffer and resolved accordingly. The membrane was blocked with Tris-buffered saline containing 0.1% Tween 20 and 3% BSA and then incubated with primary antibodies at 4 ^{o}C overnight, followed with appropriate secondary antibodies for 2 h. The primary antibodies were: APP c-terminal rabbit antibody (1:5000; Merck Bioscience) for detection of full length APP and APP-CTFs, mouse monoclonal 6E10 (Covance) for secreted Aβ and sAPPα, mouse monoclonal 6A1 (IBL) for sAPPβswe, ADAM-10 rabbit antibody (Millipore) and BACE1

rabbit antibody (Millipore). The immunoreactivities were detected using enhanced chemiluminescence reagents (GE Health care).

For examination of notch cleavage by γ-secretase, N2A-APPswe cells were transfected with myc-tagged NotchΔE construct (pSC2 EMV-6MT, provided by Prof. Raphael Kopan, Washington University, St. Louis, MO) using Fugene HD (Roche). Cells were then treated with timosaponins for 18 h and the expression of NotchΔE and NICD were examined by immunoblot analysis using myc-tagged antibodies.

Aβ ELISA: Aβ concentrations in conditioned medium were assayed using ELISA kits for human $A\beta_{40}$, human $A\beta_{42}$ (ultrasensitive) (Invitrogen), human $A\beta_{38}$ (MyBioSource) (in N2A-APPswe cells) or Wako High Sensitive $A\beta_{x\text{-}42}$ ELISA kit (in Rat primary cortical neuron culture). The viability of the N2A-APPswe and Primary cortical neurons were determined by MTT and LDH release assays, respectively. No significant effect of viability of the neuron culture was observed at the conditions by which the neuronal cells were treated with the timosaponins.

β-Secretase assays: Confluent N2A-APPswe cells were harvested and homogenized with 50 mM MES, pH5.5. Protein extracts (10 μg) were incubated with testing compounds or vehicle for 1 h at room temperature. The β-secretase activities were assayed by adding 10 μM fluorogenic β-secretase substrate (Millipore) to the reaction mixtures and the fluorescence (excitation, 380; emission, 460) were measured. β-Secretase assays were also performed using purified BACE1 (Millipore) at 100 ng in a buffer containing 50 mM MES, pH 5.5.

Immunofluorescence examination of neurite outgrowth: Neuro-2A cells were seeded at 1×10^5 per glass bottom dishes. After treatment, cells were fixed with 4% paraformaldyde for 10 min, permeabilized with 0.1% Triton X-100, and then blocked with 1% BSA in PBS. The cells were incubated with monoclonal antibody raised against type III β-tubulin (Sigma) for 1 h followed by Alexa488 conjugated secondary antibody. Neurite morphology was examined under fluorescence microscope.

Molecular Docking: Docking studies of SSG and timosaponins to the APP was performed using the software ICM Pro 3.7v-2a (molsoft). Briefly, the receptor was built from the NMR structure of β-amyloid precursor protein comprising the 46 amino acid residues (PDB code: 2LP1). Missing side chains and hydrogen atoms were added and optimized. The binding sites were assigned across the whole receptor by the grid maps for van der Waals, electrostatic, hydrogen bonding and hydrophobic interactions. During docking experiments, the completely flexible ligands were fit into the rigid receptor of the APP peptide. Binding of cholesterol to the receptor was also performed for the reference. The docking of the ligand to the receptor

was performed at least three times. The lowest binding scores were computed and the conformations of the corresponding binding pose presented.

Quantum mechanics/Molecular mechanics. The binding between transmembrane and substrate was further optimized using the QM/MM approach and NWChem software. The quantum part (QM) included TAIII (total 116 atoms), while the rest of the system was modelled as the molecular mechanics (MM) level. The QM region was treated at the DFT level using the B3LYP method. The 6-31G* Pople basis set was employed for all the atoms. The transmembrane was described with the AMBER parm99 force field. The binding structure was solvated in a cubic box with 6872 classical SPC/E water molecules. Three negative counter ions (Cl^-) were added to neutralize the charges in the system. The structure was optimized by performing the BFGS algorithm for the QM part and the steepest descent algorithm for the MM part. The optimization of these two regions (QM and MM) was alternated until self-consistency was reached.

Animal experiments: Male C57BL/6N mice were housed in environmentally controlled room (temperature: 25 ± 2 ^{o}C, humidity: 50 ± 5%, 12-h light-dark cycle) in Laboratory Animal Unit, The University of Hong Kong and were freely access to food and water. TAIII, TAI SSG and compound **34** were dissolved in 5% Tween 80 and given to the mice by oral gavage at three repeated doses of 100 mg/kg in 48 hours. Mice were administrated with 5% Tween 80 (control group), TAIII, TAI SSG and **34**. The experimental procedures were performed with the approval of Committee of Use of Live Animal for Teaching and Research of The University of Hong Kong. Blood (about 600-800 µL) was collected by cardiac puncture and added with 1/10 volume of Na_2EDTA (20 mg/mL) to prevent coagulation. After centrifugation at 3000 g for 15 min at 4 ^{o}C, plasma was transferred to a centrifuge tube. The brain was dissected into two hemispheres on ice, weighed and frozen in liquid nitrogen immediately. The left brain was homogenized with 1 mL of buffer containing 5 M guanidine HCl, 50 mM Tris HCl, pH 8.0, with protease inhibitor cocktail and AEBSF using a Polytron and the homogenates were allowed to stand at room temperature for 3h. Both plasma and brain samples were stored at -80 ^{o}C until they were analyzed. The $A\beta_{42}$ levels were determined by Wako High Sensitive $A\beta_{x\text{-}42}$ ELISA kit.

B. Determination of timosaponin levels in mice plasma and brain by ultra-high pressure liquid chromatography tandem mass spectrometry (UPLC-ESI-QTOF-MS/MS)

The UPLC-ESI-QTOF consists of a Waters Q-TOF Premier^{TM} mass spectrometer (Micromass MS Technologies, Manchester, UK) operated in positive ion mode and a Waters ACQUITY ^{TM} UPLC system. The mass spectrometer conditions were optimized as follows: source

temperature, 120 ^{o}C; desolvation temperature, 500 ^{o}C; nebulization gas flow rate, 1000 L/h; cone gas flow rate, 20 L/h; capillary voltage, 2.5 kV; sampling cone voltage, 28V. The data were collected into two separate data channels with the instrument spending 0.5 s on data acquisition for each channel and a 0.1 s inter-channel delay. The mass spectrometer was calibrated by using the solution of sodium formate (0.5 mM) and the TOF scan range was from 100 to 1000 Da. In order to improve mass measurement accuracy a lock mass compound (50 pg µL^{-1} solution of leucine encephalin; m/z 556.2771) was continuously infused at a 0.5 mL min^{-1} using the built-in syringe pump of the instrument.

UPLC condition

Sample was loaded into a Waters ACQUITY^{TM} BEH C_{18} column (100 × 2.1 mm i.d., 1.7 µM) connected to a Waters ACQUITY^{TM} BEH C_{18} guard column (5 × 2.1 mm i.d., 1.7 µM). The gradient elution system consisted of 0.1% formic acid in water (A) and 0.1% formic acid in acetonitrile (B). Separation was achieved by using: 30%-55% B from 0 to 3 min; 55-100% B from 3 to 6 min; 100% B from 6 to 12 min and returned to initial conditions and equilibrated for 3 minutes. The flow rate was 0.3 mL/min and the injection volume was 5 µL.

Standard preparation and sample preparation

Stock solution (10 µM) of each analyte was prepared by dissolving in methanol. They were subject to serial dilution to give calibration curves of SSG **1** (plasma: y = 8.7485× - 0.0676; brain: y = 12.018× - 0.8022), TAIII **2** (plasma: y = 3.1183× - 0.0621; brain: y = 3.1463× + 0.0062), TAI **3** (plasma: y = 14.336× + 0.1312; brain: y = 14.875× - 0.0973) and compound **34** (plasma: y = 34.731× - 0.0248; brain: y = 7.055× - 0.0981). A volume of 200 µL from each concentration of each analyte was transferred to a centrifuge tube and evaporated to dryness by a concentrator. Dioscin (20 µL, 10 µM) was added as an internal standard followed by methanol (130 µL) and control plasma (50 µL). The mixture was vortexed for 5 s and centrifuged (15000 × g) for 15 min. The supernatant was collected and evaporated to dryness by a concentrator. Methanol (100 µL) was added and then vortexed again for 10 s and centrifuged (15000 × g) for 20 min. The supernatant (5 µL) was subject to LC-MS/MS analysis. Analyte-treated plasma, control brain and analyte-treated brain were prepared in a similar manner.

Data acquisition

The acquisition and processing of data were performed by Masslynx (version 4.1) software. Mass window of ±10 mDa and mass resolution of 9000 were set for both molecular ions and product ions. MS full scan was applied to acquire high abundant molecular ions $[M+H]^+$ with accurate mass of **1** (*m/z* 417.3366, Δ = -0.7 ppm), **2** (*m/z* 741.4424, Δ = -0.1 ppm), **3** (*m/z* 579.3887, Δ = 1.7 ppm) and **34** (*m/z* 636.4113, Δ = 0.2 ppm). Thereafter, the collision-induced

dissociation was adjusted to achieve two most intense product ions for each molecular ion in the product scan for quantification (MS/MS). The high abundance of product ions was strongly preferred to enhance the sensitivity of quantification and improve the detection limits. The transitions *m/z* 417.3366 → *m/z* 255.2193 and *m/z* 273.2202 for **1**; *m/z* 741.4424 → *m/z* 255.2195 and *m/z* 273.2204 for **2**; *m/z* 579.3887 → *m/z* 255.2133 and *m/z* 273.2205 for **3** and *m/z* 636.4113 → *m/z* 220.0724 and *m/z* 255.2132 for **34** were monitored. Product ions of each analyte were monitored as a sum for quantification. The data are presented as mean ± S.D.

C. Preparation and chemical analysis

NMR spectra were recorded on a Bruker AV 400 or DRX 500 or AV 600 spectrometers. Each final product was recorded for 1-dimensional (^{1}H NMR, ^{13}C NMR and might with Dept-135) and maybe also 2-dimensional (^{1}H-^{1}H COSY, ^{1}H-^{1}H NOESY, ^{1}H-^{13}C HSQC and ^{1}H-^{13}C HMBC) NMR spectra. Samples were dissolved in chloroform-d_1 or methanol-d_4 or pyridine-d_5 and chemical shifts are expressed in ppm relative to TMS as internal standard reference at ambient temperature except specified. All the NMR spectra were provided in Appendix I. Mass spectra were obtained in EI mode or FAB mode using Thermo Scientific DFS High Resolution Magnetic Sector or ESI using Thermo Scientific LCQ classic or LC-ESI-QTOF HR mass spectrometer (Waters Q-TOF Premier^{TM} mass spectrometer, Micromass MS Technologies). Optical rotation was recorded by ADP440+ polarimeter (Bellingham and Stanley Ltd.). Balance of Sartorius MSE2.7S was used.

1. Preparation of Timosaponin A I (TAI, 3)

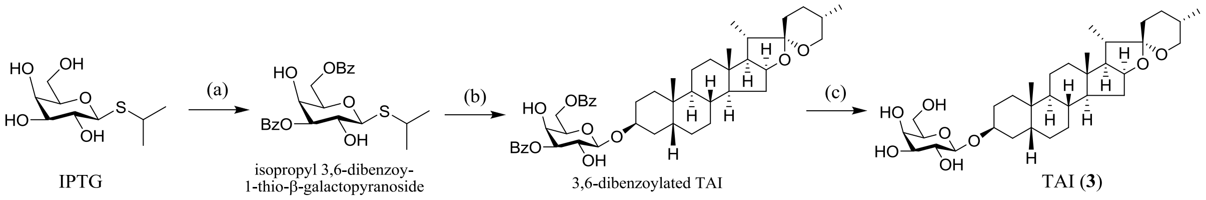

Fig. S1: Synthesis of TAI (**3**).

(a) Isopropyl 3,6-di-*O*-benzoyl-1-thio-β-D-galactopyranoside was synthesized according to literature.[2] Isopropyl-β-D-thiogalactopyranoside (IPTG, 1.015 g, 4.2 mmol) dissolved in anhydrous pyridine (7 mL) was added slowly with benzoyl chloride (0.982 mL, 8.5 mmol). The reaction mixture was stirred at 0 ^{o}C until all the IPTG was consumed and monitored by TLC. The reaction mixture was diluted with CH_2Cl_2 and then washed sequentially with dilute HCl, saturated $NaHCO_3$ and brine. The organic layer was dried over anhydrous Na_2SO_4 and concentrated by vacuum. The crude product was subjected to column chromatography using CH_2Cl_2:EtOAc (25:1) as eluent to obtain isopropyl

3,6-di-*O*-benzoyl-1-thio-β-D-galactopyranoside (0.98 g, yield 52%). ^{1}H NMR (400 MHz, $CDCl_3$) δ (ppm): 8.10 (dd, *J* = 8.4 Hz, 1.3 Hz, 2H), 8.02 (dd, *J* = 8.4 Hz, 1.3 Hz, 2H), 7.55-7.60 (m, 2H), 7.42-7.47 (m, 4H), 5.18 (dd, *J* = 9.6 Hz, 3.2 Hz, 1H), 4.51-4.65 (m, 3H), 4.26-4.28 (m, 1H), 3.99-4.07 (m, 2H), 3.25 (sep, *J* = 6.8 Hz, 1H), 2.52-2.54 (m, 2H), 1.36 (d, *J* = 6.8 Hz, 3H), 1.34 (d, *J* = 6.8 Hz, 3H). ^{13}C NMR (75 MHz, $CDCl_3$) δ (ppm): 166.9, 166.5, 133.9, 133.7, 130.3, 130.1, 129.93, 129.89, 128.9, 128.8, 87.0, 76.9, 76.5, 68.4, 68.0, 63.6, 36.6, 24.6, 24.2. ESI-MS (+ve): *m/z* 469.1 $[M+Na]^+$.

(b) 3,6-Dibenzoylated Timosaponin A I was synthesized according to literature.[3,4] To a mixture of isopropyl 3,6-di-*O*-benzoyl-1-thio-β-D-galactopyranoside (368 mg, 0.824 mmol) and sarsasapogenin (**1**, SSG, 286 mg, 0.687 mmol) in anhydrous CH_2Cl_2 (10 mL), *N*-iodosuccinimide (NIS, 277 mg, 1.237 mmol) and trimethylsilyl trifluoromethanesulfonate (TMSOTf, 15 µL, 0.082 mmol) were added under N_2 atmosphere at -42 ^{o}C. The mixture was stirred at this condition for 1 h, then neutralized with TEA and concentrated. The crude product was subjected to column chromatography using CH_2Cl_2:MeOH (50:1) as eluent to obtain the 3,6-dibenzoylated Timosaponin A I (265 mg, yield 49%). ^{1}H NMR (400 MHz, $CDCl_3$) δ (ppm): 8.10 (dd, *J* = 8.4 Hz, 1.2 Hz, 2H), 8.03 (dd, *J* = 8.4 Hz, 1.2 Hz, 2H), 7.55-7.61 (m, 2H), 7.43-7.48 (m, 4H), 5.17 (dd, *J* = 10.1 Hz, 3.3 Hz, 1H), 4.53-4.64 (m, 2H), 4.47 (d, *J* = 7.7 Hz, 1H), 4.38-4.43 (m, 1H), 4.21-4.23 (m, 1H), 4.02-4.07 (m, 2H), 3.94-3.97 (m, 2H), 3.30 (d, *J* = 11.0 Hz, 1H), 2.37 (d, *J* = 5.6 Hz, 1H), 2.27 (d, *J* = 1.6 Hz, 1H), 1.70-2.03 (m, 10H), 1.28-1.54 (m, 10H), 1.05-1.23 (m, 5H), 1.08 (d, *J* = 7.1 Hz, 3H), 0.99 (d, *J* = 6.8 Hz, 3H), 0.95 (s, 3H), 0.76 (s, 3H). ^{13}C NMR (125 MHz, $CDCl_3$) δ (ppm): 166.4, 166.0, 133.4, 133.3, 129.9, 129.7, 129.63, 129.59, 128.49, 128.45, 109.7, 101.9, 81.0, 75.4, 75.3, 72.3, 69.6, 67.4, 65.2, 62.6, 62.1, 56.4, 42.1, 40.7, 40.3, 40.2, 37.2, 35.3, 35.0, 31.8, 30.4, 30.3, 27.1, 26.64, 26.55, 26.4, 26.0, 25.8, 23.9, 20.9, 16.5, 16.0, 14.3. ESI-MS (+ve): *m/z* 787.4 $[M+H]^+$.

(c) 3,6-Dibenzoylated Timosaponin A I (100 mg, 0.127 mmol) dissolved in anhydrous CH_2Cl_2:MeOH (2:1, 21 mL) was added dropwise with NaOMe in MeOH (0.1 mL, 1.0 M) at room temperature. After stirring for 3.5 h, TLC (CH_2Cl_2:MeOH = 9:1) indicated that all starting materials were consumed. The reaction mixture was neutralized with Amberlite IR 120 (H^+), filtered and concentrated. The crude product was subjected to column chromatography using CH_2Cl_2:MeOH (9:1) to obtain Timosaponin A I (**3**) (66 mg, yield 90%).[3] $[\alpha]_D^{25}$ -58 (*c* 0.42, 3:1 CH_2Cl_2/CH_3OH). NMR was recorded at 50 °C. ^{1}H NMR (500 MHz, MeOH-d_4) δ (ppm): 4.38-4.43 (m, 1H, H-16), 4.28 (d, *J* = 7.5 Hz, 1H, H-1'), 4.14 (br s, Gal-O<u>H</u>), 4.05 (br s, 1H, H-3), 3.94 (dd, *J* = 11.0 Hz, 2.5 Hz, 1H, H-26a), 3.85 (d, *J* = 2.7 Hz, 1H, H-4'), 3.73 (d, *J* = 6.0 Hz, 2H, H-6'), 3.49 (m, 3H, H-2', H-3', H-5'), 3.27 (br d, *J* = 11.0 Hz, 1H, H-26b), 1.60-2.06 (m, 12H), 1.11-1.57 (m, 16H), 1.08 (d, *J* = 7.1 Hz, 3H, H-27), 0.99 (d, *J* = 8.0 Hz, 3H, H-21), 0.98 (s, 3H, H-19), 0.79 (s, 3H, H-18). ^{13}C NMR (125 MHz, MeOH-d_4) δ (ppm): 111.2 (C-22), 103.7 (C-1'), 82.6 (C-16), 76.7

(C-5'), 76.1 (C-3), 75.5 (C-3'), 73.0 (C-2'), 70.6 (C-4'), 66.3 (C-26), 64.0 (C-17), 62.7 (C-6'), 57.9 (C-14), 43.7 (C-20), 42.0 (C-13), 41.7 (C-9), 41.6 (C-12), 38.2 (C-5), 37.0 (C-8), 36.3 (C-10), 32.9 (C-15), 31.7 (C-4), 31.5 (C-1), 28.7 (C-25), 27.9 (C-6, C-7), 27.7 (C-2), 27.3 (C-23), 26.9 (C-24), 24.3 (C-19), 22.2 (C-11), 17.0 (C-18), 16.5 (C-27), 14.7 (C-21). ESI-HRMS calcd for $C_{33}H_{55}O_8^+$: 579.3897 $[M+H]^+$; found: 579.3901.

2. Preparation of Timosaponin A III (TAIII, 2) and Timosaponin A V (TAV, 4).

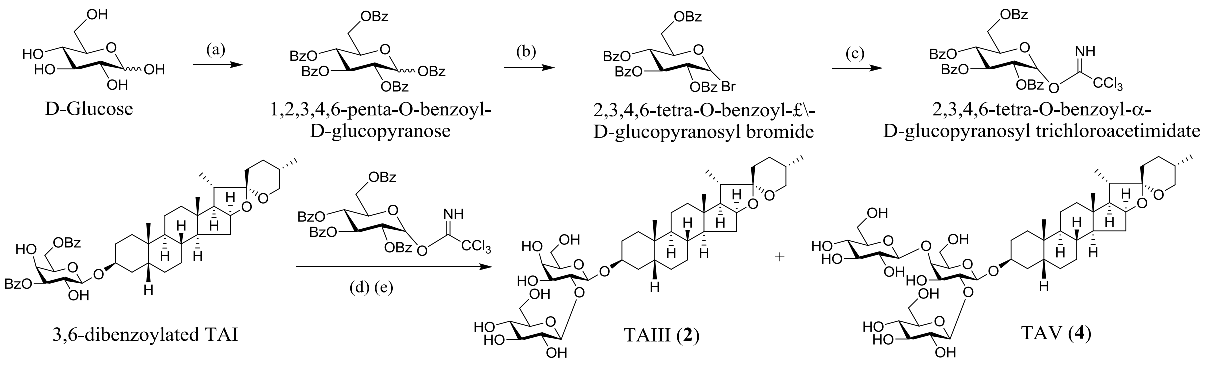

Fig. S2: Synthesis of TAIII (**2**) and TAV (**4**).

Preparation of 1α/β,2,3,4,6-penta-*O*-benzoyl-D-glucopyranose, 2,3,4,6-tetra-*O*-benzoyl-α-D-glucopyranosyl bromide and 2,3,4,6-tetra-*O*-benzoyl-α-D-glucopyranosyl trichloro -acetimidate was made with reference.[5]

(a) To a solution of D-glucose (1.8 g, 10.0 mmol) in pyridine (10 mL) was added benzoyl chloride (6.2 mL, 53 mmol) and DMAP (~5 mg) as a catalyst. After stirring overnight at room temperature, the reaction mixture was quenched with H_2O (30 mL), extracted with CH_2Cl_2, dried with anhydrous Na_2SO_4, filtered and concentrated. The crude product was subjected to column chromatography using n-hexane:EtOAc (2:1) as eluent to obtain the mixed products of 1α,2,3,4,6-penta-*O*-benzoyl-D-glucopyranose and 1β,2,3,4,6-penta-*O*-benzoyl-D-glucopyranose (7.0 g, yield 85%). ^1H NMR (400 MHz, $CDCl_3$) δ (ppm): 7.86-8.14 (m, 10H), 7.26-7.55 (m, 15H), 6.05-6.89 (m, 2H), 5.70-5.92 (m, 2H), 4.41-4.70 (m, 3H). ESI-MS (+ve): *m/z* 718.2 $[M+NH_4]^+$.

(b) To a solution of 1,2,3,4,6-penta-*O*-benzoyl-D-glucopyranose (2.5 g, 3.57 mmol) in anhydrous CH_2Cl_2 at 0 ^oC was added HBr solution in AcOH (33%, 10 mL) and stirred for 1 h. The temperature was then increased to room temperature and stirred for another hour. The solvent was removed under vacuum and the residue was dissolved in CH_2Cl_2 (100 mL) and neutralized with saturated aqueous $NaHCO_3$ (50 mL). The organic layer was separated and washed with H_2O (3 × 50 mL), saturated aqueous $NaHCO_3$ (3 × 30 mL) and brine (2 × 30 mL). Then the solution was dried with anhydrous Na_2SO_4 and filtered. Concentrated in vacuum to afford a white foamy solid (quantitative yield) 2,3,4,6-tetra-*O*-benzoyl-α-D-glucopyranosyl bromide. ^1H NMR (400 MHz, $CDCl_3$) δ

(ppm): 8.07 (dd, J = 8.5 Hz, 1.4 Hz, 2H), 8.00 (dd, J = 8.5 Hz, 1.3 Hz, 2H), 7.96 (dd, J = 8.5 Hz, 1.3 Hz, 2H), 7.88 (dd, J = 8.5 Hz, 1.3 Hz, 2H), 7.27-7.59 (m, 12H), 6.88 (d, J = 4.0 Hz, 1H), 6.28 (t, J = 9.8 Hz, 1H), 5.84 (t, J = 10.0 Hz, 1H), 5.34 (dd, J = 10.0 Hz, 4.0 Hz, 1H), 4.72-4.77 (m, 1H), 4.68 (dd, J = 12.6 Hz, 2.6 Hz, 1H), 4.52 (dd, J = 12.5 Hz, 4.5 Hz, 1H). ^{13}C NMR (100 MHz, $CDCl_3$) δ (ppm): 166.0, 165.6, 165.3, 165.1, 133.8, 133.7, 133.4, 133.3, 130.1, 129.9, 129.84, 129.76, 129.5, 128.8, 128.57, 128.55, 128.51, 128.47, 128.40, 128.38, 86.9, 72.7, 71.5, 70.7, 68.0, 62.0. ESI-MS (+ve): *m/z* 681.0 $[M+Na]^+$.

(c) A solution of the crude 2,3,4,6-tetra-*O*-benzoyl-α-D-glucopyranosyl bromide (1.0 g, 1.52 mmol) in a mixture of acetone and H_2O (6:1, 7 mL) was added with Ag_2CO_3 (1.3 g, 4.72 mmol) and stirred at room temperature for 4 h. The mixture was filtered through celite and the filtrate was concentrated. A solution of the resulting hemiacetal and Cl_3CCN (1.05 mL, 10.5 mmol) in anhydrous CH_2Cl_2 (5 mL) at 0 ^oC was added with 1,8-diazabicyclo[5.4.0]undec-7-ene (DBU) (0.27 mL, 1.8 mmol) under N_2 and stirred at 0 ^oC for 3 h. The mixture was concentrated and subjected to column chromatography using n-hexane:EtOAc:CH_2Cl_2 (10:1:2) as eluent to afford the 2,3,4,6-tetra-*O*-benzoyl-α-D-glucopyranosyl trichloroacetimidate (828 mg, yield 75%). ^{1}H NMR (400 MHz, $CDCl_3$) δ (ppm): 8.64 (s, 1H), 8.04 (d, J = 7.4 Hz, 2H), 7.94-7.97 (m, 4H), 7.87 (d, J = 7.4 Hz, 2H), 7.27-7.57 (m, 12H), 6.85 (d, J = 3.6 Hz, 1H), 6.29 (t, J = 10.0 Hz, 1H), 5.83 (t, J = 9.9 Hz, 1H), 5.63 (dd, J = 10.2 Hz, 3.7 Hz, 1H), 4.64-4.67 (m, 2H), 4.50 (dd, J = 12.6 Hz, 5.3 Hz, 1H). ^{13}C NMR (100 MHz, $CDCl_3$) δ (ppm): 166.1, 165.7, 165.4, 165.2, 160.5, 133.9, 133.6, 133.3, 133.2, 130.1, 129.9, 129.8, 129.74, 129.69, 129.6, 128.85, 128.78, 128.61, 128.55, 128.5, 128.41, 128.37, 93.1, 90.7, 70.7, 70.2, 68.7, 62.5. MS low resolution of 2,3,4,6-tetra-*O*-benzoyl-α-D-glucopyranosyl trichloroacetimidate was hardly detected probably due to the instability under the MS condition.

(d) Synthetic route was adopted according to literature.[6] To a mixture of 3,6-dibenzoylated TAI (65.7 mg, 0.083 mmol), 2,3,4,6-tetra-*O*-benzoyl-α-D-glucopyranosyl trichloroacetimidate (160 mg, 0.184 mmol) and powdered 4Å molecular sieves (100 mg) in anhydrous CH_2Cl_2 (10 mL) at 0 ^oC under N_2 was added TMSOTf (4.5 µL, 0.026 mmol). After stirring at 0 ^oC for 2 h, the reaction was quenched with TEA. The reaction mixture was filtered through celite, filtrate was concentrated and subjected to column chromatography using n-hexane:EtOAc (2:1) as eluent to give the crude *O*-benzoylated product.

(e) A solution of crude *O*-benzoylated product dissolved in anhydrous CH_2Cl_2:MeOH (2:1, 18 mL) was added with NaOMe in MeOH (0.2 mL, 1.0 M). After stirring at room temperature for 2 h, TLC monitoring (CH_2Cl_2:MeOH = 4:1) indicated that all starting material was consumed. The reaction mixture was neutralized with Amberlite IR 120 (H^+), filtered and concentrated. Purified the crude sample by column chromatography using

CH_2Cl_2:MeOH (4:1) as eluent to obtain disaccharide TAIII (**2**) (31 mg, yield 60%) and trisaccharide TAV (**4**) (23 mg, yield 31%).

Timosaponin A V (TAV, **4**): $[\alpha]_D^{25}$ -31 (*c* 0.33, 2:1 CH_2Cl_2/CH_3OH). ^1H NMR (600 MHz, pyridine-d_5): δ (ppm): 5.27 (d, *J* = 7.9 Hz, 1H, H-1''), 5.23 (d, *J* = 7.7 Hz, 1H, H-1'''), 4.85 (d, *J* = 7.7 Hz, 1H, H-1'), 4.71 (d, *J* = 3.1 Hz, 1H, H-4'), 4.58-4.66 (m, 4H, H-16, H-6'a, H-6''a, H-2'), 4.46-4.52 (m, 2H, H-6'''), 4.31-4.35 (m, 2H, H-3', H-4'''), 4.06-4.27 (m, 9H, H-3, H-6'b, H-6''b, H-5'', H-3'', H-2'', H-2''', H-4'', H-26b), 3.97-4.02 (m, 2H, H-5', H-3'''), 3.81-3.84 (m, 1H, H-5'''), 3.38 (d, *J* = 11.0 Hz, 1H, H-26a), 2.13-2.18 (m, 2H, H-5, H-24a), 2.02 (dt, *J* = 6.8 Hz, 5.6 Hz, 1H, H-15a), 1.90-1.95 (m, 3H), 1.80-1.89 (m, 5H), 1.67-1.71 (m, 1H, H-9a), 1.61 (br s, 1H, H-25), 1.20-1.52 (m, 13H), 1.17 (d, *J* = 6.9 Hz, 3H, H-21), 1.02-1.12 (m, 2H), 1.09 (d, *J* = 7.1 Hz, 3H, H-27), 0.99 (s, 3H, H-19), 0.86-0.98 (m, 3H), 0.83 (s, 3H, H-18). ^{13}C NMR (150 MHz, pyridine-d_5) δ (ppm): 109.5 (C-22), 106.6 (C-1''), 105.8 (C-1'''), 102.0 (C-1'), 82.0 (C-2'), 81.1 (C-16), 78.8 (C-4'), 78.4 (C-3''), 78.2 (C-5''), 78.1 (C-3'''), 77.8 (C-5'''), 76.7 (C-2''), 75.7 (C-2'''), 75.4 (C-3), 74.9 (C-5'), 74.7 (C-3'), 72.0 (C-4''), 71.5 (C-4'''), 64.9 (C-26), 62.9 (C-6''), 62.7 (C-17), 62.6 (C-6'''), 60.5 (C-6'), 56.2 (C-14), 42.2 (C-20), 40.6 (C-13), 40.1 (C-12), 40.0 (C-9), 36.7 (C-5), 35.3 (C-8), 35.0 (C-10), 31.9 (C-15), 30.7 (C-4), 30.7 (C-1), 27.3 (C-25), 26.7 (C-2), 26.5 (C-6, C-7), 26.2 (C-23), 26.0 (C-24), 23.8 (C-19), 20.9 (C-11), 16.4 (C-18), 16.1 (C-27), 14.7 (C-21). ESI-HRMS calcd for $C_{45}H_{75}O_{18}^+$: 903.4953 $[M+H]^+$; found: 903.4960.

Timosaponin AIII (TAIII, **2**): $[\alpha]_D^{25}$ -43 (*c* 0.56, 2:1 CH_2Cl_2/CH_3OH). ^1H NMR (500 MHz, pyridine-d_5) δ (ppm): 5.32 (overlay with H_2O peak, 1H, H-1''), 4.94 (d, *J* = 7.6 Hz, 1H, H-1'), 4.70 (dd, *J* = 9.2 Hz, 8.0 Hz, 1H, H-2'), 4.59-4.64 (m, 2H, H-16, H-4'), 4.54 (dd, *J* = 11.6 Hz, 2.6 Hz, 1H, H-6''a), 4.41-4.49 (m, 3H, H-6', H-6''b), 4.36 (s, 1H, H-3), 4.29-4.34 (m, 2H, H-3', H-4''), 4.23 (t, *J* = 9.0 Hz, 1H, H-3''), 4.09-4.12 (m, 2H, H-2'', H-26b), 4.06 (t, *J* = 6.1 Hz, 1H, H-5'), 3.86-3.89 (m, 1H, H-5''), 3.41 (d, *J* = 11.0 Hz, 1H, H-26a), 2.15-2.20 (m, 2H, H-5, H-24a), 1.80-2.07 (m, 9H), 1.70-1.73 (m, 1H, H-12a), 1.62 (br s, 1H, H-25), 1.23-1.54 (m, 10H), 1.19 (d, *J* = 6.9 Hz, 3H, H-21), 1.10 (d, *J* = 6.9 Hz, 3H, H-27), 0.99 (s, 3H, H-19), 0.85 (s, 3H, H-18). ^{13}C NMR (125 MHz, pyridine-d_5) δ (ppm): 110.0 (C-22), 106.1 (C-1''), 102.6 (C-1'), 81.7 (C-2'), 81.6 (C-16), 78.7 (C-5''), 78.3 (C-3''), 77.1 (C-2''), 76.8 (C-5'), 75.7 (C-3), 75.5 (C-3'), 72.0 (C-4''), 70.1 (C-4'), 65.4 (C-26), 63.2 (C-17), 63.1 (C-6''), 62.4 (C-6'), 56.7 (C-14), 42.8 (C-20), 41.2 (C-13), 40.6 (C-12), 40.5 (C-9), 37.1 (C-5), 35.8 (C-8), 35.5 (C-10), 32.4 (C-15), 31.2 (C-1), 31.1 (C-4), 27.8 (C-25), 27.3 (C-2), 27.0 (C-6, C-7), 26.7 (C-23), 26.4 (C-24), 24.3 (C-19), 21.4 (C-11), 16.9 (C-18), 16.5 (C-27), 15.2 (C-21). ESI-HRMS calcd for $C_{39}H_{65}O_{13}^+$: 741.4425 $[M+H]^+$; found: 741.4424.

3. **Preparation of Asparagoside A (AA, 5)**

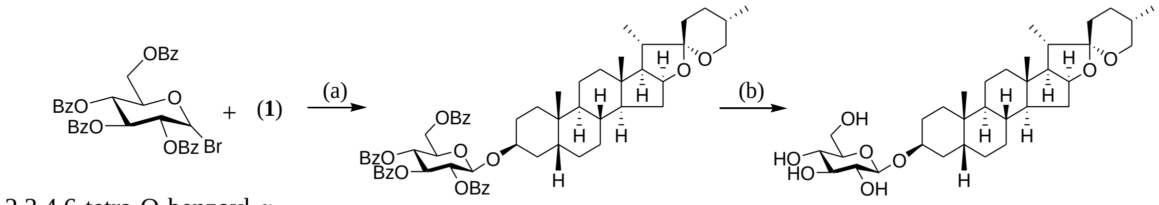

2,3,4,6-tetra-O-benzoyl-α-D-glucopyranosyl bromide — Benzoylated asparagoside A — AA (5)

Fig. S3: Synthesis of AA (**5**).

(a) Synthetic preparation of benzoylated asparagoside A was conducted according to published report.[7,8] To a mixture of SSG (**1**, 208 mg, 0.499 mmol), 2,3,4,6-tetra-*O*-benzoyl-α-D-glucopyranosyl bromide (311 mg, 0.472 mmol) and powdered 4Å molecular sieves (600 mg) in anhydrous CH_2Cl_2 (10 mL) at -42 ^oC under N_2 was added AgOTf (130 mg, 0.5 mmol) in dry toluene (2 mL). The reaction mixture was stirred at -42 ^oC for 3 h and continued stirring overnight at room temperature. The mixture was diluted with CH_2Cl_2 (10 mL), filtered through celite and the filtrate was concentrated. The crude product was then subjected to column chromatography using n-hexane:EtOAc:CH_2Cl_2 (10:1:2) as eluent to give benzoylated asparagoside A (169 mg, yield 36%). ^1H NMR (400 MHz, $CDCl_3$) δ (ppm): 8.00 (dd, J = 8.3 Hz, 1.2 Hz, 2H), 7.96 (dd, J = 8.4 Hz, 1.1 Hz, 2H), 7.89 (dd, J = 8.4 Hz, 1.1 Hz, 2H), 7.85 (dd, J = 8.4 Hz, 1.2 Hz, 2H), 7.28-7.55 (m, 12H), 5.93 (t, J = 9.7 Hz, 1H), 5.67 (t, J = 9.7 Hz, 1H), 5.56 (dd, J = 9.8 Hz, 7.9 Hz, 1H), 4.88 (d, J = 7.9 Hz, 1H), 4.61 (dd, J = 12.0 Hz, 3.2 Hz, 1H), 4.51 (dd, J = 12.1 Hz, 5.3 Hz, 1H), 4.34-4.40 (m, 1H), 4.12-4.16 (m, 1H), 4.05 (br s, 1H), 3.93 (dd, J = 10.8 Hz, 2.4 Hz, 1H), 3.29 (d, J = 10.9 Hz, 1H), 1.65-2.08 (m, 10H), 1.10-1.46 (m, 15H), 1.07 (d, J = 7.0 Hz, 3H), 0.97 (d, J = 6.7 Hz, 3H), 0.69 (s, 3H), 0.55 (s, 3H). ^{13}C NMR (100 MHz, $CDCl_3$) δ (ppm): 166.2, 165.9, 165.2, 164.9, 133.4, 133.21, 133.16, 133.1, 129.8, 129.73, 129.68, 129.6, 129.41, 128.88, 128.86, 128.40, 128.37, 128.33, 128.30, 109.7, 98.9, 81.0, 74.4, 72.9, 72.1, 71.8, 70.1, 65.1, 63.4, 62.1, 56.4, 42.1, 40.6, 40.2, 40.0, 36.2, 35.2, 34.8, 31.7, 30.1, 29.4, 27.1, 26.4, 26.4, 26.1, 25.9, 25.8, 23.3, 20.8, 16.4, 16.0, 14.3. EI-MS (+ve): *m/z* 994.5 $[M]^+$.

(b) Benzoylated asparagoside A (290 mg, 0.291 mmol) was dissolved in anhydrous CH_2Cl_2:MeOH (2:1, 10.5 mL) and then NaOMe in MeOH (0.15 mL, 1.0 M) was added dropwise at room temperature. After stirring for 2 h, TLC (CH_2Cl_2:MeOH = 9:1) indicated that all starting material was consumed. The reaction mixture was neutralized with Amberlite IR 120 (H^+), filtered and concentrated. The crude product was subjected to column chromatography using CH_2Cl_2:MeOH (10:1) as eluent to obtain the product asparagoside A (**5**) (160 mg, yield 95%). $[\alpha]^{25}_{D}$ -61 (*c* 0.79, 2:1 CH_2Cl_2/CH_3OH). ^1H NMR (600 MHz, MeOH-d_4) δ (ppm): 4.37-4.40 (m, 1H, H-16), 4.30 (d, J = 7.8 Hz, 1H,

H-1'), 4.05 (br s, 1H, H-3), 3.92 (dd, J = 10.8 Hz, 2.0 Hz, 1H, H-26a), 3.83 (dd, J = 11.8 Hz, 1.8 Hz, 1H, H-6'a), 3.64 (dd, J = 11.8 Hz, 5.6 Hz, 1H, H-6'b), 3.33 (t, J = 9.0 Hz, 1H, H-3'), 3.26-3.28 (m, 2H, H-26b, H-4'), 3.21-3.24 (m, 1H, H-5'), 3.15-3.18 (m, 1H, H-2'), 1.79-2.04 (m, 8H), 1.73-1.79 (m, 2H), 1.61-1.66 (m, 3H), 1.42-1.54 (m, 6H), 1.11-1.35 (m, 8H), 1.08 (d, J = 7.1 Hz, 3H, H-27), 0.99 (d, J = 6.3 Hz, 3H, H-21), 0.98 (s, 3H, H-19), 0.78 (s, 3H, H-18). ^{13}C NMR (150 MHz, MeOH-d_4) δ (ppm): 111.2 (C-22), 102.8 (C-1'), 82.6 (C-16), 78.4 (C-3'), 78.0 (C-5'), 75.7 (C-3), 75.3 (C-2'), 71.9 (C-4'), 66.2 (C-26), 63.8 (C-17), 62.9 (C-6'), 57.8 (C-14), 43.6 (C-20), 42.0 (C-13), 41.54 (C-9), 41.51 (C-12), 38.0 (C-5), 36.9 (C-8), 36.3 (C-10), 32.8 (C-15), 31.6 (C-4), 31.1 (C-1), 28.7 (C-25), 27.9 (C-6, C-7), 27.6 (C-2), 27.1 (C-23), 26.9 (C-24), 24.4 (C-19), 22.2 (C-11), 17.1 (C-18), 16.5 (C-27), 14.9 (C-21). ESI-HRMS calcd for $C_{33}H_{55}O_8^+$: 579.3897 $[M+H]^+$; found: 579.3898.

4. **Preparation of Capsicoside A$_3$ (8)**

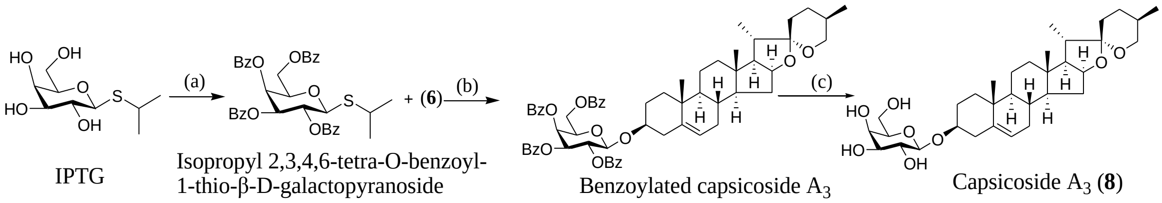

Fig. S4: Synthesis of Capsicoside A$_3$ (**8**).

(a) Isopropyl 2,3,4,6-tetra-*O*-benzoyl-1-thio-β-D-galactopyranoside was synthesized according to literature.[2] To a solution of isopropyl-β-D-1-thiogalactopyranoside (IPTG, 1.019 g, 4.3 mmol) and 4-dimethylaminopyridine (DMAP, 3 mg) as a catalyst dissolved in anhydrous pyridine (7 mL) was added slowly with benzoyl chloride (3.0 mL, 17.6 mmol), some white precipitates occurred and kept stirring until all the IPTG was consumed at room temperature. The reaction was monitored by TLC. Diluted the solution with CH_2Cl_2, and then washed with dilute HCl solution, saturated $NaHCO_3$ solution and brine. The organic layer was dried over anhydrous Na_2SO_4 and solvent was evaporated. The crude product was subjected to column chromatography using n-hexane:CH_2Cl_2 (25:1) as eluent to obtain isopropyl 2,3,4,6-tetra-*O*-benzoyl-1-thio-β-D-galactopyranoside (2.377 g, yield 85%) as a white solid. ^1H NMR (400 MHz, $CDCl_3$) δ (ppm): 8.09 (dd, J = 8.4 Hz, 1.3 Hz, 2H), 8.02 (dd, J = 8.5 Hz, 1.3 Hz, 2H), 7.95 (dd, J = 8.5 Hz, 1.3 Hz, 2H), 7.78 (dd, J = 8.4 Hz, 1.3 Hz, 2H), 7.36-7.64 (m, 10H), 7.22-7.26 (m, 2H), 6.03 (br d, J = 2.9 Hz, 1H), 5.81 (t, J = 10.0 Hz, 1H), 5.65 (dd, J = 9.9 Hz, 3.4 Hz, 1H), 4.95 (d, J = 10.0 Hz, 1H), 4.63-4.66 (m, 1H), 4.34-4.45 (m, 2H), 3.28 (sep, J = 6.8 Hz, 1H), 1.32 (d, J = 6.8 Hz, 6H). ^{13}C NMR (100 MHz, $CDCl_3$) δ (ppm): 166.1, 165.60, 165.56, 165.4, 133.7, 133.6, 133.3, 130.2, 130.0,

129.81, 129.77, 129.4, 129.3, 129.0, 128.8, 128.7, 128.49, 128.45, 128.4, 128.3, 84.1, 75.0, 72.8, 68.45, 68.41, 62.4, 35.8, 24.0, 23.8. FAB-MS (+ve): *m/z* 677.2 $[M + Na]^+$.

(b) Benzoylated capsicoside A_3 was synthesized similar to 3,6-dibenzoylated Timosaponin A I.[3] To a mixture of isopropyl 2,3,4,6-tetra-*O*-benzoyl-1-thio-β-D-galactopyranoside (327 mg, 0.50 mmol) and diosgenin (**6**, 207 mg, 0.50 mmol) in anhydrous CH_2Cl_2 (10 mL) was added NIS (123 mg, 0.55 mmol) and TMSOTf (8.8 μL, 0.005 mmol) under N_2 at -42 ^oC. The mixture was stirred under these conditions for 2 h, then neutralized with TEA and concentrated. The crude product was subjected to column chromatography using n-hexane:CH_2Cl_2:EtOAc (40:40:3) as eluent to obtain benzoylated capsicoside A_3 (135 mg, yield 28%) as a white solid. ^1H NMR (300 MHz, $CDCl_3$) δ (ppm): 8.10 (dd, J = 8.4 Hz, 1.2 Hz, 2H), 8.02 (dd, J = 8.5 Hz, 1.2 Hz, 2H), 7.97 (dd, J = 8.5 Hz, 1.2 Hz, 2H), 7.79 (dd, J = 8.4 Hz, 1.2 Hz, 2H), 7.35-7.63 (m, 10H), 7.21-7.23 (m, 2H), 5.99 (d, J = 3.1 Hz, 1H), 5.75-5.81 (m, 1H), 5.60 (dd, J = 10.3 Hz, 3.4 Hz, 1H), 5.22-5.24 (m, 1H), 4.92 (d, J = 8.0 Hz, 1H), 4.68 (dd, J = 11.2 Hz, 6.7 Hz, 1H), 4.31-4.45 (m, 3H), 3.34-3.60 (m, 3H), 2.19-2.20 (m, 2H), 1.60-2.00 (m, 11H), 1.37-1.51 (m, 4H), 1.01-1.27 (m, 7H), 0.97 (d, J = 6.8 Hz, 3H), 0.93 (s, 3H), 0.79 (d, J = 6.2 Hz, 3H), 0.77 (s, 3H). ^{13}C NMR (75 MHz, $CDCl_3$) δ (ppm): 166.4, 166.1, 166.0, 165.7, 140.7, 134.0, 133.64, 133.59, 130.5, 130.2, 130.1, 129.94, 129.9, 129.4, 129.2, 129.0, 128.9, 128.8, 128.7, 122.1, 109.7, 101.1, 81.20, 81.17, 72.3, 71.7, 70.4, 68.6, 67.3, 62.5, 56.9, 50.4, 42.0, 40.7, 40.2, 39.3, 37.5, 37.2, 32.5, 32.2, 31.8, 30.7, 30.0, 29.2, 21.2, 19.7, 17.6, 16.7, 15.0. ESI-MS (+ve): *m/z* 1015.4 $[M+Na]^+$.

(c) Benzoylated capsicoside A_3 (85 mg, 0.086 mmol) was dissolved in anhydrous CH_2Cl_2-MeOH (2:1, 7.5 mL) and then NaOMe in MeOH (0.1 mL, 1.0 M) was added dropwise at room temperature. After stirring at room temperature for 3.5 h, TLC (CH_2Cl_2: MeOH = 9:1) indicated that all starting material was consumed. The reaction mixture was neutralized with Amberlite IR 120 (H^+), and then filtered and concentrated. The product was subjected to column chromatography using CH_2Cl_2: MeOH (10:1) as eluent to obtain capsicoside A_3 (**8**) (46 mg, yield 93%) as a white solid. $[\alpha]^{25}_{D}$ -88 (*c* 0.41, 2:1 CH_2Cl_2/CH_3OH). ^1H NMR (600 MHz, MeOH-d_4) δ (ppm): 5.37 (m, 1H, H-6), 4.37-4.41 (m, 1H, H-16), 4.60 (br s, Gal-O<u>H</u>), 4.33 (d, J = 6.8 Hz, 1H, H-1'), 3.82 (d, J = 1.9 Hz, 1H, H-4'), 3.71-3.72 (m, 2H, H-6'), 3.56-3.60 (m, 1H, H-3), 3.44-3.50 (m, 4H, H-5', H-2', H-3', H-26a), 3.32-3.34 (m, 1H, H-26b), 2.42-2.44 (m, 1H, H-4a), 2.24-2.28 (m, 1H, H-4b), 1.86-2.02 (m, 5H), 1.50-1.77 (m, 11H), 1.40-1.43 (m, 1H), 1.26-1.31 (m, 1H), 1.12-1.22 (m, 2H), 1.05-1.10 (m, 1H), 1.04 (s, 3H, H-19), 0.98-1.00 (m, 1H), 0.96 (d, J = 7.0 Hz, 3H, H-21), 0.81 (s, 3H, H-18), 0.79 (d, J = 6.4 Hz, 3H, H-27). ^{13}C NMR (150 MHz, MeOH-d_4) δ (ppm): 142.2 (C-5), 122.6 (C-6), 110.7 (C-22), 103.2 (C-1'), 82.4 (C-16), 79.9 (C-3), 76.7 (C-5'), 75.2 (C-3'), 72.7 (C-2'), 70.4 (C-4'), 68.0 (C-26), 63.9 (C-17), 62.6 (C-6'), 57.9 (C-14), 51.8 (C-9), 43.1 (C-20), 41.6 (C-13), 41.1 (C-12), 39.9

(C-4), 38.7 (C-1), 38.2 (C-10), 33.3 (C-7), 33.0 and 32.9 (C-15, C-23), 32.6 (C-8), 31.6 (C-2), 30.9 (C-25), 30.0 (C-24), 22.1 (C-11), 20.0 (C-19), 17.7 (C-27), 16.9 (C-18), 15.0 (C-21). ESI-HRMS calcd for $C_{33}H_{53}O_8^+$: 577.3740 $[M+H]^+$; found: 577.3745.

5. Preparation of dihydrodioscin (11) and dihydropolyphyllin D (12) by hydrogenation

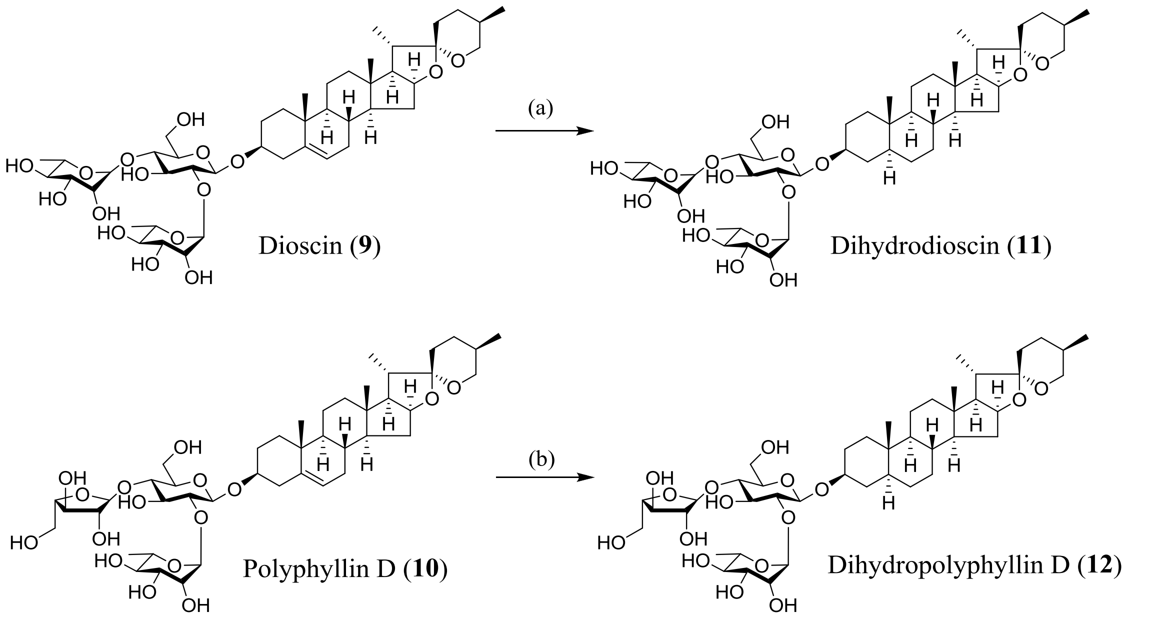

Fig. S5: Synthesis of dihydrodioscin (**11**) and dihydropolyphyllin D (**12**).

Hydrogenation of dioscin (**11**) and polyphyllin D (**12**) was referenced to published report.[9]

(a) To a solution of dioscin (**9**) (10 mg, 0.012 mmol) dissolved in MeOH (15 mL) was added Pd/charcoal (5%, 3 mg) as a catalyst. H_2 was bubbled in and stirred at room temperature for 3 h. Filtered and dried to obtain dihydrodioscin (**11**) (quantitative yield). $[\alpha]_D^{25}$ -70 (*c* 0.23, 2:1 CH_2Cl_2/CH_3OH). ^1H NMR (600 MHz, MeOH-d_4) δ (ppm): 5.17 (s, 1H), 4.82 (s, 1H), 4.49 (d, *J* = 7.8 Hz, 1H), 4.35-4.38 (m, 1H), 4.09-4.12 (m, 1H), 3.90-3.93 (m, 2H), 3.78-3.82 (m, 2H), 3.68-3.72 (m, 1H), 3.60-3.65 (m, 3H), 3.56 (t, *J* = 8.9 Hz, 1H), 3.50 (t, *J* = 9.2 Hz, 1H), 3.32-3.44 (m, 6H), 1.94-1.98 (m, 1H), 1.84-1.90 (m, 2H), 1.48-1.73 (m, 11H), 1.26-1.43 (m, 7H), 1.25 (d, *J* = 6.2 Hz, 3H), 1.22 (d, *J* = 6.2 Hz, 3H), 1.07-1.15 (m, 3H), 0.94 (d, *J* = 7.0 Hz, 3H), 0.88-0.99 (m, 2H), 0.85 (s, 3H), 0.77-0.79 (m, 6H), 0.65-0.69 (m, 1H). ^{13}C NMR (150 MHz, MeOH-d_4) δ (ppm): 110.7, 103.2, 102.5, 100.4, 82.4, 80.1, 79.6, 78.8, 78.2, 76.7, 74.1, 73.9, 72.6, 72.5, 72.3, 70.8, 69.9, 68.0, 64.0, 62.1, 57.7, 56.0, 46.2, 43.0, 41.9, 41.4, 38.5, 37.0, 36.7, 35.2, 33.7, 32.8, 32.6, 31.6, 30.9, 30.7, 30.0, 22.3, 18.1, 18.0, 17.6, 17.1, 15.0, 12.9. ESI-HRMS calcd for $C_{45}H_{75}O_{16}^+$: 871.5055 $[M+H]^+$; found: 875.5046.

(b) To a solution of polyphyllin D (**10**) (10 mg, 0.012 mmol) dissolved in MeOH (15 mL) was added Pd/charcoal (5%, 3 mg) as a catalyst. H_2 was bubbled in and stirred at room

temperature for 3 h. Filtered and dried to obtain dihydropolyphyllin D (**12**) (quantitative yield). $[\alpha]^{25}_{D}$ -79 (*c* 0.36, 2:1 CH_2Cl_2/CH_3OH). ^1H NMR (600 MHz, MeOH-d_4) δ (ppm): 5.19 (d, *J* = 1.2 Hz, 1H), 5.01 (d, *J* = 1.9 Hz, 1H), 4.50 (d, *J* = 7.8 Hz, 1H), 4.35-4.38 (m, 1H), 4.05-4.12 (m, 2H), 3.95-3.96 (m, 1H), 3.80-3.88 (m, 3H), 3.67-3.73 (m, 3H), 3.58-3.64 (m, 3H), 3.49 (t, *J* = 9.4 Hz, 1H), 3.42-3.44 (m, 1H), 3.32-3.39 (m, 4H), 1.94-1.98 (m, 1H), 1.84-1.90 (m, 2H), 1.47-1.73 (m, 11H), 1.23-1.43 (m, 7H), 1.21 (d, *J* = 6.2 Hz, 3H), 1.08-1.16 (m, 3H), 0.94 (d, *J* = 7.0 Hz, 3H), 0.88-0.99 (m, 2H), 0.85 (s, 3H), 0.78-0.79 (m, 6H), 0.65-0.69 (m, 1H). ^{13}C NMR (150 MHz, MeOH-d_4) δ (ppm): 110.7, 110.1, 102.3, 100.3, 86.0, 83.3, 82.4, 79.1, 78.9, 78.8, 78.2, 78.0, 76.6, 74.1, 72.5, 72.4, 69.9, 68.0, 64.0, 63.1, 62.1, 57.7, 56.0, 46.2, 43.1, 41.9, 41.4, 38.4, 37.0, 36.7, 35.3, 33.7, 32.8, 32.6, 31.6, 30.7, 30.0, 22.3, 18.1, 17.6, 17.1, 15.0, 12.9. ESI-HRMS calcd for $C_{44}H_{73}O_{16}^+$: 857.4899 $[M+H]^+$; found: 857.4890.

6. Preparation of Tigogenin (13) and Neotigogenin (14) by hydrogenation

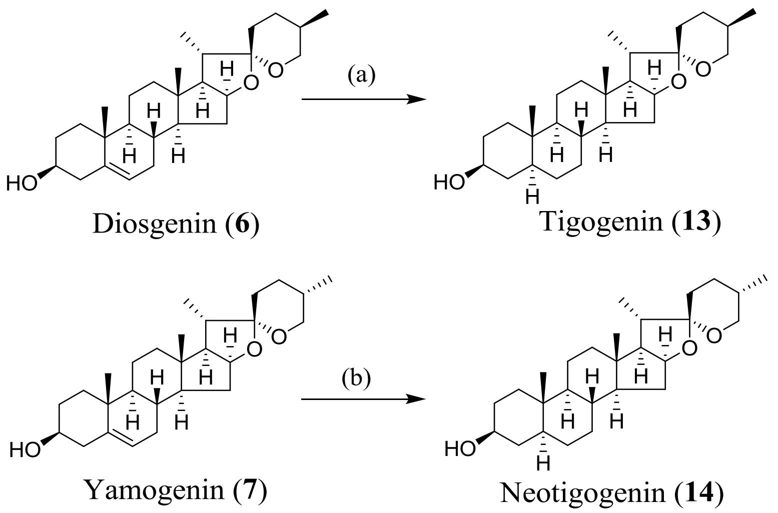

Fig. S6: Synthesis of Tigogenin (**13**) and Neotigogenin (**14**).

Hydrogenation of diosgenin (**6**) and yamogenin (**7**) was referenced to published report.[9]

(a) To a solution of diosgenin (**6**) (10 mg, 0.024 mmol) dissolved in MeOH (15 mL) was added Pd/charcoal (5%, 3 mg) as a catalyst. H_2 was bubbled in and stirred at room temperature for 3 h. Filtered and dried to obtain tigogenin (**13**) (quantitative yield). $[\alpha]^{22}_{D}$ -69 (*c* 0.53, CH_2Cl_2). ^1H NMR (400 MHz, $CDCl_3$) δ (ppm): 4.36-4.42 (m, 1H), 3.54-3.62 (m, 1H), 3.45-3.49 (m, 1H), 3.37 (t, *J* = 10.9 Hz, 1H), 1.95-2.01 (m, 1H), 1.21-1.89 (m, 20H), 1.05-1.16 (m, 3H), 0.96 (d, *J* = 7.0 Hz, 3H), 0.85-1.00 (m, 2H), 0.82 (s, 3H), 0.79 (d, *J* = 6.3 Hz, 3H), 0.76 (s, 3H), 0.61-0.70 (m, 1H),.^{13}C NMR (100 MHz, $CDCl_3$) δ (ppm): 109.2 (C-22), 80.8 (C-16), 71.3 (C-3), 66.8 (C-26), 62.2 (C-17), 56.3 (C-14), 54.4 (C-9), 44.8 (C-5), 41.6 (C-20), 40.6 (C-13), 40.1 (C-12), 38.2 (C-4), 37.0 (C-1), 35.6 (C-10), 35.1 (C-8), 32.2 (C-7), 31.8 (C-15), 31.5 (C-2), 31.4 (C-23), 30.3

(C-25), 28.8 (C-24), 28.6 (C-6), 21.1 (C-11), 17.1 (C-27), 16.5 (C-18), 14.5 (C-21), 12.3 (C-19). EI-HRMS calcd for $C_{27}H_{44}O_3^+$: 416.3285 $[M]^+$; found: 416.3297.

(b) To a solution of yamogenin (**7**) (5 mg, 0.012 mmol) dissolved in MeOH (7.5 mL) was added Pd/charcoal (5%, 1.5 mg) as a catalyst. H_2 was bubbled in and stirred at room temperature for 5 h. Filtered and dried to obtain neotigogenin (**14**) (quantitative yield). $[\alpha]_D^{22}$ -75 (*c* 0.07, CH_2Cl_2). ^1H NMR (400 MHz, $CDCl_3$) δ (ppm): 4.37-4.43 (m, 1H, H-16), 3.95 (dd, *J* = 10.9 Hz, 2.7 Hz, 1H, H-26b), 3.55-3.62 (m, 1H, H-3), 3.29 (br d, *J* = 10.9 Hz, 1H, H-26a), 1.94-2.07 (m, 2H), 1.65-1.88 (m, 8H), 1.35-1.56 (m, 7H), 1.21-1.31 (m, 5H), 1.08 (d, *J* = 7.1 Hz, 3H, H-27), 1.05-1.16 (m, 2H), 0.99 (d, *J* = 6.8 Hz, 3H, H-21), 0.85-0.97 (m, 2H), 0.82 (s, 3H, H-19), 0.76 (s, 3H, H-18), 0.61-0.68 (m, 1H). EI-HRMS calcd for $C_{27}H_{44}O_3^+$: 416.3285 $[M]^+$; found: 416.3280.

7. Preparation of 5α-H, 6α-OH and 5β-H, 6β-OH of diosgenin (15-16) and 5α-H, 6α-OH and 5β-H, 6β-OH of yamogenin (17-18).

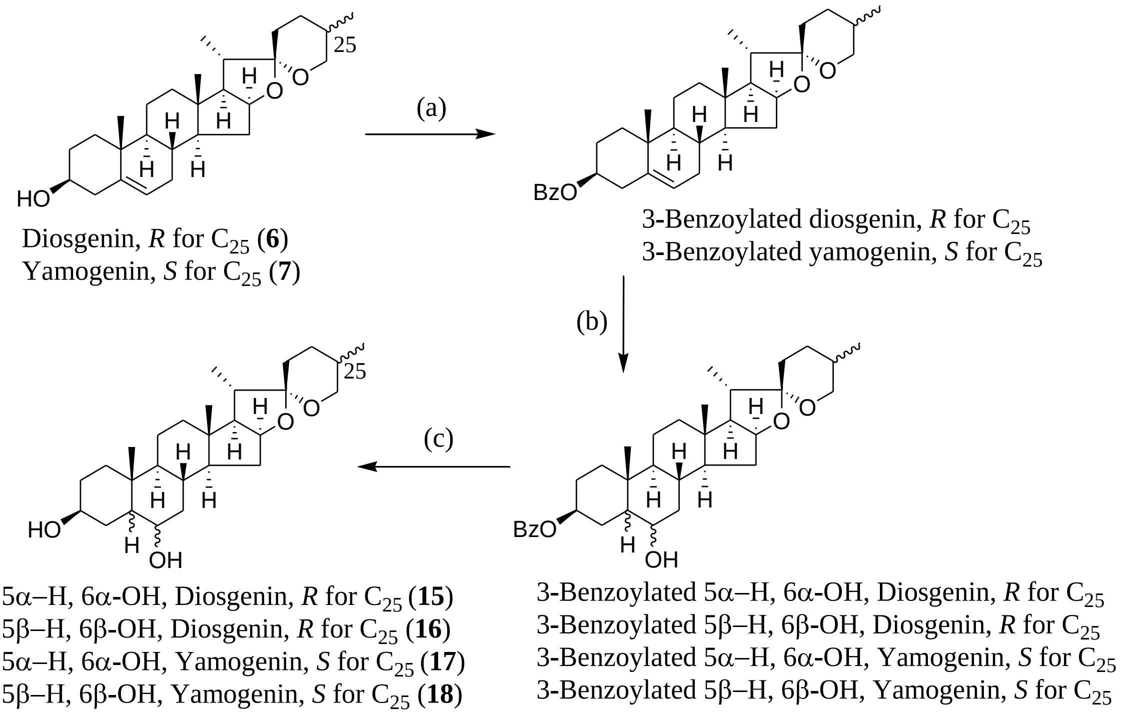

Fig. S7: Synthesis of 5α-H, 6α-OH and 5β-H, 6β-OH of diosgenin and yamogenin.

The synthesis of compounds 15-18 was referenced to publication. [10]

(a) To a solution of diosgenin (**6**) (125 mg, 0.3 mmol) in anhydrous CH_2Cl_2 (15 mL) at 0 ℃, benzoyl chloride (65 mg), Et_3N (50 mg) and DMAP (10 mg) were added subsequently. The reaction mixture was warmed to room temperature gently and monitored by TLC (~ 8 h). The mixture was concentrated under reduced pressure and the residue was subjected to column chromatography using EtOAc/n-hexane as eluent to obtain 3-benzoylated diosgenin (Yield: 85%). ^1H NMR (400 MHz, $CDCl_3$) δ (ppm): 8.04 (d, *J* = 7.1 Hz, 2H),

7.54 (m, 1H), 7.44 (m, 2H), 5.42 (d, *J* = 4.6 Hz, 1H), 4.83 (m, 1H), 4.43 (q, *J* = 7.4 Hz, 1H), 3.49 (m, 1H), 3.39 (m 1H), 2.48 (m, 2H), 2.04-1.98 (m, 3H), 1.94-1.84 (m, 2H), 1.82-1.72 (m, 3H), 1.70-1.57 (m, 6H), 1.56-1.48 (m, 3H), 1.31-1.08 (m, 4H), 1.10 (s, 3H), 1.07-1.01 (m, 1H), 0.99 (d, *J* = 6.9 Hz, 3H), 0.81 (s, 3H), 0.80 (d, *J* = 6.9 Hz, 3H). ^{13}C NMR (100 MHz, $CDCl_3$) δ (ppm): 166.0, 139.7, 132.7, 130.8, 129.5, 128.3, 122.5, 109.3, 80.8, 74.5, 66.8, 62.1, 56.5, 50.0, 41.6, 40.3, 39.7, 38.2, 37.0, 36.8, 32.1, 31.9, 31.4, 31.4, 30.3, 28.8, 27.9, 20.8, 19.4, 17.2, 16.3, 14.6. ESI-HRMS calcd for $C_{34}H_{47}O_4^+$: 519.3474 $[M+H]^+$; found: 519.3480.

(b) To a solution of 3-benzoylated diosgenin (52 mg, 0.1 mmol) in anhydrous THF (5 mL) at 0 ℃, Et_2O•BH_3 (0.15 mmol) was added. The reaction mixture was warmed to room temperature gently and monitored by TLC (~ 12 h). Na_2CO_3 (200 mg), H_2O (2 mL) and H_2O_2 (200 μL) were added slowly to the mixture and stirred for 1 h. Then, the mixture was extracted with CH_2Cl_2 (3 × 20 mL) and the organic fraction was dried by $MgSO_4$, concentrated under reduced pressure. The residue was subjected to column chromatography using EtOAc/n-hexane as eluent to obtain 3-benzoylated 5β-H, 6β-OH diosgenin (Yield: 29%) and 3-benzoylated 5α-H, 6α-OH diosgenin (Yield: 44%).

3-Benzoylated 5α-H, 6α-OH diosgenin: ^{1}H NMR (400 MHz, $CDCl_3$) δ (ppm): 8.03 (d, *J* = 7.1 Hz, 2H), 7.54 (m, 1H), 7.43 (m, 2H), 4.93 (m, 1H), 4.41 (q, *J* = 7.1 Hz, 1H), 3.49 (m, 1H), 3.45 (br s, 1H), 3.37 (m 1H), 2.35 (m, 1H), 2.06-1.96 (m, 3H), 1.94-1.89 (m, 1H), 1.80-1.75 (m, 2H), 1.74-1.57 (m 8H), 1.56-1.34 (m, 4H), 1.31-1.21 (m, 2H), 1.20-1.14 (m, 4H), 0.97 (d, *J* = 6.9 Hz, 3H), 0.90 (s, 3H), 0.79 (d, *J* = 6.9 Hz, 3H), 0.77 (s, 3H), 0.78 (m, 1H). ^{13}C NMR (100 MHz, $CDCl_3$) δ (ppm): 166.0, 132.7, 130.8, 129.5, 128.2, 109.2, 80.7, 74.2, 69.2, 66.8, 62.1, 55.9, 53.6, 51.6, 41.8, 41.6, 40.5, 39.7, 37.0, 36.4, 33.9, 31.7, 31.3, 30.2, 28.8, 28.4, 27.3, 20.9, 17.1, 16.4, 14.5, 13.4. ESI-HRMS calcd for $C_{34}H_{49}O_5^+$: 537.3580 $[M+H]^+$; found: 537.3571.

3-Benzoylated 5β-H, 6β-OH diosgenin: ^{1}H NMR (400 MHz, $CDCl_3$) δ (ppm): 8.03 (d, *J* = 7.1 Hz, 2H), 7.55 (m, 1H), 7.43 (m, 2H), 4.99 (m, 1H), 4.40 (q, *J* = 7.1 Hz, 1H), 3.84 (br s, 1H), 3.48 (m, 1H), 3.38 (m 1H), 2.02-1.93 (m, 4H), 1.92-1.84 (m, 4H), 1.80-1.72 (m, 2H), 1.70-1.52 (m, 6H), 1.51-1.35 (m, 2H), 1.33-1.27 (m, 3H), 1.24-1.02 (m, 4H), 1.12 (s, 3H), 0.97 (d, *J* = 6.9 Hz, 3H), 0.81 (s, 3H), 0.79 (d, *J* = 6.9 Hz, 3H), 0.78 (m, 1H). ^{13}C NMR (100 MHz, $CDCl_3$) δ (ppm): 166.2, 132.8, 130.8, 129.5, 128.3, 109.3, 80.8, 74.5, 71.6, 66.9, 62.2, 56.0, 54.1, 47.2, 41.6, 40.6, 40.0, 39.9, 38.3, 35.6, 31.8, 31.5, 31.4, 30.3, 30.0, 28.8, 27.5, 20.9, 17.1, 16.5, 15.7, 14.5. ESI-HRMS calcd for $C_{34}H_{49}O_5^+$: 537.3580 $[M+H]^+$; found: 537.3568.

(c) To a solution of 3-benzoylated 5β-H, 6β-OH diosgenin (27 mg, 0.05 mmol) in MeOH-H_2O (5:1, 6 mL), K_2CO_3 (30 mg) was added. The reaction mixture was refluxed and monitored by TLC (~ 5 h). The mixture was extracted with CH_2Cl_2 (3 × 20 mL) and the organic fraction was dried by $MgSO_4$, concentrated under reduced pressure. The

residue was purified by column chromatography using EtOAc/n-hexane as eluent to obtain 5β-H, 6β-OH diosgenin (**16**) (Yield: 87%). $[\alpha]^{25}_{D}$ -39 (*c* 0.43, 3:1 CH_2Cl_2/CH_3OH). ^1H NMR (400 MHz, $CDCl_3$) δ (ppm): 4.41 (q, *J* = 7.5 Hz, 1H), 3.81 (br s, 1H), 3.64 (m, 1H), 3.46 (m, 1H), 3.37 (t, *J* = 10.7 Hz, 1H), 2.03-1.94 (m, 2H), 1.92-1.71 (m, 5H), 1.70-1.59 (m, 7H), 1.52-1.45 (m, 3H), 1.44-1.35 (m, 2H), 1.32-1.23 (m, 3H), 1.20-1.07 (m, 4H), 1.05 (s, 3H), 0.96 (d, *J* = 6.8 Hz, 3H), 0.80 (s, 3H), 0.79 (d, *J* = 6.3 Hz, 3H), 0.72-0.66 (m, 1H). ^{13}C NMR (100 MHz, $CDCl_3$) δ (ppm): 109.3, 80.8, 71.9, 71.7, 66.9, 62.2, 56.1, 54.3, 47.4, 41.7, 40.6, 40.0, 39.8, 38.5, 35.5, 35.4, 31.8, 31.5, 31.4, 30.3, 30.0, 28.8, 20.9, 17.2, 16.6, 15.8, 14.5. EI-HRMS calcd for $C_{27}H_{44}O_4^+$: 432.3235 $[M]^+$; found: 432.3230.

5α-H, 6α-OH diosgenin (**15**) was prepared in similar manner. $[\alpha]^{25}_{D}$ -77 (*c* 0.22, CH_2Cl_2). ^1H NMR (300 MHz, $CDCl_3$) δ (ppm): 4.41 (q, *J* = 7.4 Hz, 1H), 4.28 (br s, 2H), 3.51 (m, 2H), 3.35 (m, 2H), 2.15-2.10 (m, 1H), 2.04-1.97 (m, 2H), 1.91-1.85 (m, 1H), 1.80-1.73 (m, 3H), 1.73-1.62 (m, 5H), 1.61-1.50 (m, 1H), 1.48-1.37 (m, 2H), 1.32-1.24 (m, 3H), 1.21-1.12 (m, 2H), 1.05-0.95 (m, 2H), 0.97 (d, *J* = 6.9 Hz, 3H), 0.92-0.85 (m, 1H), 0.87 (s, 3H), 0.80 (d, *J* = 6.3 Hz, 3H), 0.77 (s, 3H), 0.74-0.66 (m, 1H). ^{13}C NMR (100 MHz, $CDCl_3$) δ (ppm): 109.3, 80.6, 70.4, 68.5, 66.6, 61.8, 55.7, 53.5, 51.2, 41.3, 41.0, 40.3, 39.5, 37.0, 36.0, 33.6, 31.4, 31.3, 31.0, 30.2, 29.9, 28.3, 20.6, 16.6, 16.0, 14.0, 13.0. EI-HRMS calcd for $C_{27}H_{44}O_4^+$: 432.3235 $[M]^+$; found: 432.3230.

5α-H, 6α-OH yamogenin (**17**) and 5β-H, 6β-OH yamogenin (**18**) were prepared in similar manner. 5α-H, 6α-OH yamogenin (**17**) (Yield: 85%). $[\alpha]^{24}_{D}$ -37 (*c* 0.16, 2:1 CH_2Cl_2/CH_3OH). ^1H NMR (600 MHz, $CDCl_3$) δ (ppm): 4.42 (q, *J* = 7.4 Hz, 1H, H-16), 3.95 (dd, *J* = 11.0, 2.5 Hz, 1H, H-26α), 3.59 (m, 1H, H-3), 3.45 (dt, *J* = 10.7, 4.5 Hz, 1H, H-6), 3.30 (d, *J* = 11.0 Hz, 1H, H-26β), 2.21-2.17 (m, 1H), 2.04-1.97 (m, 3H), 1.91-1.85 (m, 1H), 1.84-1.76 (m, 3H), 1.74-1.66 (m, 3H), 1.66-1.62 (m, 2H), 1.54-1.50 (m, 1H), 1.46-1.37 (m, 4H), 1.32-1.22 (m, 3H), 1.21-1.10 (m, 2H), 1.08 (d, *J* = 7.1 Hz, 3H, H-27), 1.05-0.95 (m, 2H), 0.99 (d, *J* = 6.8 Hz, 3H, H-21), 0.92-0.85 (m, 1H), 0.83 (s, 3H, H-19), 0.76 (s, 3H, H-18), 0.72-0.66 (m, 1H). Significant NoE effect was observed for H-6 and H-19. ^{13}C NMR (150 MHz, $CDCl_3$) δ (ppm): 109.8 (C-22), 80.8 (C-16), 71.2 (C-3), 69.3 (C-6), 65.2 (C-26), 61.9, 56.0, 53.8, 51.6, 42.1, 41.8, 40.5, 39.9, 37.2, 36.4, 33.9, 32.3, 31.7, 31.0, 27.1, 25.9, 25.8, 21.0, 16.5 (C-18), 16.1 (C-27), 14.3 (C-21), 13.5 (C-19). EI-HRMS calcd for $C_{27}H_{44}O_4^+$: 432.3235 $[M]^+$; found: 432.3233.

5β-H, 6β-OH yamogenin (**18**) (Yield: 83%). $[\alpha]^{24}_{D}$ -32 (*c* 0.31, 2:1 CH_2Cl_2/CH_3OH). ^1H NMR (600 MHz, $CDCl_3$) δ (ppm): 4.40 (q, *J* = 7.5 Hz, 1H, H-16), 3.94 (dd, *J* = 11.0, 2.3 Hz, 1H, H-26α), 3.81 (d, *J* = 2.2 Hz, 1H, H-6), 3.65 (m, 1H, H-3), 3.30 (d, *J* = 11.0 Hz, 1H, H-26β), 2.16-1.96 (m, 2H), 1.94-1.86 (m, 2H), 1.85-1.79 (m, 3H), 1.79-1.75 (m, 1H), 1.74-1.66 (m, 3H), 1.66-1.62 (m, 2H), 1.54-1.50 (m, 1H), 1.49-1.35 (m, 4H), 1.34-1.25 (m, 3H), 1.18-1.09 (m, 4H), 1.08 (d, *J* = 7.1 Hz, 3H, H-27), 1.05 (s, 3H, H-19), 0.99 (d, *J*

= 6.9 Hz, 3H, H-21), 1.01-0.94 (m, 1H), 0.80 (s, 3H, H-18), 0.72-0.66 (m, 1H). ^{13}C NMR (150 MHz, $CDCl_3$) δ (ppm): 109.8 (C-22), 80.9 (C-16), 71.9 (C-6), 71.7 (C-3), 65.2 (C-26), 62.0, 56.0, 54.3, 47.3, 42.1, 40.6, 40.0, 39.7, 38.5, 35.5, 35.3, 31.7, 31.5, 30.0, 27.1, 25.9, 25.7, 20.9, 16.6 (C-18), 16.1 (C-27), 15.8 (C-19), 14.3 (C-21). EI-HRMS calcd for $C_{27}H_{44}O_4^+$: 432.3235 $[M]^+$; found: 432.3228.

8. Preparation of Sarsasapogenone (19) and Episarsasapogenin (20)

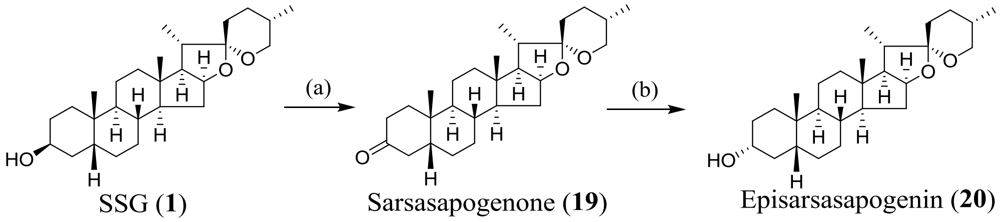

Fig. S8: Synthesis of sarsasapogenone (**19**) and episarsasapogenin (**20**).

(a) Sarsasapogenone was prepared according to literature.[11] To a solution of sarsasapogenin (**1**) (500 mg, 1.20 mmol) in acetone (25 mL) at 30 ^{o}C was added 8N chromic acid in H_2SO_4 (40%, 1.5 mL) and the mixture was stirred for 30 min. The reaction completion was monitored by TLC (n-Hexane:EtOAc = 3:1). The mixture was dried to afford a crude product which was added with H_2O and extracted with CH_2Cl_2. The dry crude product was subjected to column chromatography using n-hexane: EtOAc (5:1) as eluent to give sarsasapogenone as a white solid (**19**) (251 mg, yield 50%). $[\alpha]^{25}_{D}$ -56 (*c* 0.29, CH_2Cl_2). ^{1}H NMR (500 MHz, $CDCl_3$) δ (ppm): 4.40-4.44 (m, 1H, H-16), 3.95 (dd, *J* = 10.9 Hz, 2.4 Hz, 1H, H-26b), 3.30 (d, *J* = 10.9 Hz, 1H, H-26a), 2.69 (t, *J* = 14.3 Hz, 1H), 2.32 (td, *J* = 14.6 Hz, 5.2 Hz, 1H), 2.14-2.17 (m, 1H), 1.99-2.04 (m, 4H), 1.61-1.93 (m, 8H), 1.35-1.55 (m, 7H), 1.11-1.32 (m, 5H), 1.08 (d, *J* = 7.1 Hz, 3H, H-27), 1.04 (s, 3H, H-19), 1.00 (d, *J* = 6.7 Hz, 3H, H-21), 0.80 (s, 3H, H-18). ^{13}C NMR (125 MHz, $CDCl_3$) δ (ppm): 213.0 (C-3), 109.6 (C-22), 80.8 (C-16), 65.1 (C-26), 62.1 (C-17), 56.2 (C-14), 44.2 (C-5), 42.3 (C-4), 42.1 (C-20), 40.8 (C-9), 40.6 (C-13), 40.1 (C-12), 37.1 (C-2), 37.0 (C-1), 35.1 (C-8), 35.0 (C-10), 31.7 (C-15), 27.0 (C-25), 26.5 (C-6), 26.0 (C-7), 25.9 (C-23), 25.7 (C-24), 22.6 (C-19), 21.0 (C-11), 16.4 (C-18), 16.00 (C-27), 14.3 (C-21). EI-HRMS calcd for $C_{27}H_{42}O_3^+$: 414.3128 $[M]^+$; found: 414.3119.

(b) Episarsasapogenin was prepared according to literature.[12] To a solution of sarsasapogenone (**19**) (50 mg, 0.12 mmol) in THF (1.5 mL) at room temperature was added lithium tri-tert-butoxyaluminohydride (0.16 mL, 1M in THF) at such a rate that the temperature was maintained. After the addition was complete, the mixture was stirred at room temperature overnight. TLC (CH_2Cl_2:EtOAc=10:1) indicated that all sarsasapogenone was consumed. Saturated NH_4Cl solution was added to quench the reducing reagent. The mixture was filtered and the solid was washed with CH_2Cl_2. The

combined filtrates were extracted with CH_2Cl_2 and the organic layer was evaporated to afford a crude product which was subjected to column chromatography using CH_2Cl_2 : EtOAc (10:1) as eluent to afford sarsasapogenin (**1**) (8 mg, yield 8%) and episarsasapogenin (**20**) (80 mg, yield 80%). $[\alpha]_D^{22}$ -68 (*c* 0.52, CH_2Cl_2). ^{1}H NMR (400 MHz, $CDCl_3$) δ (ppm): 4.38-4.44 (m, 1H, H-16), 3.95 (br d, *J* = 9.9 Hz, 1H, H-26b), 3.60-3.65 (m, 1H, H-3), 3.30 (br d, J=10.9 Hz, 1H, H-26a), 1.95-2.07 (m, 2H), 1.65-1.91 (m, 10H), 1.39-1.59 (m, 8H), 1.12-1.33 (m, 7H), 1.08 (d, *J* = 7.0 Hz, 3H, H-27), 0.99 (d, *J* = 6.6 Hz, 3H, H-21), 0.94 (s, 3H, H-19), 0.75 (s, 3H, H-18). ^{13}C NMR (100 MHz, $CDCl_3$) δ (ppm): 109.7 (C-22), 81.0 (C-16), 71.8 (C-3), 65.1 (C-26), 62.0 (C-17), 56.3 (C-14), 42.1 (C-20), 42.0 (C-5), 40.6 (C-13), 40.5 (C-9), 40.2 (C-12), 36.4 (C-4), 35.5 (C-8), 35.3 (C-1), 34.7 (C-10), 31.7 (C-15), 30.5 (C-2), 27.07 and 27.06 (C-25, C-7), 26.7 (C-6), 25.9 (C-23), 25.8 (C-24), 23.4 (C-19), 20.6 (C-11), 16.5 (C-18), 16.0 (C-27), 14.3 (C-21). EI-HRMS calcd for $C_{27}H_{44}O_3^+$: 416.3285 $[M]^+$; found: 416.3292.

9. **Preparation of dihydrosarsasapogenin (dSSG, 21)**

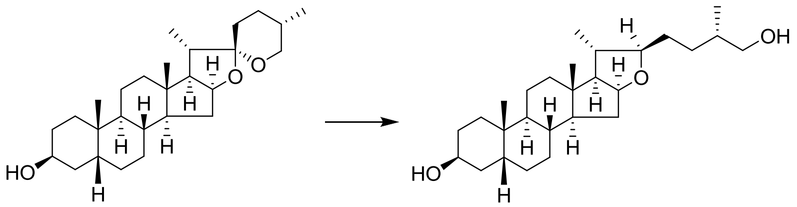

SSG (**1**) Dihydrosarsasapogenin (dSSG, **21**)

Fig. S9: Synthesis of dSSG (**21**).

Dihydrosarsasapogenin was prepared according to reference.[13] To a solution of $AlCl_3$ (0.267 g, 2.00 mmol) dissolved in anhydrous Et_2O (3 mL) at 0 ^oC was added ethereal $LiAlH_4$ solution (0.5 mL, 1.0 M) and stirred for 0.5 h. Sarsasapogenin (**1**, 417 mg, 1.0 mmol) was then added and the mixture was stirred overnight at room temperature after removal of the ice bath. The reaction was quenched by addition of H_2SO_4 (10%, 20 mL). The clear ether layer was separated and aqueous layer was extracted with Et_2O thrice. The organic layers were combined, dried with anhydrous Na_2SO_4, filtered and concentrated to afford a crude product which was then subjected to column chromatography using MeOH:CH_2Cl_2 (1:30) as eluent to afford dihydrosarsasapogenin (**21**) (160 mg, yield 38%), $[\alpha]_D^{22}$ -7 (*c* 0.83, CH_2Cl_2). ^{1}H NMR (400 MHz, $CDCl_3$) δ (ppm): 4.26-4.31 (m, 1H, H-16), 4.10 (br s, 1H, H-3), 3.43-3.52 (m, 2H, H-26), 3.30-3.34 (m, 1H, H-22), 1.86-2.03 (m, 3H), 1.49-1.75 (m, 11H), 1.07-1.45 (m, 11H), 0.98-1.00 (m, 6H, H-19, H-27), 0.93 (d, *J* = 5.9 Hz, 3H, H-21), 0.78 (s, 3H, H-18). ^{13}C NMR (100 MHz, $CDCl_3$) δ (ppm): 90.5 (C-22), 83.3 (C-16), 67.9 (C-26), 67.1 (C-3), 65.4 (C-17), 56.9 (C-14), 41.1 (C-13), 39.91 (C-12), 39.85 (C-9), 37.9 (C-20), 36.5 (C-5), 36.0 (C-25), 35.4 (C-8), 35.2 (C-10), 33.5 (C-4),

32.2 (C-15), 30.7 (C-23), 30.05 (C-24), 29.95 (C-1), 27.8 (C-2), 26.54 and 26.45 (C-6, C-7), 23.9 (C-19), 20.7 (C-11), 19.0 (C-21), 16.8 (C-27), 16.6 (C-18). EI-HRMS calcd for $C_{27}H_{44}O_3^+$: 418.3442 $[M]^+$; found: 418.3432.

10. Preparation of propargyl SSG (25), azide (26) triazole SSG (27) and α-OMe triazole SSG (28)

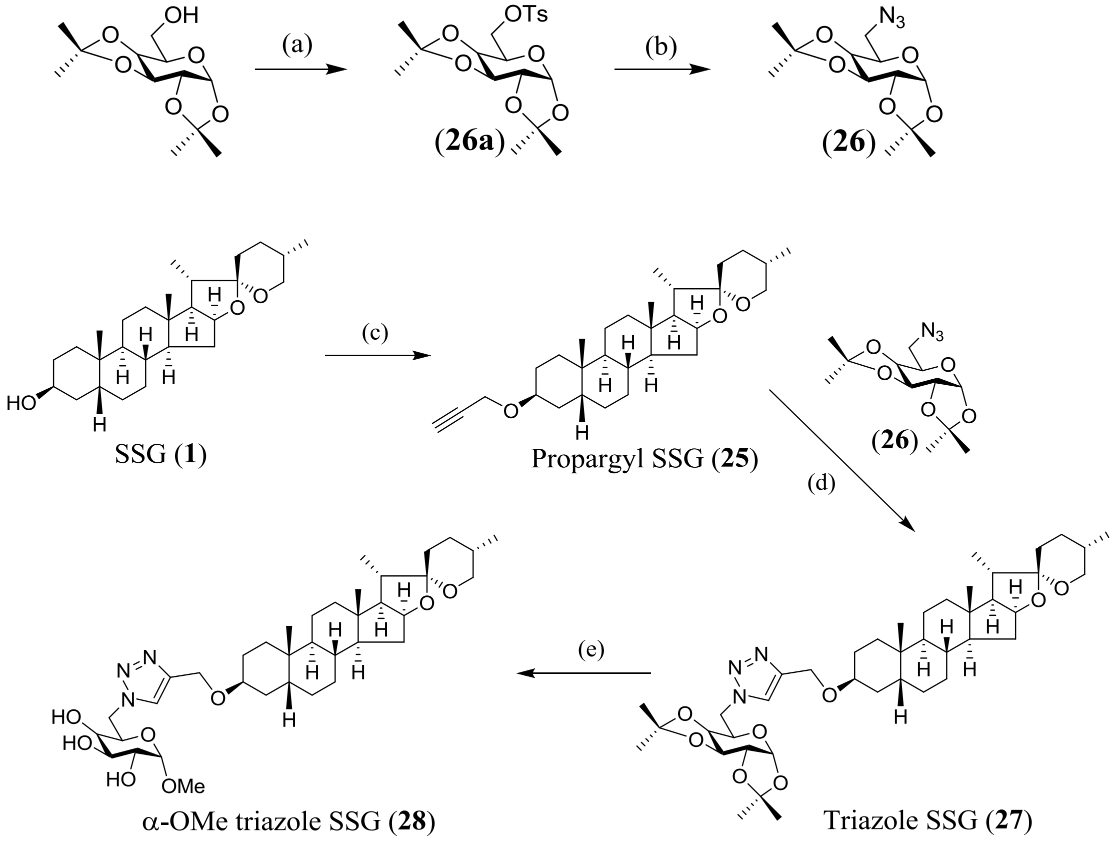

Fig. S10: Synthesis of compounds **25-28**.

(a) Compounds **26** was prepared according to the literature.[14] Briefly, reaction of commercial 1,2;3,4-di-*O*-isopropylidene-α-D-galactopyranose (1.0 eq) with tosyl chloride (1.5 eq) and Et_3N (2.0 eq) in anhydrous CH_2Cl_2 overnight gave the tosylated product **26a** in 92% yield after purification by flash column chromatography: ^{1}H NMR (400 MHz, $CDCl_3$) δ (ppm): 7.82 (d, J = 8.0 Hz, 2H), 7.33 (d, J = 8.2 Hz, 2H), 5.45 (d, J = 5.0 Hz, 1H), 4.58 (dd, J = 7.9, 2.5 Hz, 1H), 4.29 (dd, J = 5.0, 2.5 Hz, 1H), 4.22-4.18 (m, 2H), 4.11-4.04 (m, 2H), 2.44 (s, 3H), 1.50 (s, 3H), 1.34 (s, 3H), 1.31 (s, 3H), 1.28 (s, 3H). ^{13}C NMR (100 MHz, $CDCl_3$) δ (ppm): 144.8, 132.8, 129.7, 128.1, 109.5, 108.9, 96.1, 70.5, 70.4, 70.3, 68.2, 65.8, 25.9, 25.8, 24.9, 24.3, 21.6.

(b) Reaction of **26a** with NaN_3 (5.0 eq) in DMF at 120 ℃ for 24 hours gave **26** in 90% yield

after purification by flash column chromatography. ^{1}H NMR (400 MHz, $CDCl_3$) δ (ppm): 5.54 (d, J = 5.0 Hz, 1H), 4.64 (dd, J = 7.9, 2.4 Hz, 1H), 4.33 (dd, J = 5.0, 2.5 Hz, 1H), 4.20 (dd, J = 7.9, 1.9 Hz, 1H), 3.93-3.90 (m, 1H), 3.51 (dd, J = 12.7, 7.9 Hz, 1H), 3.36 (dd, J = 12.7, 5.3 Hz, 1H), 1.55 (s, 3H), 1.46 (s, 3H), 1.34 (s, 3H), 1.33 (s, 3H). ^{13}C NMR (100 MHz, CDCl3) δ (ppm): 109.5, 108.7, 96.3, 71.1, 70.7, 70.3, 66.9, 50.6, 25.9, 25.9, 24.8, 24.3.

(c) To a solution of SSG (**1**) (42 mg, 0.1 mmol) in anhydrous DMF (3 mL), NaH (0.5 mmol) was added at 0 °C and the mixture was stirred at this temperature for 0.5 h. Then, propargyl bromide (1.0 mmol) was added and the mixture was stirred at room temperature for 3 d. The reaction mixture was diluted with 100 mL of diethyl ether and drops of H_2O was added to quench the unreacted NaH, and then, the diethyl ether fraction was washed successively with water, brine, dried with $MgSO_4$, and concentrated under reduced pressure. The residue was subject to column chromatography using EtOAc/n-hexane as eluent to afford propargyl SSG (**25**) (Yield: 46%), $[\alpha]^{25}_{D}$ -51 (c 0.52, 2:1 CH_2Cl_2/CH_3OH). ^{1}H NMR (400 MHz, $CDCl_3$) δ (ppm): 4.40 (m, 1H), 4.12 (s, 2H), 3.92 (dd, J = 11.0, 2.3 Hz, 1H), 3.83 (s, 1H), 3.26 (d, J = 11.0 Hz, 1H), 2.37 (s, 1H), 2.02-1.95 (m, 2H), 1.91-1.82 (m, 3H), 1.81-1.74 (m, 2H), 1.73-1.54 (m, 5H), 1.47-1.37 (m, 6H), 1.36-1.23 (m, 5H), 1.22-1.13 (m, 3H), 1.08-1.03 (m, 1H), 1.07 (d, J = 7.1 Hz, 3H), 0.99 (d, J = 6.9 Hz, 3H), 0.95 (s, 3H), 0.76 (s, 3H). ^{13}C NMR (100 MHz, $CDCl_3$) δ (ppm): 109.7, 81.0, 80.7, 73.8, 73.5, 65.1, 62.2, 56.5, 55.1, 42.1, 40.7, 40.4, 40.1, 36.9, 35.3, 35.1, 31.8, 30.5, 30.3, 27.1, 26.6, 26.5, 26.0, 25.8, 24.4, 23.8, 20.9, 16.5, 16.1, 14.4. ESI-HRMS calcd for $C_{30}H_{47}O_3^+$: 455.3525 $[M+H]^+$; found: 455.3533.

(d) To a solution of **25** (23 mg, 0.05 mmol) and **26** (45 mg, 0.15 mmol) in 3 mL of de-gased mixed solvent of *tert*-butanol/DMSO (4:1, v/v) at room temperature, $CuSO_4 \cdot 5H_2O$ (2 mg, dissolved in 25 μL of H_2O) was added and the mixture was stirred for 5 min. Then, ascorbic acid (5 mg) was added and the mixture was stirred for 24 h. The reaction mixture was diluted with 100 mL of diethyl ether, washed with water, brine, dried with $MgSO_4$, and concentrated under reduced pressure. The residue was subject to column chromatography using EtOAc/n-hexane as eluent to afford triazole SSG (**27**) (Yield: 63%). $[\alpha]^{25}_{D}$ -48 (c 0.58, 2:1 CH_2Cl_2/CH_3OH). ^{1}H NMR (400 MHz, $CDCl_3$) δ (ppm): 7.70 (s, 1H), 5.51 (d, J = 4.9 Hz, 1H), 4.66-4.58 (m, 4H), 4.48-4.38 (m, 2H), 4.33 (m, 1H), 4.20 (m, 2H), 3.95 (dd, J = 11.2, 2.4 Hz, 1H), 3.74 (s, 1H), 3.30 (d, J = 11.2 Hz, 1H), 2.03-1.93 (m, 2H), 1.92-1.72 (m, 5H), 1.71-1.66 (m, 3H), 1.64-1.54 (m, 2H), 1.50 (s, 3H), 1.46-1.34 (m, 6H), 1.40 (s, 3H), 1.36 (s, 3H), 1.33-1.23 (m, 5H), 1.29 (s, 3H), 1.22-1.12 (m, 3H), 1.07 (d, J = 7.1 Hz, 3H), 1.08-1.03 (m, 1H), 0.99 (d, J = 6.9 Hz, 3H), 0.95 (s, 3H), 0.75 (s, 3H). ^{13}C NMR (100 MHz, $CDCl_3$) δ (ppm): 146.0, 123.6, 109.8, 109.7, 109.1, 96.3, 81.0, 73.9, 71.2, 70.7, 70.3, 67.2, 65.1, 62.1, 61.5, 56.5, 50.4, 42.1, 40.7, 40.3, 40.1, 36.9, 35.3, 35.1, 31.8, 30.7, 30.4, 27.1, 26.7, 26.6, 26.0, 25.8, 24.9, 24.6,

24.4, 23.9, 20.9, 16.5, 16.1, 14.4. ESI-HRMS calcd for $C_{42}H_{66}N_3O_8^+$: 740.4850 [M+H]$^+$; found: 740.4830.

(e) The solution of **27** (15 mg) in a mixed solvent of MeOH (1 mL) and concentrated HCl (1 mL) was stirred at 40 ℃ for 3 h. Then the mixture was dried in vacuuo and the residue was dissolved in 10 mL of anhydrous MeOH and 1 mL of HCl in diethyl ether (1 M) was added. The reaction mixture was stirred over night at room temperature. The mixture was dried in vacuuo and the residue was purified by flash column chromatography to give the product (**28**) (Yield: 58%). $[\alpha]_D^{25}$ -58 (*c* 0.12, 2:1 CH_2Cl_2/CH_3OH). ^{1}H NMR (400 MHz, $CDCl_3$) δ (ppm): 7.58 (s, 1H), 4.96 (s, 1H), 4.78 (br s, 1H), 4.53-4.33 (m, 6H), 4.15 (s, 1H), 4.11 (d, *J* = 9.7 Hz, 1H), 4.03 (s, 1H), 3.95 (dd, *J* = 11.0, 2.5 Hz, 1H), 3.73 (s, 1H), 3.42 (s, 3H), 3.29 (d, *J* = 11.0 Hz, 1H), 2.99 (d, *J* = 10.7 Hz, 1H), 2.03-1.93 (m, 2H), 1.90-1.72 (m, 5H), 1.70-1.54 (m, 5H), 1.48-1.33 (m, 6H), 1.32-1.22 (m, 5H), 1.20-1.10 (m, 3H), 1.08-1.03 (m, 1H), 1.07 (d, *J* = 7.1 Hz, 3H), 0.99 (d, *J* = 6.9 Hz, 3H), 0.95 (s, 3H), 0.75 (s, 3H). ^{13}C NMR (125 MHz, $CDCl_3$) δ (ppm): 146.0, 123.9, 109.8, 109.7, 87.5, 81.0, 78.7, 78.6, 74.7, 69.9, 65.2, 62.1, 61.4, 56.5, 55.1, 53.4, 42.2, 40.7, 40.3, 40.1, 37.0, 35.4, 35.1, 31.8, 30.7, 30.4, 27.1, 26.7, 26.6, 26.0, 25.8, 24.6, 23.9, 20.9, 16.5, 16.0, 14.3. ESI-HRMS calcd for $C_{37}H_{60}N_3O_8^+$: 674.4380 [M+H]$^+$; found: 674.4396.

11. **Preparation of Ethereal SSG (29)**

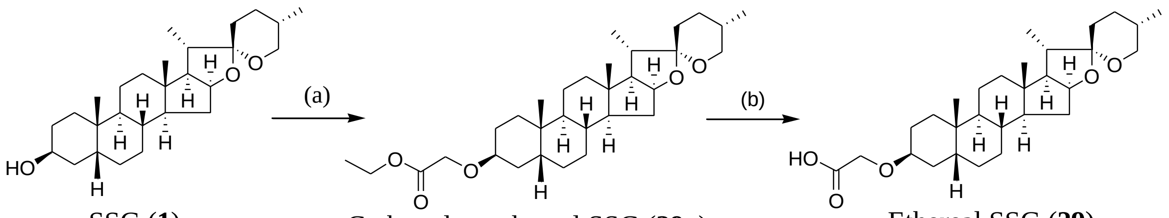

SSG (**1**) Carboxylate ethereal SSG (**29a**) Ethereal SSG (**29**)

Fig. S11: Synthesis of ethereal SSG (**29**).

(a) To a solution of SSG (42 mg, 0.1 mmol) and $Rh_2(OAc)_4$ (1 mg) in anhydrous CH_2Cl_2 (2 mL), ethyl diazoacetate (EDA, 35 mg, 0.3 mmol) dissolved in CH_2Cl_2 (3 mL) was added through a syringe pump for 3 h at 40 °C. Compound **29a** was purified for characterization. $[\alpha]_D^{25}$ -55 (*c* 0.79, 2:1 CH_2Cl_2/CH_3OH). ^{1}H NMR (400 MHz, CDCl3) δ (ppm): 4.40 (m, 1H), 4.20 (q, *J* = 7.1 Hz, 1H), 4.05 (s, 2H), 3.95 (dd, *J* = 11.0, 2.6 Hz, 1H), 3.69 (s, 1H), 3.30 (d, *J* = 11.0 Hz, 1H), 2.03-1.94 (m, 2H), 1.92-1.78 (m, 4H), 1.77-1.54 (m, 6H), 1.52-1.34 (m, 7H), 1.33-1.22 (m, 4H), 1.28 (t, *J* = 7.1 Hz, 3H), 1.21-1.10 (m, 3H), 1.08-1.02 (m, 1H), 1.07 (d, *J* = 7.1 Hz, 3H), 0.99 (d, *J* = 6.9 Hz, 3H), 0.96 (s, 3H), 0.76 (s, 3H). ^{13}C NMR (100 MHz, CDCl3) δ (ppm): 171.2, 109.7, 81.0, 75.6, 65.7, 65.1, 62.1, 60.7, 56.5, 42.1, 40.7, 40.3, 40.1, 36.8, 35.3, 35.1, 31.7, 30.5, 30.3, 27.1, 26.6, 26.5, 25.9, 25.8, 24.6, 23.8, 20.9, 16.5, 16.0, 14.3, 14.2. ESI-HRMS calcd for

$C_{31}H_{51}O_5^+$: 503.3737 [M+H]$^+$; found: 503.3735.

(b) Then, the reaction mixture from (a) was concentrated and the residue was dissolved in MeOH-H_2O (5:1, 6 mL) followed by addition of K_2CO_3 (300 mg). Refluxed the reaction mixture for ~5 h and monitored by TLC. The mixture was adjusted to pH 2 by dilute HCl, extracted with CH_2Cl_2 (3 × 20 mL) and the organic fraction was dried by $MgSO_4$, concentrated under reduced pressure. The residue was subjected to column chromatography using EtOAc/n-hexane as eluent to obtain ethereal SSG (**29**) (Yield: 80%), $[\alpha]_D^{25}$ -58 (*c* 0.41, 2:1 CH_2Cl_2/CH_3OH). ^1H NMR (600 MHz, MeOH-d_4) δ (ppm): 4.38 (m, 1H, H-16), 4.02 (d, *J* = 1.6 Hz, 2H, H-28), 3.92 (dd, *J* = 2.5 Hz, 11.0 Hz, 1H, H-26a), 3.26 (d, *J* = 11.0 Hz, 1H, H-26b), 3.70 (s, 1H, H-3), 1.82-2.04 (m, 6H), 1.60-1.75 (m, 7H), 1.38-1.56 (m, 5H), 1.10-1.37 (m, 9H), 1.08, (d, *J* = 7.0 Hz, 3H, H-27), 0.98 (d, *J* = 7.1 Hz, 3H, H-21), 0.97 (s, 3H, H-19), 0.76 (s, 3H, H-18). ^{13}C NMR (150 MHz, MeOH-d_4) δ (ppm): 174.9, 111.2, 82.6, 77.0, 66.3, 66.2, 57.8, 43.6, 42.0, 41.5, 41.4, 38.3, 36.9, 36.3, 32.8, 31.7, 31.6, 31.5, 28.7, 27.9, 27.8, 27.1, 26.9, 25.6, 24.5, 22.2, 17.1, 16.5, 14.9. EI-HRMS calcd for $C_{29}H_{46}O_5^+$: 474.3345 [M]$^+$; found:474.3333.

12. **Preparation of α-, β-OMe SSG (32, 33) and α-, β-OH SSG (34)**

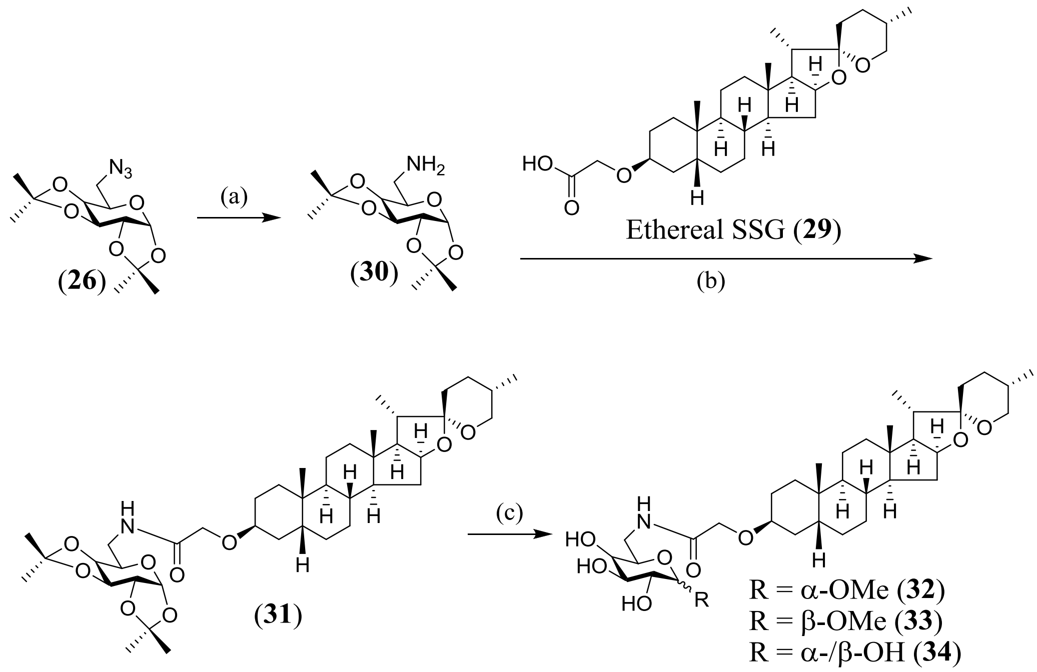

Fig. S12: Synthesis of **30**, **31**, α-, β-OMe SSG (**32-33**) and α-/β-OH SSG (**34**).

(a) Compounds **30** was prepared according to the literature.[14] Reducing of **26** with Ph_3P (1.5 eq) and H_2O (15 eq) in THF under reflux for 18 hours afforded **30** in 95% yield after purification by flash column chromatography. ^1H NMR (400 MHz, $CDCl_3$) δ (ppm): 5.55 (d, *J* = 5.0 Hz, 1H), 4.61 (dd, *J* = 7.9, 2.3 Hz, 1H), 4.47 (br s, 2H), 4.32 (dd, *J* = 5.0, 2.4 Hz, 1H), 4.23 (dd, *J* = 7.9, 1.7 Hz, 1H), 3.91-3.87 (m, 1H), 3.09-2.98 (m, 1H), 1.58 (s,

3H), 1.44 (s, 3H), 1.33 (s, 6H). ^{13}C NMR (100 MHz, $CDCl_3$) δ (ppm): 109.3, 108.7, 96.1, 71.5, 70.6, 70.4, 67.5, 41.3, 26.0, 25.9, 24.8, 24.2.

(b) To a solution of ethereal SSG (**29**) in anhydrous CH_2Cl_2 (2 mL), galactose amine **30**[14] (25 mg), *N,N'*-diisopropylcarbodiimide (DIC, 25 mg), hydroxybenzotriazole (HOBt, 5 mg) were added and the mixture was stirred at room temperature for 48 h. After concentrating the mixture in reduced pressure, the residue was purified by flash column chromatography to give product **31** (Yield: 92%). $[\alpha]_D^{25}$ -44 (*c* 0.86, CH_2Cl_2). ^{1}H NMR (400 MHz, $CDCl_3$) δ (ppm): 7.04 (t, *J* = 5.7 Hz, 1H), 5.51 (d, *J* = 4.8 Hz, 1H), 4.60 (dd, *J* = 7.9 Hz, 1.9 Hz, 1H), 4.41 (q, *J* = 7.5 Hz, 1H), 4.30 (dd, *J* = 5.0 Hz, 2.4 Hz, 1H), 4.21 (dd, *J* = 7.9 Hz, 1.8 Hz, 1H), 3.97-3.88 (m, 4H), 3.71-3.65 (m, 2H), 3.36-3.29 (m, 2H), 2.03-1.93 (m, 2H), 1.92-1.73 (m, 5H), 1.71-1.65 (m, 3H), 1.64-1.54 (m, 2H), 1.48-1.34 (m, 6H), 1.47 (s, 3H), 1.46 (s, 3H), 1.34 (s, 3H), 1.32-1.22 (m, 5H), 1.31 (s, 3H), 1.22-1.12 (m, 3H), 1.08-1.03 (m, 1H), 1.08 (d, *J* = 7.0 Hz, 3H), 0.97 (d, *J* = 6.8 Hz, 3H), 0.97 (s, 3H), 0.76 (s, 3H). ^{13}C NMR (100 MHz, $CDCl_3$) δ (ppm): 170.8, 109.7, 109.5, 108.7, 96.3, 81.0, 75.5, 71.6, 70.9, 70.6, 67.4, 66.4, 65.2, 62.1, 56.5, 42.2, 40.7, 40.3, 40.1, 39.3, 36.9, 35.3, 35.1, 31.8, 30.7, 30.4, 27.1, 26.6, 26.6, 26.1, 25.99, 25.97, 25.8, 25.1, 24.7, 24.3, 23.8, 20.9, 16.5, 16.1, 14.4. ESI-HRMS calcd for $C_{41}H_{66}NO_9^+$: 716.4738 $[M+H]^+$; found: 716.4771.

(c) The solution of **31** (18 mg) in a mixed solvent of MeOH (1 mL) and concentrated HCl (1 mL) was stirred at 40 ℃ for 3 h. Then the mixture was dried in vacuuo and the residue was purified by flash column chromatography to give three products of α-OMe SSG (**32**) (Yield: 24%), β-OMe SSG (**33**) (Yield: 16%) and a mixture of α-/β-OH SSG (**34**) (Yield: 38%). α-OMe SSG (**32**): $[\alpha]_D^{25}$ -31 (*c* 0.22, 2:1 CH_2Cl_2/CH_3OH). ^{1}H NMR (400 MHz, $CDCl_3$) δ (ppm): 7.03 (brs, 1H), 4.81 (d, *J* = 3.5 Hz, 1H), 4.40 (q, *J* = 7.2 Hz, 1H), 3.97-3.88 (m, 6H), 3.40 (s, 3H), 3.30 (d, *J* = 11.0 Hz, 1H), 2.04-1.95 (m, 2H), 1.93-1.84 (m, 3H), 1.83-1.74 (m, 2H), 1.73-1.64 (m, 3H), 1.64-1.55 (m, 2H), 1.54-1.35 (m, 6H), 1.34-1.22 (m, 5H), 1.19-1.12 (m, 3H), 1.10-1.05 (m, 1H), 1.08 (d, *J* = 7.0 Hz, 3H), 0.97 (d, *J* = 6.8 Hz, 3H), 0.97 (s, 3H), 0.76 (s, 3H). ^{13}C NMR (150 MHz, $CDCl_3$) δ (ppm): 172.2, 99.7, 81.0, 76.1, 70.5, 69.8, 68.7, 68.3, 67.8, 67.2, 65.2, 62.1, 56.4, 55.6, 42.1, 40.7, 40.3, 40.1, 38.8, 37.1, 35.3, 35.1, 31.7, 30.7, 30.4, 29.7, 27.1, 26.6, 26.5, 25.9, 25.8, 24.6, 24.0, 20.9, 16.5, 16.0, 14.3. ESI-HRMS calcd for $C_{36}H_{60}NO_9^+$: d650.4268 $[M+H]^+$; found: 650.4258. β-OMe SSG (**33**): ^{1}H NMR (400 MHz, $CDCl_3$) δ (ppm): 7.19 (brs, 1H), 4.92 (s, 1H), 4.40 (q, *J* = 7.0 Hz, 1H), 4.08-3.88 (m, 7H), 3.70 (m, 3H), 3.48 (m, 1H), 3.40 (s, 3H), 3.30 (d, *J* = 10.8 Hz, 1H), 3.03 (d, *J* = 11.0 Hz, 1H), 2.04-1.95 (m, 2H), 1.92-1.83 (m, 3H), 1.82-1.73 (m, 2H), 1.73-1.64 (m, 3H), 1.64-1.55 (m, 2H), 1.54-1.37 (m, 6H), 1.34-1.23 (m, 5H), 1.19-1.12 (m, 3H), 1.10-1.04 (m, 1H), 1.08 (d, *J* = 7.0 Hz, 3H), 0.97 (d, *J* = 6.8 Hz, 3H), 0.97 (s, 3H), 0.76 (s, 3H). ^{13}C NMR (150 MHz, $CDCl_3$) δ (ppm): 173.4, 109.8, 88.9, 81.0, 78.8, 78.7, 76.1, 72.0, 67.2, 65.2, 62.1, 56.4, 55.0, 43.8, 42.1, 40.7, 40.3, 40.1,

37.1, 35.3, 35.2, 31.7, 30.7, 30.4, 29.7, 27.1, 26.6, 26.5, 25.9, 25.8, 24.6, 24.0, 20.9, 16.5, 16.0, 14.4. ESI-HRMS calcd for $C_{36}H_{60}NO_9^+$: 650.4268 $[M+H]^+$; found: 650.4241. α-/β-OH SSG (**34**): $[\alpha]^{25}_{D}$ -10 (*c* 0.45, 2:1 CH_2Cl_2/CH_3OH). ^{1}H NMR (400 MHz, $CDCl_3$) *δ* (ppm): 7.45-7.39 (brs, 1H), 4.40 (d, *J* = 5.6 Hz, 1H), 3.30 (d, *J* = 10.2 Hz, 1H), 2.04-1.95 (m, 2H), 1.94-1.73 (m, 5H), 1.72-1.55 (m, 5H), 1.51-1.39 (m, 6H), 1.36-1.23 (m, 5H), 1.18-1.11 (m, 3H), 1.08-1.04 (m, 1H), 1.08 (d, *J* = 7.0 Hz, 3H), 0.98 (d, *J* = 6.8 Hz, 3H), 0.96 (s, 3H), 0.76 (s, 3H). ^{13}C NMR (150 MHz, $CDCl_3$) *δ* (ppm): 172.5, 109.7, 81.0, 76.1, 73.3, 72.9, 72.4, 69.4, 69.1, 68.9, 68.5, 67.3, 65.1, 62.2, 56.5, 42.2, 40.7, 40.3, 40.1, 39.5, 36.9, 35.4, 35.1, 31.8, 30.6, 30.5, 30.4, 29.7, 27.1, 26.7, 26.6, 26.0, 25.8, 24.6, 24.0, 21.0, 16.5, 16.1, 14.4. ESI-MS: *m/z* 658.6 $[M + Na]^+$. ESI-HRMS calcd for $C_{35}H_{58}NO_9^+$: 636.4112 $[M+H]^+$; found: 636.4113.

D. Tables and Figures

Table S1. Some Aβ lowering compounds from natural products

	In vitro **IC_{50} Aβ inhibition**	**Animal study**	**Mechanism**	**References**
Ginsenosides	~ 50 μM<br>in CHO expressing APPswe	APPswe transgene were orally administered with a dose of 25 mg/kg Rg3 for 18 h. Brain $A\beta_{1\text{-}42}$ was reduced by 30%	-	FASEB J. (2006) 20: E599
Epigallocatechin-3-gallate (EGCG)	~ 20 μM<br>in N2A expressing APPswe	APPswe transgene were injected with 10 mg/kg i.p. for 60 d. Brain $A\beta_{1\text{-}42}$ was reduced by 50%.	Stimulation of nonamyloidogenic processing of APP	J. Neurosci., (2005) 25:8807
Resveratrol	~ 20 μM<br>in HEK293 expressing APP	APP/PS1 transgene were fed a diet with 0.35% resveratrol for 15 weeks. Brain $A\beta_{42}$ was reduced by 20%.	Autophagy of Aβ	J. Biol. Chem. (2010) 285:9110
Curcumin	>20 μM<br>in Mouse primary cortical neurons	APPswe transgene were fed with chow with 500 ppm curcumin for 22 weeks. Amyloid deposition was reduced.	Inhibition of Ab aggregation, inhibition of APP maturation.	J. Biol. Chem. (2005) 280: 5829;<br>J. Biol. Chem. (2010) 285:28472
Omega-3 fatty acid docosahexaenoic acid (DHA)	~ 100 μM<br>in SH-SY5Y Cells expressing APP	APPswe transgene were fed a diet with 0.5% level of DHA for 5 months. Total Aβ was reduced by 70%	Stimulation of nonamyloidogenic processing of APP	J Neurosci. (2005) 25: 3032<br>J. Biol. Chem (2011) 286:14028
Huperzine A	~ 10 μM<br>in HEK293 expressing APPswe	APPswe/PS1dE9 transgenic mice were orally administered with 0.1 mg/kg for 7 months. Amyloid plaque burden and	Stimulation of nonamyloidogenic processing of APP	J Neurosci Res (2012) 92: 108

		oligomeric Aβ levels in the cortex and hippocampus were reduced.		
Stigmasterol	~ 10 μM in in SH-SY5Y Cells expressing APP	C57/B6 mice were orally administered with 0.3% stigmasterol in diet for 4-6 weeks. Ab42 and Ab40 species species were reduced by 10%.	Stimulation of nonamyloidogenic processing of APP	J Neurosci. (2013) 33: 16072

Table S2. Plasma and brain concentrations of SSG **1**, timosaponins **2-3** and compound **34** in mice (n = 6) treated with three repeated oral doses (100 mg/kg) in two days, conducted by LC-MS/MS.

Plasma			Brain		
Analyte	Precursor (μM)	Metabolite (μM)	Analyte	Precursor (μM)	Metabolite (μM)
SSG **1**	0.118 ± 0.062	Not detected	SSG **1**	1.042 ± 0.418	Not detected
TAIII **2**	0.050 ± 0.058	TAI: 0.048 ± 0.018<br>SSG: 0.035 ± 0.016	TAIII **2**	Not detected	TAI: 0.014 ± 0.008<br>SSG: 0.203 ± 0.044
TAI **3**	3.361 ± 0.925	SSG: 0.058 ± 0.016	TAI **3**	1.174 ± 0.401	SSG: 0.416 ± 0.158
**34**	0.051 ± 0.072	Not detected	**34**	0.025 ± 0.041	Not detected

Compound	Binding pose	Binding score
**SSG**	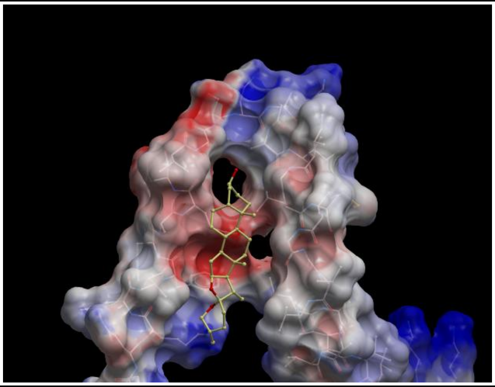	-10.11
**TA-I**	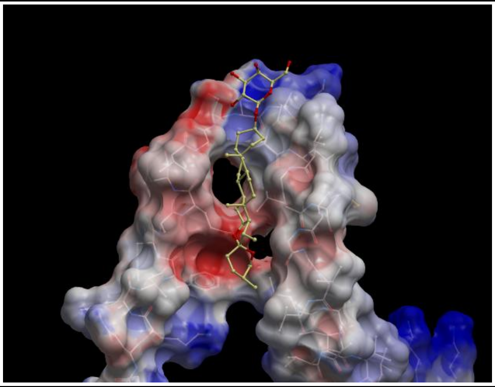	-3.26
**TA-III**	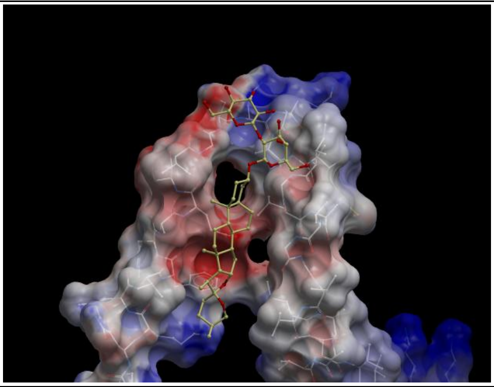	-3.08
**Cholesterol**	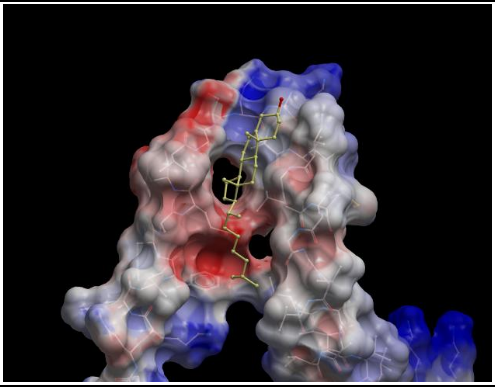	-11.20

Fig. S14: Molecular docking of SSG, TAI, TAIII and cholesterol to the transmembrane domain of APP. Electrostatic surface representation of the transmembrane region of APP, showing the energy minimized binding pose of TAIII. Positive and negative charge surfaces are presented as blue and red regions, respectively.

E. References

1. H. Fu, W. Li, J. Luo, N. T. Lee, M. Li, K. W. Tsim, Y. Pang, M. B. Youdim and Y. Han, *Biochem. Biophys. Res. Commun.*, 2008, **366**, 631-636.
2. L. Jiang and T. H. Chan, *J. Org. Chem.*, 1998, **63**, 6035-6038.
3. S. Cheng, Y. Duo, B. Ma and D. Tan, *Org. Biomol. Chem.*, 2009, **7**, 3112-3118.
4. Y. Du, G. Gu, G. Wei, Y. Hua and R. J. Lindhardt, *Org. Lett.*, 2003, **5**, 3627-3630.
5. R. S. Lankalapalli, A. Baksa, K. Liliom and R. Bittman, *ChemMedChem*, 2010, **5**, 682-686.
6. G. Gu, M. Fang, J. Liu and L. Gu, *Carbohydrate Research*, 2011, **346**, 2406-2413.
7. C. Li, B. Yu, M. Liu and Y. Hui, *Carbohydr. Res.*, 1998, **306**, 189-195.
8. Y. Li, Y. Zhang, T. Guo, H. Guan, Y. Hao and B. Yu, *Synthesis*, 2006, **5**, 775-782.
9. J. R. Williams, H. Gong, N. Hoff, O. I. Olubodun and P. J. Carroll, *Org. Lett.*, 2004, **6**, 269-271.
10. (*a*) M. A. Iglesias-Arteaga, R. P. Gill, C. S. P. Martinez and F. C. Manchado, *J. Chem. Soc., Perkin Trans*. **1**, 2001, 261–266; (*b*) M. A. Iglesias Arteaga, R. P. Gil, V. L. Lara, C. S. P. Martinez, F. C. Manchado, A. R. Perez and L. P. Rios, *Synth. Commun.*, 1998, **28**, 1381-1386; (*c*) M. A. Iglesias Arteaga, R. P. Gil, V.L. Lara, F. C. Manchado and C. S. P. Martinez, *Synth. Commun.*, 1998, **28**, 75-81.
11. S. K. Upadhyay, C. C. Creech, K. L. Bowdy, E. D. Stevens, B. S. Jursic and D. M. Neumann, *Bioorg. Med. Chem. Lett.*, 2011, **21**, 2826-2831.
12. P. J. Gunning, P. D. Tiffin, PCT Int. Appl., 2004, **WO 2004037845 A1**.
13. E. L. Eliel, V. G. Badding and M. N. Rerick, *J. Am. Chem. Soc.*, 1962, **84**, 2371-2377.
14. B. Doboszewski, E. Groaz and P. Herdewijn, *Eur. J. Org. Chem.*, 2013, 4804-4815.
